# Global impact of particulate matter on ischemic stroke

**DOI:** 10.3389/fpubh.2024.1398303

**Published:** 2024-06-05

**Authors:** Zhouyu Xie, Peng Shu, Fei Li, Yi Chen, Wangfang Yu, Ronglei Hu

**Affiliations:** ^1^Department of ICU, Tongxiang Traditional Chinese Medicine Hospital, Tongxiang, Zhejiang, China; ^2^Precision Medicine Research Center, Beilun People’s Hospital, Ningbo, Zhejiang, China; ^3^Department of Neurosurgery, Beilun People’s Hospital, Ningbo, Zhejiang, China; ^4^Department of Pathology, Tongxiang First People’s Hospital, Tongxiang, Zhejiang, China

**Keywords:** ischaemic stroke, ambient particulate matter pollution, death, DALY, sociodemographic index

## Abstract

**Objective:**

This study assesses the worldwide impact of ischemic stroke caused by ambient particulate matter pollution between 1990 and 2019, utilizing data from the Global Burden of Disease (GBD) 2019.

**Methods:**

An analysis was conducted across various subgroups, including region, Socio-demographic Index (SDI) level, country, age, and gender. The study primarily examined metrics such as death cases, death rate, Disability-Adjusted Life Years (DALYs), DALY rate, and age-standardized indicators. The Estimated Annual Percentage Change (EAPC) was calculated to assess trends over time.

**Results:**

The study found a moderate increase in the global burden of ischemic stroke attributed to ambient particulate matter, with the age-standardized DALY rate showing an EAPC of 0.41. Subgroup analyses indicated the most substantial increases in Western Sub-Saharan Africa (EAPC 2.64), East Asia (EAPC 2.77), and Eastern Sub-Saharan Africa (EAPC 3.80). Low and middle SDI countries displayed the most notable upward trends, with EAPC values of 3.36 and 3.58 for age-standardized death rate (ASDR) and DALY rate, respectively. Specifically, countries like Equatorial Guinea, Timor-Leste, and Yemen experienced the largest increases in ASDR and age-standardized DALY rate. Furthermore, both death and DALY rates from ischemic stroke due to particulate matter showed significant increases with age across all regions.

**Conclusion:**

The study highlights the increasing worldwide health consequences of ischemic stroke linked to particulate matter pollution, particularly in Asia and Africa. This emphasizes the critical necessity for tailored public health interventions in these regions.

## 1 Introduction

Ischemic stroke, caused by the blockage or narrowing of arteries in the brain, is a prevalent type of stroke resulting in tissue damage and cell death (1). It ranks as the second leading cause of death and disability (2) worldwide, with 80% of all strokes being ischemic. Furthermore, ischemic stroke plays a major role in neurological disorders and places significant financial and healthcare strains on a global level (3, 4).

Ambient particulate matter pollution refers to the suspension of small particles in the air, which have adverse effects on the environment and human health. This pollution is categorized into PM10, PM2.5, and PM0.1 based on particle size, with these particles reaching deep into the lungs and bloodstream, triggering inflammation and oxidative stress pathways that increase the risk of vascular dysfunctions (5, 6). These fine particles originate from various sources such as industrial emissions, vehicle exhaust, and construction activities (7, 8). Research indicates that ambient particulate matter pollution poses a range of health risks (9–11), including respiratory and cardiovascular issues like atherosclerosis (12), myocardial infarction, hypertension, and stroke (13–15). Recent systematic reviews and meta-analyses have further confirmed the association between particulate matter exposure and higher rates of cardiovascular diseases and mortality (16). Studies in the Middle East and North Africa have shown significant variations in disease burden related to air pollution, emphasizing the need for targeted public health interventions in the region (17). Therefore, a comprehensive understanding of the health impact of ambient particulate matter pollution is essential for developing specific prevention strategies for ischemic stroke.

The Global Burden of Disease 2019 (GBD 2019) database offers extensive data to support the creation of public health policies and measures for disease prevention and control. It serves as a valuable tool for evaluating global health status. Additionally, various studies using the GBD 2019 database have made significant contributions to analyzing disease burden, identifying development trends, shaping health policies, and refining disease prevention and control strategies (18, 19).

The current research on the disease burden of ischaemic stroke caused by air pollution is lacking in comprehensiveness. This study analyzes the global burden of ischaemic stroke attributed to ambient particulate matter pollution from 1990 to 2019 using data from the GBD 2019 database. It examines various burden of disease indicators such as death cases, death rate, Disability-Adjusted Life Years (DALYs), DALY rate, age-standardized rate, and Estimated Annual Percentage Change (EAPC). The distribution of ischaemic stroke cases linked to ambient particulate matter pollution is explored across different levels including region, Socio-demographic Index (SDI) level, country, age, and gender, while also assessing changing trends. By delving into the distribution and trends of ischaemic stroke burden due to ambient particulate matter pollution using GBD 2019 data, this study can help guide the development of evidence-based public health prevention and control strategies, offering a scientific foundation for reducing the disease burden of ischaemic stroke.

## 2 Methods

### 2.1 Study data

This study draws upon data from the ‘Global Burden of Disease (GBD) 2019 Study,’ an extensive international research initiative (20). The GBD 2019 Study is dedicated to collecting and analyzing global health data to evaluate disease burden, offering valuable insights into mortality and morbidity across different regions and demographics. Such information is crucial for the development of public health policies and disease prevention strategies. The data utilized in this study are sourced from the GBD database, which compiles detailed epidemiological patterns from health registries, official statistics, hospital records, and extensive literature reviews, encompassing 21 regions and 204 countries worldwide. It is important to note that this study is based on publicly available data and does not require ethical approval.

### 2.2 Classification coding and diagnosis of diseases

The International Classification of Diseases (ICD) codes for ischemic stroke encompass G45-G46.8, I63-I63.9, I65-I66.9, I67.2-I67.3, I67.5-I67.6, I69.3 (ICD-10), and 433–435.9, 437.0–437.1, 437.5–437.8 (ICD-9). The diagnostic process for ischemic stroke typically involves a comprehensive assessment including physical examination, neuroimaging, and cerebral angiography.

### 2.3 Subgroup analysis

The study classified subgroups based on gender and age across 21 regions and 204 countries globally. Age groups spanned from 25 to 85 years old, with each 5-year interval representing a distinct age group. Furthermore, each country was grouped into five categories based on their SDI level: High SDI, high-middle SDI, middle SDI, low-middle SDI, and Low SDI. SDI level was determined using three indicators: *per capita* income, population education level, and fertility rate, collectively offering insights into the overall developmental status of a society (21).

### 2.4 Attribution analysis of risk factors

The analysis of risk factors for disease burden utilizes the Population Attributable Fraction (PAF) method to assess the contribution of a risk factor to the total disease burden in a population. The disease burden linked to these risk factors is calculated by multiplying them with the burden of disease value. The equation for this calculation is as follows:


$$PAF=\frac{p*\left(RR-1\right)}{1+p*\left(RR-1\right)}$$

AB=BI∗PAF

The exposure rate of a specific risk factor, which refers to the proportion of the population exposed to it, is a key consideration. Relative risk, which compares the risk of disease between the exposed and non-exposed groups, is also important. Furthermore, evaluating the burden of disease attributed to the risk factor and its corresponding indicator is crucial for understanding the overall impact on public health.

### 2.5 Burden of disease indicator

The study includes various measures of disease burden, such as the number of death cases, death rate, DALYs, number of DALY cases, DALY rate, age-standardized rate (ASR), and EAPC.

Death cases refer to the total number of deaths caused by diseases within a specific time period, illustrating the direct impact of these diseases on population health. Researchers often use DALYs to measure the burden of diseases on life expectancy and quality of life, calculated by combining Years of Life Lost (YLL) and Years Lived with Disability (YLD). The death rate and DALY rate serve as standardized indicators of mortality or disease burden in a particular population over a set period, typically reported as rates per 100,000 individuals. These metrics offer insights into the overall health impact of a disease on a population’s well-being.

To enhance comparability across different spatial and temporal scales, age-standardized burden of disease indicators are computed by adjusting the ASR of relevant burden of disease indicators. This adjustment standardizes the indicators to a common age distribution, enabling evaluation in units per 100,000 individuals. The calculation methodology for age-standardized burden of disease indicators is utilized for this purpose.

The EAPC is used to evaluate the average annual rate of change in burden of disease indicators over a specific time period. It is often combined with age-standardized data to illustrate the trend of these indicators over different time periods. The calculation for EAPC is as follows:


$$ASR=\frac{\sum_{i=1}^{A}a_{i}w_{i}}{\sum_{i=1}^{A}w_{i}}\times 100,000$$

Where $a_{i}$ , denotes an indicator of disease burden in the ith age group, and $w_{i}$ denotes the corresponding standard population weight.

The EAPC, or Estimated Annual Percentage Change, is a metric employed to assess the average yearly rate of change in burden of disease indicators over a specific timeframe. It is applied to age-standardized data to illustrate the evolving trend of burden of disease indicators across various time intervals, and is calculated as follows:


$$y=\alpha +\beta x+\varepsilon ,\;y=\ln\left(\gamma \right)$$

$$\mathrm{EAPC}=100\times \left(\exp\left(\beta \right)-1\right)$$

Among them, X represents the year, γ denotes the standardization rate, and ε signifies the residual. A positive EAPC indicates an upward trend, while a negative EAPC suggests a downward trend. The significance of the trend is stronger with a larger absolute value of EAPC. A value of EAPC = 0 indicates that the indicator has remained essentially unchanged over the specified time period.

## 3 Results

### 3.1 Status and changes of disease burden of ischaemic stroke attributable to ambient particulate matter pollution in different regions of the world from 1990 to 2019

Table 1 and Supplementary Table S1 present the changes in age-standardized death rates (ASDR) and age-standardized DALY attributed to ambient particulate matter pollution globally for ischaemic Stroke between 1990 and 2019. The rates decreased from 6.71/100,000 and 130.81/100,000 in 1990 to 6.58/100,000 and 146.16/100,000 in 2019, respectively. The EAPCs were −0.05 and 0.41, respectively. In 2019, the number of deaths and DALYs attributed to ambient particulate matter pollution for ischaemic stroke were 515,957.16 × 10 and 119,303,172.03 × 10, respectively. These figures show an increase compared to 1990, where the death cases were 225,607.43 × 103 and DALYs were 4,942,155.46 × 103, resulting in growth rates of 2.29 and 2.41, respectively.

**Table 1:** ASDR, age-standardized DALY rate and EAPC of ischaemic stroke Attributed to ambient particulate matter pollution in regions of the world from 1990 to 2019.

Region	ASDR			Age-standardized DALY rate		
	1990 (95% UI)	2019 (95% UI)	EAPC (95% CI)	1990 (95% UI)	2019 (95% UI)	EAPC (95% CI)
Global	6.71 (4.77, 8.65)	6.58 (5.47, 7.71)	−0.05 (−0.11, 0.01)	130.81 (93.18, 169.76)	146.16 (119.78, 171.23)	0.41 (0.35, 0.46)
Andean Latin America	3.41 (1.56, 5.94)	2.58 (1.78, 3.58)	−0.91 (−1.26, −0.56)	66.05 (30.49, 115.37)	52.44 (36.71, 71.07)	−0.81 (−1.16, −0.47)
Australasia	1.39 (0.16, 3.45)	0.36 (0.09, 0.68)	−5.34 (−5.66, −5.02)	24.82 (2.72, 60.97)	7.17 (1.77, 13.58)	−4.77 (−5.11, −4.44)
Caribbean	3.73 (1.58, 6.77)	3.52 (1.93, 5.65)	−0.06 (−0.21, 0.09)	72.55 (30.72, 132.26)	70.36 (39.15, 111.60)	0.05 (−0.10, 0.21)
Central Asia	9.11 (4.66, 15.40)	12.16 (8.70, 16.10)	0.61 (0.29, 0.93)	197.56 (100.90, 330.70)	255.08 (181.80, 339.10)	0.48 (0.17, 0.80)
Central Europe	16.31 (8.85, 24.70)	8.12 (6.58, 9.85)	−2.73 (−3.00, −2.45)	317.24 (176.10, 472.77)	157.66 (128.77, 189.53)	−2.71 (−2.96, −2.47)
Central Latin America	3.94 (1.97, 6.64)	2.27 (1.68, 2.88)	−2.25 (−2.45, −2.04)	77.61 (39.76, 128.16)	46.45 (35.31, 58.59)	−2.11 (−2.33, −1.89)
Central sub-Saharan Africa	1.81 (0.60, 4.11)	3.60 (1.79, 6.27)	2.16 (1.70, 2.63)	35.39 (11.80, 81.53)	70.84(35.51,119.26)	2.20 (1.72, 2.69)
East Asia	6.76 (3.24, 11.40)	13.52 (10.98, 16.18)	2.77 (2.39, 3.16)	140.66 (68.08, 236.36)	288.24 (234.23, 344.52)	2.81 (2.50, 3.12)
Eastern Europe	18.79 (8.54, 31.11)	7.51 (4.67, 10.68)	−3.62 (−4.14,−3.09)	360.91 (167.77, 588.04)	151.21 (93.85, 211.80)	−3.43 (−3.94, −2.90)
Eastern Sub-Saharan Africa	0.82 (0.30, 1.82)	2.12 (1.11, 3.50)	3.80(3.63,3.98)	16.60(5.90,37.17)	41.96 (22.57, 70.99)	3.71 (3.54, 3.88)
High-income Asia Pacific	5.31 (2.03, 9.64)	1.60 (1.13, 2.13)	−4.77 (−5.18, −4.37)	104.50 (42.29, 184.95)	43.37(31.89,57.96)	−3.55 (−3.91, −3.19)
High-income North America	2.20 (0.81, 4.16)	0.49 (0.26, 0.77)	−5.80 (−6.14, −5.45)	50.29 (18.80, 93.84)	14.26 (7.45, 22.34)	−4.62 (−4.86, −4.39)
North Africa and Middle East	10.56 (8.08, 13.50)	13.31 (11.15, 15.77)	0.96 (0.76, 1.16)	226.98 (176.72, 286.01)	296.47 (249.17, 349.14)	1.07 (0.88, 1.26)
Oceania	0.93 (0.29, 2.32)	1.37 (0.45, 2.96)	1.12 (0.96, 1.28)	22.00 (6.85, 53.85)	33.03 (11.21, 73.29)	1.24 (1.09, 1.39)
South Asia	3.25 (1.37, 6.17)	6.48 (4.83, 8.33)	2.45 (2.30, 2.61)	60.64 (25.60, 114.51)	128.00 (95.09, 163.12)	2.72 (2.61, 2.84)
Southeast Asia	4.22 (1.92, 7.56)	7.04 (5.19, 8.93)	1.95 (1.62, 2.29)	90.80 (41.98, 161.31)	149.90 (111.66, 190.77)	1.85 (1.56, 2.13)
Southern Latin America	4.34 (1.78, 7.83)	2.27 (1.60, 3.03)	−2.32 (−2.58, −2.06)	83.56(35.52,147.28)	44.65 (31.38, 59.28)	−2.24 (−2.49, −1.99)
Southern sub-Saharan Africa	4.59 (3.22, 6.21)	6.96 (5.28, 8.69)	1.69 (1.22, 2.17)	98.76 (70.72, 131.51)	138.92 (106.50, 172.81)	1.41 (0.98, 1.84)
Tropical Latin America	5.09 (2.34, 9.15)	2.27 (1.53, 3.04)	−2.84 (−3.06, −2.63)	102.17 (47.48, 182.58)	44.93 (31.39, 60.14)	−2.95 (−3.14, −2.76)
Western Europe	6.25 (2.86, 10.49)	1.19 (0.85, 1.56)	−5.97 (−6.10, −5.83)	106.02 (48.17, 178.80)	22.36(16.19,29.74)	−5.49 (−5.61, −5.36)
Western sub-Saharan Africa	2.34 (1.08, 4.49)	4.87 (3.19, 6.83)	2.64 (2.42, 2.87)	45.98 (20.75, 89.04)	97.64 (63.63, 137.44)	2.72 (2.50, 2.94)

**Figure 1:**
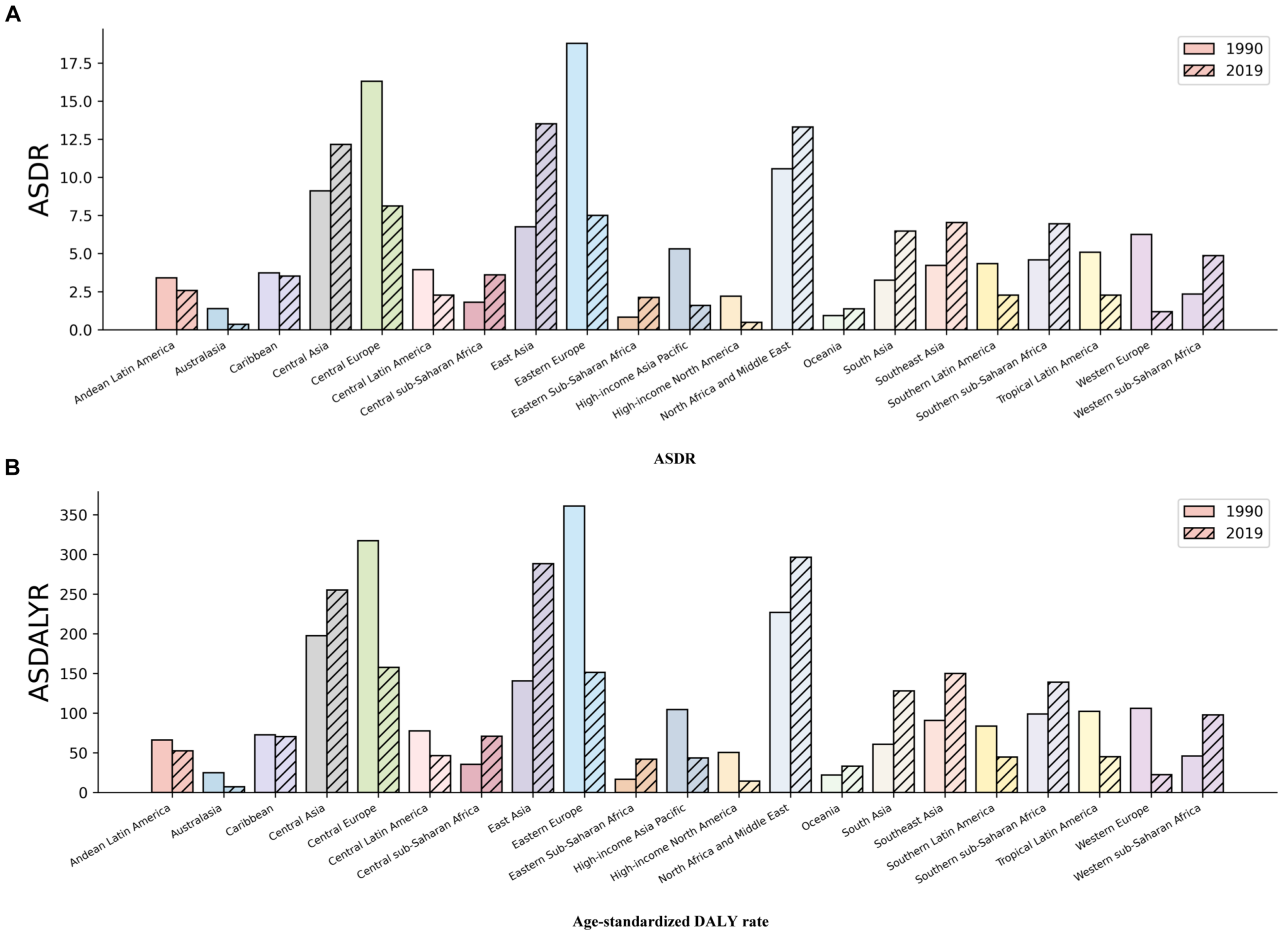
Disease Burden Metrics for Ischaemic Stroke Attributed to Ambient Particulate Matter Pollution in Regions of World in 1990 and 2019: **(A)** ASDR **(B)** Age-standardized DALY rate.

The geographic regions with the highest age-standardized DALY rates and ASDR for ischemic stroke caused by ambient particulate matter pollution in 1990 were Eastern Europe, Central Europe, and North Africa and the Middle East. In 2019, the regions with the highest rates were Central Asia, East Asia, and North Africa and the Middle East. These results are detailed in Table 1 and Figure 1.

The growth trend indicators presented in Table 1 show that Western Sub-Saharan Africa (2.64), East Asia (2.77), and Eastern Sub-Saharan Africa (3.80) had the highest EAPC of ASDR between 1990 and 2019, indicating a significant increase in ASDR. These regions also had the highest EAPC values for age-standardized DALY rates, with 2.72, 2.81, and 3.71, respectively. On the other hand, Western Europe (−5.97), High-income North America (−5.80), and Australasia (−5.34) showed the most pronounced downward trend in ASDR, reflecting the lowest EAPC values. These regions also had the lowest EAPC values for age-standardized DALY rates globally, namely, −5.49, −4.77, and −4.62, respectively. Additionally, Eastern Saharan Africa had the highest growth rate in death cases (5.75) and DALYs (5.54) compared to the year 1990.

### 3.2 Global disease burden of ischaemic stroke attributable to ambient particulate matter pollution in countries with different SDI levels and its changes from 1990 to 2019

**Figure 2:**
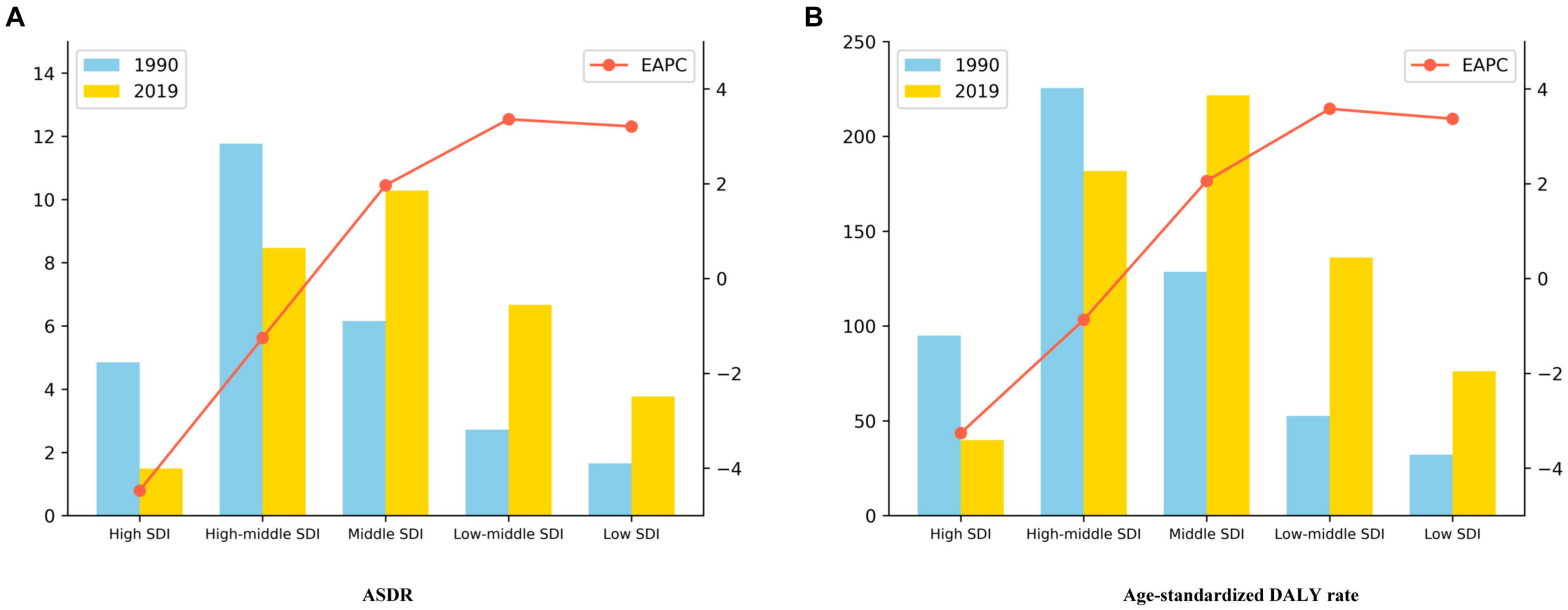
Disease Burden Metrics for Ischaemic Stroke Attributed to Ambient Particulate Matter Pollution in Countries with Different Levels of SDI Worldwide in 1990 and 2019: **(A)** ASDR **(B)** Age-standardized DALY rate.

Table 2 and Supplementary Table S2, along with Figure 2, display the ASDR and age-standardized DALY related to ischemic stroke caused by ambient particulate matter pollution across countries with varying SDI levels globally from 1990 to 2019. The data includes statistics on mortality rates, DALYs, trends in change, and percentage increase. In 1990, countries with high-middle SDI exhibited the highest ASDR (11.77/100,000) and age-standardized DALY rate (225.53/100,000). By 2019, countries with middle SDI showed higher ASDR (10.28/100,000) and age-standardized DALY rate (221.61/100,000) compared to other nations. Throughout both 1990 and 2019, high-middle SDI and middle SDI countries reported the highest number of death cases and DALYs, respectively, among all SDI categories. Over the period from 1990 to 2019, high-middle SDI and high SDI countries displayed a decreasing trend in ASDR and age-standardized DALY rate, with EAPC values below zero. Conversely, countries with middle SDI, low-middle SDI, and low SDI showed an increasing trend, with EAPC values above zero. Specifically, high SDI countries had EAPC values of −4.47 and −3.26 for ASDR and age-standardized DALY rate, respectively. In contrast, countries with low and middle SDI had EAPC values of 3.36 and 3.58 for ASDR and age-standardized DALY rate, respectively. Moreover, only high SDI countries experienced a reduction in death cases and DALYs in 2019 compared to 1990, with an increase proportion of less than 1. Conversely, countries with middle SDI, low-middle SDI, and low SDI exhibited higher increase proportions and more pronounced rises in death cases and DALYs.

**Table 2:** ASDR, age-standardized DALY rate and EAPC of ischaemic stroke attributed to ambient particulate matter pollution in countries with different SDI levels of the world from 1990 to 2019.

SDI	ASDR			Age-standardized DALY rate		
	1990 (95% UI)	2019 (95% UI)	EAPC (95% CI)	1990 (95% UI)	2019 (95% UI)	EAPC (95% CI)
High SDI	4.85 (3.10, 6.87)	1.49 (1.13,1.88)	−4.47 (−4.65, −4.28)	94.92 (61.97, 131.63)	39.87 (31.24, 49.86)	−3.26 (−3.42, −3.11)
High-middle SDI	11.77 (8.00, 15.73)	8.47 (6.97,9.96)	−1.25 (−1.44, −1.07)	225.53 (153.90, 296.92)	181.73 (151.26, 213.08)	−0.87 (−1.03, −0.71)
Middle SDI	6.16 (3.85, 8.92)	10.28 (8.46,12.10)	1.97 (1.72, 2.22)	128.56 (80.32, 186.41)	221.61 (183.79, 260.05)	2.06 (1.86, 2.27)
Low-middle SDI	2.72 (1.27, 4.91)	6.67 (4.77,8.56)	3.36 (3.26, 3.45)	52.57 (24.04, 95.34)	136.07 (97.18, 174.23)	3.58 (3.48, 3.67)
Low SDI	1.65 (0.60, 3.62)	3.77 (2.32,5.69)	3.21 (3.02, 3.39)	32.11 (11.31, 72.12)	76.17 (47.00, 114.52)	3.37 (3.17, 3.58)

### 3.3 Status and changes of disease burden of ischaemic stroke attributable to ambient particulate matter pollution in all countries from 1990 to 2019

**Figure 3:**
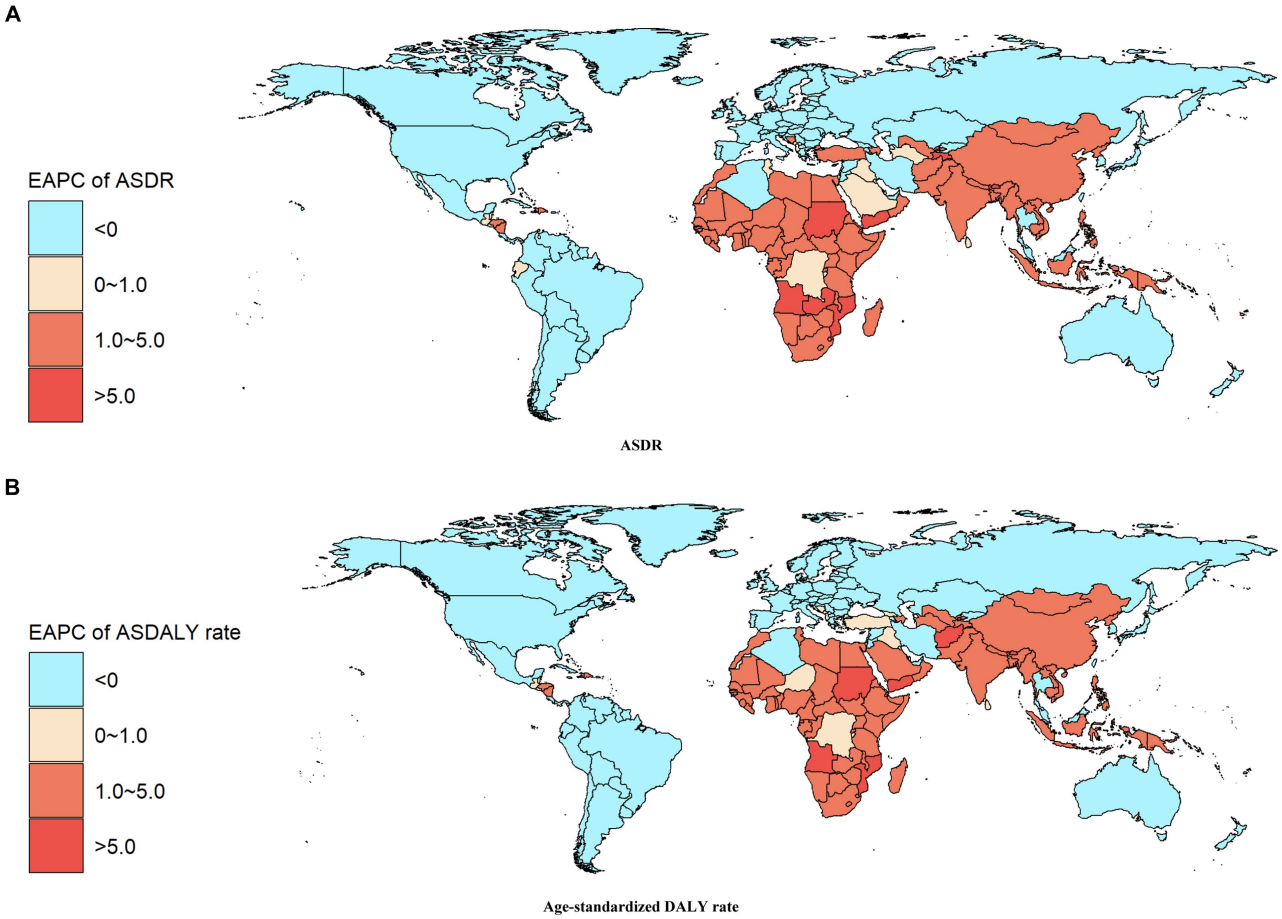
EAPC Heat Map of Disease Burden Metrics for Ischaemic Stroke Attributed to Ambient Particulate Matter Pollution in the World from 1990 to 2019: **(A)** ASDR **(B)** Age-standardized DALY rate.

The distribution of ASDR and age-standardized DALY rate due to ischaemic stroke, attributed to ambient particulate matter pollution, is illustrated in Figure 3 and Supplementary Table S3 of the European Association of Palliative Care map. Countries in Africa, South Asia, and East Asia show an increasing trend in ischaemic stroke burden from 1990 to 2019, linked to such pollution. In 2019, North Macedonia, Iraq, and Saudi Arabia had the highest ASDR rates of 33.35/100000, 26.00/100000, and 20.11/100000, respectively. Similarly, Iraq, North Macedonia, and Egypt had the highest age-standardized DALY rates globally, with rates of 542.49/100000, 528.84/100000, and 487.24/100000, respectively. Equatorial Guinea, Timor-Leste, and Yemen exhibited the most significant increases in both ASDR and age-standardized DALY rates for ischaemic stroke due to ambient particulate matter pollution. The EAPC values for ASDR were 7.81, 6.46, and 6.14, respectively, while the EAPC values for age-standardized DALY rate were 7.65, 6.44, and 6.41, respectively. Additionally, Estonia showed the most substantial downward trend in both ASDR and age-standardized DALY rate, with EAPC values of −9.39 and −8.78, respectively.

**Figure 4:**
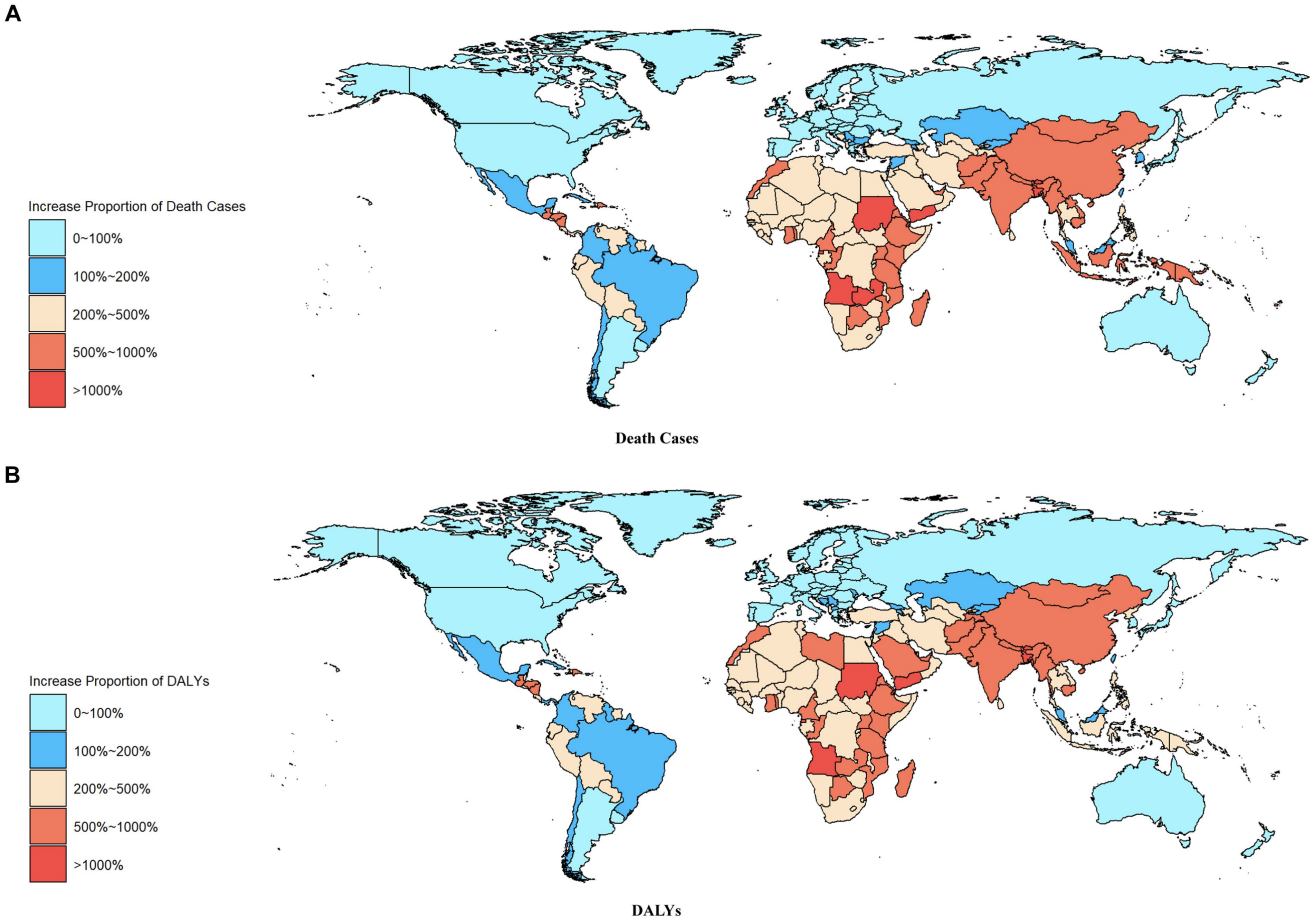
Increase Proportion Heat Map of Disease Burden Metrics for Ischaemic Stroke Attributed to Ambient Particulate Matter Pollution in the World from 1990 to 2019: **(A)** Death Cases **(B)** DALYs.

The distribution of death cases and DALYs growth in countries worldwide can be observed in Figure 4 and Supplementary Table S4. It is clear that countries showing an increase in death cases and DALYs related to ambient particulate matter pollution from ischemic stroke between 1990 and 2019 are mainly located in Africa, South Asia, East Asia, and Latin America. In 2019, China, India, Russian Federation, Indonesia, and Egypt stood out as the top five countries with the highest number of death cases and DALYs globally. Among these countries, Djibouti (16.68), Timor-Leste (16.07), and Equatorial Guinea (15.61) reported the highest percentage increase in death cases from 1990 to 2019. Similarly, Equatorial Guinea (15.29), Djibouti (15.25), and Yemen (14.40) showed the highest proportion of DALYs increase during the same period. Notably, Estonia demonstrated the lowest percentage increase in both death cases and DALYs, at just 0.13.

### 3.4 Age distribution of disease burden of ischaemic stroke attributable to ambient particulate matter pollution in countries around the world in 2019

Supplementary Tables S5, S6 present the death rate and DALY rate of ischaemic stroke caused by ambient particulate matter pollution in various age groups worldwide in 2019. The data clearly shows a notable rise in both the death rate and DALY rate of ischaemic stroke linked to ambient particulate matter pollution as age groups progressed. The impact of this disease burden was particularly high among the elderly population, exceeding that seen in younger age groups.

### 3.5 Distribution of disease burden of ischaemic stroke attributable to ambient particulate matter pollution in different genders from 1990 to 2019

Supplementary Tables S7, S8 depict the disease burden of ischaemic stroke associated with ambient particulate matter pollution, broken down by gender, geographical regions, and levels of SDI. Globally, from 1990 to 2019, the proportion of deaths and DALYs due to ischaemic stroke in men and women exhibited a similar trend, showing a noticeable increase over time. However, when examining different regions, a reduction in cases and DALYs was noted among men and women in Australasia, Central Europe, Eastern Europe, High-income Asia Pacific, High-income North America, and Western Europe. Conversely, Central sub-Saharan Africa, East Asia, Eastern Sub-Saharan Africa, and South Asia experienced higher growth rates and a greater disease burden. Moreover, over the study period, all disease burden metrics for ischaemic stroke linked to ambient particulate matter pollution decreased significantly for both genders as national SDI levels rose. Interestingly, in countries with high SDI levels, the rise in death cases and DALYs for males and females was less than 1.

## 4 Discussion

This study utilizes the extensive GBD 2019 database to investigate the relationship between ambient particulate matter pollution and the worldwide occurrence of ischemic stroke. The data was carefully categorized into different subgroups, such as regions, SDI levels, countries, genders, and age groups. Key metrics such as incidence cases, incidence rates, DALYs, DALY rate, and age-standardized indicators were thoroughly analyzed, with a particular emphasis on the EAPC.

Our findings support existing research (22), showing a substantial worldwide impact of ambient particulate matter pollution on ischemic stroke, with notable variations in geographical and demographic patterns. The analysis by SDI subgroups indicates that lower-income and developing nations experience a greater burden, with clear upward trends in ischemic stroke rates attributed to particulate matter pollution, particularly evident through higher EAPC values. Significantly, countries with lower SDI levels saw the most substantial rise from 1990 to 2019, emphasizing the urgent necessity for tailored health interventions in these areas.

Countries such as China, which have a high-middle SDI and large population sizes, exhibit data disparities resulting from demographic biases. This indicates that population structure plays a significant role in determining disease burden outcomes. Consequently, it is crucial to customize prevention and treatment strategies according to the unique conditions of each country while considering global trends (23).

Additionally, the exposure to ambient particulate matter pollution is a major contributor to health disparities (7). Countries with low levels of social development often lack resources and have weak national environmental governance, leading to higher levels of exposure to particulate matter pollution for their residents. On the other hand, developed countries with stronger economies, better access to medical resources, and comprehensive social care systems tend to have residents who are more informed and empowered to seek treatment for diseases like ischemic stroke. Recent advancements in treatment technologies have resulted in improved treatment programs and outcomes for ischemic stroke patients (7, 24–28), enhancing their quality of life. Disparities in medical resources between developed and underdeveloped regions contribute to differences in disease burden, with residents in countries with more resources having better access to advanced treatment methods, ultimately reducing the impact of the disease.

The study also revealed that the elderly population bears a disproportionate burden of ischemic stroke caused by particulate matter, as both the death rate and DALY rate increase significantly with age. The death rate and DALY rate in different age groups across countries showed a significant increase with age, aligning with previous research findings (29). The elderly population emerged as the primary group affected by this burden, likely due to weakened immunity and reduced resistance to external environmental stimuli that accompany aging (30). Their respiratory and cardiovascular systems are particularly vulnerable to damage from ambient particulate matter pollution, increasing their susceptibility to cardiovascular diseases such as arteriosclerosis and hypertension (31), which are also risk factors for ischaemic stroke (32). Therefore, targeted and timely interventions are essential for this population. Regular disease monitoring and health assessments can help identify individuals who would benefit from such interventions.

While our analysis provides a comprehensive overview using GBD 2019 data, limitations persist. Some countries may have limited high-quality epidemiological data due to constraints in data sources and collection methods, especially in countries with low SDI. While the GBD database utilizes statistical techniques to address incomplete data from low SDI countries (33), the influence of SDI may still introduce bias into disease burden analysis results. Additionally, this study does not thoroughly explore the connection between ambient particulate matter pollution and ischemic stroke, indicating a need for further investigation in future studies.

This study conducted a comprehensive analysis at multiple levels, including region, SDI level, country, age, and gender, to examine the distribution and changing trend of the global disease burden of ischemic stroke attributable to ambient particulate matter pollution from 1990 to 2019. The findings of this study have significant implications for the development of more effective health policies and interventions. Furthermore, they provide important data support for different countries and regions to share experiences and collaborate in addressing global health challenges. Future research should aim to investigate various influencing factors in order to develop more precise prevention and control strategies.

In light of our findings, future research should focus on detailed epidemiological studies to further elucidate the mechanisms by which PM2.5 contributes to ischemic stroke, especially in regions with high pollution levels and diverse sources of particulate matter. Longitudinal studies investigating the long-term health effects of sustained exposure to different components of PM2.5 are also necessary to guide targeted interventions. Healthcare systems should consider incorporating environmental health assessments into routine care, particularly in areas with high air pollution levels. From a policy perspective, it is crucial for governments and international organizations to enforce stricter air quality regulations and promote public health initiatives aimed at reducing exposure to harmful particulate matter. Collaborative efforts involving policymakers, public health officials, and researchers are essential to develop comprehensive strategies that address the complexities of air pollution and its significant impact on global health.

## 5 Conclusion

This study offers a detailed analysis of the global and regional impact of particulate matter pollution on ischemic stroke, using data from GBD 2019. The results indicate a notable increase in stroke burden, especially in regions with high pollution levels and potentially limited public health resources. The elderly population is particularly affected, underscoring the importance of targeted prevention and healthcare interventions. While the GBD 2019 data is robust, limitations related to data completeness and quality in lower SDI countries may impact the accuracy of our findings. Future research should focus on understanding the specific mechanisms through which PM2.5 contributes to stroke, and health policies should be adapted to address the varying burden of stroke worldwide. Effective strategies are likely to involve integrating environmental health considerations into broader public health initiatives and implementing tailored preventive measures based on local pollution sources and population vulnerabilities.

## Data availability statement

The original contributions presented in the study are included in the article/Supplementary material, further inquiries can be directed to the corresponding author.

## Ethics statement

Ethical approval was not required for the studies involving humans because the data is publicly available. The studies were conducted in accordance with the local legislation and institutional requirements. Written informed consent for participation was not required from the participants or the participants' legal guardians/next of kin in accordance with the national legislation and institutional requirements because the data is publicly available.

## Author contributions

ZX: Writing – original draft, Writing – review & editing. PS: Writing – original draft, Writing – review & editing. FL: Writing – review & editing. YC: Writing – review & editing. WY: Writing – review & editing. RH: Writing – review & editing.
