# Supplementary material for: Global impact of particulate matter on ischemic stroke
Source: Front Public Health. 2024 Jun 5;12:1398303. doi: 10.3389/fpubh.2024.1398303 (PMC11188470; doi:10.3389/fpubh.2024.1398303)
Supplement: Supplementary file 1 [file Table_1.DOCX]

**Supplementary Table 1 Death Cases, DALYs and Increased Proportion of Ischaemic Stroke Attributed to Ambient Particulate Matter Pollution in Regions of the World from 1990 to 2019**

| **Region** | **Death Cases** | | | **DALYs** | | |
| --- | --- | --- | --- | --- | --- | --- |
|  | **1990 (×10^3^)** | **2019 (×10^3^)** | **Increased Proportion** | **1990 (×10^3^)** | **2019 (×10^3^)** | **Increased Proportion** |
| Global | 225607.43 | 515957.16 | 2.29 | 4942155.46 | 11903172.03 | 2.41 |
| Andean Latin America | 598.84 | 1369.71 | 2.29 | 13196.43 | 29145.48 | 2.21 |
| Australasia | 313.39 | 207.58 | 0.66 | 5835.67 | 3586.08 | 0.61 |
| Caribbean | 904.02 | 1817.14 | 2.01 | 18780.4 | 36229.78 | 1.93 |
| Central Asia | 3814.26 | 7142.12 | 1.87 | 90403.13 | 180180.46 | 1.99 |
| Central Europe | 22169.95 | 18636.18 | 0.84 | 458750.58 | 343737.14 | 0.75 |
| Central Latin America | 2732.59 | 5012.38 | 1.83 | 62913.45 | 107581.36 | 1.71 |
| Central sub-Saharan Africa | 266.62 | 1302.3 | 4.88 | 6908.13 | 32977.61 | 4.77 |
| East Asia | 46106.26 | 249112.06 | 5.4 | 1163437.67 | 5834918.32 | 5.02 |
| Eastern Europe | 48560.88 | 26592.79 | 0.55 | 995405.38 | 522031.98 | 0.52 |
| Eastern Sub-Saharan Africa | 433.31 | 2491.65 | 5.75 | 11198.76 | 62081.35 | 5.54 |
| High-income Asia Pacific | 9520.4 | 9315.95 | 0.98 | 203880.37 | 192258.74 | 0.94 |
| High-income North America | 8257.95 | 3441.29 | 0.42 | 181526.1 | 86329.77 | 0.48 |
| North Africa and Middle East | 14372.46 | 48363.94 | 3.37 | 377154.95 | 1277204.47 | 3.39 |
| Oceania | 19.04 | 68.82 | 3.61 | 621.3 | 2236.4 | 3.6 |
| South Asia | 12572.96 | 75391.98 | 6 | 302887.5 | 1724055.48 | 5.69 |
| Southeast Asia | 8231.53 | 34991.92 | 4.25 | 217333.65 | 863419.17 | 3.97 |
| Southern Latin America | 1860.14 | 1963.97 | 1.06 | 38027.9 | 37617.29 | 0.99 |
| Southern sub-Saharan Africa | 1040.84 | 3123.21 | 3 | 26667.85 | 73339.36 | 2.75 |
| Tropical Latin America | 3868.83 | 5161.34 | 1.33 | 90649.07 | 106559.22 | 1.18 |
| Western Europe | 38431.16 | 13739.44 | 0.36 | 639042.82 | 215030.52 | 0.34 |
| Western sub-Saharan Africa | 1531.97 | 6711.39 | 4.38 | 37534.36 | 172652.06 | 4.6 |

**Supplementary Table 2 Death Cases, DALYs and Increased Proportion of Ischaemic Stroke Attributed to Ambient Particulate Matter Pollution in Countries with Different SDI Levels of the World from 1990 to 2019**

| **SDI** | **Death Cases** | | | **DALYs** | | |
| --- | --- | --- | --- | --- | --- | --- |
|  | **1990 (×10^3^)** | **2019 (×10^3^)** | **Increased Proportion** | **1990 (×10^3^)** | **2019 (×10^3^)** | **Increased Proportion** |
| High SDI | 52062.03 | 31751.91 | 0.61 | 1007116.45 | 710712.1 | 0.71 |
| High-middle SDI | 109606.65 | 170274.55 | 1.55 | 2327337.68 | 3695518.75 | 1.59 |
| Middle SDI | 49247.9 | 221282.17 | 4.49 | 1251272.77 | 5328904.36 | 4.26 |
| Low-middle SDI | 11847.71 | 77375.89 | 6.53 | 286211.78 | 1790952.2 | 6.26 |
| Low SDI | 2758.82 | 15098.65 | 5.47 | 68468.47 | 373080.16 | 5.45 |

**Supplementary Table 3 ASDR, Age-standardized DALY rate and EAPC of Ischaemic Stroke Attributed to Ambient Particulate Matter Pollution in Countries of the World from 1990 to 2019**

| **Country** | **ASDR** | | | **Age-standardized DALY rate** | | |
| --- | --- | --- | --- | --- | --- | --- |
|  | **1990 (95% UI)** | **2019 (95% UI)** | **EAPC (95% CI)** | **1990 (95% UI)** | **2019 (95% UI)** | **EAPC (95% CI)** |
| Afghanistan | 2.56(0.63,6.85) | 8.25(3.59,16.12) | 4.80(4.29,5.31) | 51.10(12.12,137.14) | 176.77(78.63,336.75) | 5.11(4.60,5.63) |
| Albania | 3.34(1.55,5.69) | 3.34(2.29,4.70) | 0.94(0.56,1.33) | 69.80(33.16,117.36) | 71.57(51.46,97.16) | 0.95(0.61,1.29) |
| Algeria | 15.95(9.87,23.12) | 14.71(9.67,20.18) | -0.40(-0.66,-0.14) | 284.84(178.88,409.03) | 282.89(192.95,384.22) | -0.18(-0.45,0.09) |
| American Samoa | 1.53(0.49,3.87) | 1.16(0.45,2.30) | -1.39(-1.67,-1.10) | 37.98(12.26,97.88) | 30.88(12.38,59.63) | -1.11(-1.36,-0.86) |
| Andorra | 2.13(0.64,4.09) | 0.61(0.31,0.98) | -4.10(-4.25,-3.96) | 39.55(12.16,74.02) | 12.62(6.88,19.51) | -3.77(-3.93,-3.62) |
| Angola | 1.21(0.38,2.93) | 5.06(2.43,8.58) | 5.14(4.89,5.40) | 25.11(7.87,62.51) | 102.28(49.89,170.82) | 5.05(4.76,5.34) |
| Antigua and Barbuda | 5.28(1.41,10.84) | 3.96(1.44,7.11) | -1.44(-1.81,-1.07) | 98.11(26.35,199.17) | 71.26(26.53,125.47) | -1.47(-1.81,-1.14) |
| Argentina | 3.97(1.54,7.43) | 1.78(1.09,2.56) | -2.94(-3.31,-2.57) | 76.03(30.27,140.21) | 36.03(22.50,51.56) | -2.76(-3.09,-2.42) |
| Armenia | 12.42(5.81,20.52) | 8.71(5.83,11.87) | -1.52(-1.90,-1.14) | 250.40(118.90,408.03) | 177.19(121.77,239.15) | -1.37(-1.70,-1.03) |
| Australia | 1.42(0.17,3.48) | 0.36(0.09,0.68) | -5.40(-5.73,-5.07) | 25.35(2.87,62.34) | 7.29(1.79,13.72) | -4.78(-5.13,-4.43) |
| Austria | 8.09(3.94,13.20) | 1.00(0.71,1.31) | -7.54(-7.74,-7.35) | 136.55(67.42,220.43) | 21.68(15.82,28.57) | -6.50(-6.68,-6.32) |
| Azerbaijan | 4.23(1.96,7.42) | 9.75(5.68,14.83) | 3.78(3.22,4.34) | 100.17(46.36,173.41) | 185.60(110.53,280.09) | 2.73(2.28,3.19) |
| Bahamas | 4.33(1.13,8.87) | 2.93(0.82,5.81) | -1.53(-1.71,-1.34) | 86.35(23.79,175.79) | 59.24(16.60,115.42) | -1.42(-1.57,-1.27) |
| Bahrain | 16.67(13.04,21.15) | 9.85(7.66,13.21) | -1.59(-1.98,-1.20) | 324.03(256.45,401.56) | 188.65(151.54,238.11) | -1.80(-2.08,-1.51) |
| Bangladesh | 2.73(0.87,6.07) | 8.52(5.13,12.77) | 4.35(3.92,4.79) | 47.37(14.73,108.26) | 145.07(88.37,217.11) | 4.28(4.01,4.55) |
| Barbados | 8.30(2.31,16.11) | 5.19(2.20,8.90) | -2.04(-2.46,-1.62) | 146.27(41.88,278.85) | 93.95(40.87,160.37) | -1.89(-2.29,-1.49) |
| Belarus | 14.46(7.21,22.99) | 8.13(5.33,11.48) | -2.27(-2.80,-1.73) | 307.70(158.69,476.68) | 170.45(113.78,234.41) | -2.32(-2.87,-1.76) |
| Belgium | 6.37(2.97,10.58) | 1.28(0.94,1.68) | -5.54(-5.74,-5.33) | 110.16(51.64,181.49) | 24.91(18.58,32.29) | -5.07(-5.29,-4.85) |
| Belize | 2.43(0.56,5.79) | 3.07(1.15,5.75) | 0.15(-0.39,0.70) | 45.26(10.46,104.31) | 61.69(23.80,112.18) | 0.39(-0.12,0.90) |
| Benin | 1.54(0.51,3.56) | 2.82(1.30,4.91) | 1.93(1.60,2.27) | 30.73(9.78,72.14) | 56.20(25.94,100.14) | 1.92(1.58,2.27) |
| Bermuda | 2.62(0.66,6.76) | 0.60(0.12,1.21) | -5.56(-5.93,-5.19) | 49.16(12.19,123.20) | 12.21(2.38,23.75) | -5.24(-5.63,-4.86) |
| Bhutan | 1.03(0.29,2.60) | 4.06(2.41,6.00) | 5.77(5.42,6.12) | 19.34(5.20,49.91) | 75.22(45.24,110.45) | 5.75(5.40,6.10) |
| Bolivia (Plurinational State of) | 4.70(1.65,9.09) | 4.06(2.27,6.31) | -0.71(-1.22,-0.19) | 88.68(31.28,172.30) | 74.59(43.19,113.20) | -0.82(-1.33,-0.31) |
| Bosnia and Herzegovina | 13.57(6.76,22.02) | 17.48(13.07,22.30) | 1.18(0.63,1.74) | 279.19(139.85,446.39) | 331.21(252.57,424.16) | 0.78(0.29,1.27) |
| Botswana | 4.40(2.13,7.71) | 9.54(5.72,14.08) | 2.57(2.06,3.08) | 85.62(41.71,151.00) | 183.02(114.70,264.58) | 2.47(1.95,2.99) |
| Brazil | 5.16(2.38,9.32) | 2.27(1.54,3.05) | -2.90(-3.11,-2.68) | 103.67(48.20,184.96) | 44.89(31.44,60.29) | -3.00(-3.19,-2.81) |
| Brunei Darussalam | 3.50(1.33,7.11) | 1.38(0.40,2.54) | -3.10(-3.78,-2.41) | 74.47(28.84,148.27) | 29.96(8.77,54.16) | -3.04(-3.77,-2.30) |
| Bulgaria | 20.54(10.84,31.35) | 15.75(12.12,20.05) | -0.96(-1.18,-0.74) | 388.92(212.55,580.16) | 297.47(228.98,375.25) | -0.98(-1.18,-0.79) |
| Burkina Faso | 0.65(0.18,1.68) | 1.27(0.43,2.61) | 2.90(2.65,3.14) | 13.22(3.41,35.93) | 25.65(8.46,54.11) | 2.80(2.61,2.99) |
| Burundi | 1.22(0.33,3.22) | 1.47(0.49,3.29) | 0.54(0.29,0.80) | 25.52(6.70,66.09) | 28.53(9.48,66.19) | 0.23(-0.06,0.53) |
| Cabo Verde | 1.11(0.46,2.11) | 6.63(4.20,9.15) | 5.50(4.83,6.16) | 27.48(11.20,53.41) | 135.66(89.76,183.02) | 5.09(4.57,5.62) |
| Cambodia | 1.59(0.43,4.00) | 3.69(1.76,6.38) | 3.23(3.01,3.46) | 30.83(8.48,78.41) | 69.83(34.38,118.58) | 3.14(2.92,3.36) |
| Cameroon | 2.60(1.13,4.87) | 5.52(3.20,8.26) | 2.65(2.46,2.84) | 52.82(22.34,99.75) | 114.00(66.73,172.84) | 2.68(2.48,2.87) |
| Canada | 1.62(0.39,3.41) | 0.36(0.16,0.60) | -6.09(-6.49,-5.69) | 35.08(8.77,72.28) | 9.51(4.34,15.79) | -5.14(-5.45,-4.82) |
| Central African Republic | 1.63(0.40,4.13) | 2.59(0.78,5.94) | 1.73(1.49,1.98) | 33.24(8.22,84.70) | 51.69(15.04,119.35) | 1.61(1.36,1.87) |
| Chad | 0.95(0.23,2.60) | 1.79(0.65,3.89) | 2.40(2.22,2.59) | 19.32(4.66,54.66) | 36.68(12.76,83.39) | 2.42(2.23,2.61) |
| Chile | 5.76(2.75,9.61) | 3.55(2.74,4.39) | -1.46(-1.59,-1.32) | 109.63(53.23,181.84) | 66.98(52.28,82.90) | -1.55(-1.68,-1.43) |
| China | 6.80(3.26,11.50) | 13.87(11.25,16.59) | 2.85(2.46,3.24) | 141.06(67.56,238.25) | 294.05(239.00,352.73) | 2.87(2.56,3.19) |
| Colombia | 4.31(2.01,7.65) | 1.85(1.23,2.67) | -3.31(-3.47,-3.14) | 86.83(40.22,151.14) | 38.88(27.26,54.05) | -3.12(-3.31,-2.93) |
| Comoros | 0.89(0.29,1.99) | 2.02(0.97,3.51) | 2.70(2.60,2.81) | 18.98(6.52,42.61) | 41.33(19.98,70.86) | 2.52(2.40,2.64) |
| Congo | 3.75(1.32,8.18) | 8.94(4.75,14.59) | 3.01(2.75,3.27) | 77.68(27.94,165.55) | 177.19(95.22,282.72) | 2.82(2.56,3.08) |
| Cook Islands | 1.15(0.43,2.76) | 0.73(0.14,1.70) | -1.88(-2.20,-1.56) | 30.58(11.38,70.84) | 21.72(4.37,50.37) | -1.41(-1.70,-1.12) |
| Costa Rica | 2.09(0.96,3.74) | 1.63(1.09,2.30) | -1.26(-1.59,-0.93) | 38.60(18.10,67.81) | 32.61(23.03,43.40) | -0.87(-1.13,-0.60) |
| Croatia | 16.20(8.19,25.41) | 6.14(4.55,8.09) | -3.57(-3.87,-3.27) | 291.92(150.02,446.38) | 110.12(83.12,141.03) | -3.62(-3.89,-3.36) |
| Cuba | 4.80(1.86,9.03) | 3.75(1.82,6.38) | -0.68(-0.82,-0.55) | 97.05(39.14,180.11) | 74.04(35.91,123.22) | -0.70(-0.83,-0.57) |
| Cyprus | 7.59(3.11,13.68) | 2.45(1.77,3.34) | -4.44(-4.71,-4.17) | 106.33(44.65,190.11) | 37.05(27.28,48.84) | -4.22(-4.48,-3.96) |
| Czechia | 22.23(11.14,33.91) | 3.85(2.91,4.97) | -6.43(-6.71,-6.15) | 409.34(210.07,615.41) | 77.50(60.59,97.21) | -5.98(-6.14,-5.81) |
| Côte d'Ivoire | 2.31(0.83,5.08) | 3.82(1.87,6.46) | 1.66(1.35,1.97) | 48.52(16.85,109.60) | 80.57(39.51,138.27) | 1.58(1.25,1.92) |
| Democratic People's Republic of Korea | 4.88(1.98,9.26) | 9.26(5.64,13.39) | 2.40(2.29,2.52) | 112.94(46.22,215.95) | 220.01(134.33,321.54) | 2.51(2.39,2.63) |
| Democratic Republic of the Congo | 1.72(0.50,4.24) | 2.55(1.03,4.99) | 0.94(0.27,1.61) | 32.94(9.41,80.96) | 48.75(19.83,94.73) | 0.92(0.22,1.63) |
| Denmark | 4.87(2.00,8.47) | 1.05(0.64,1.54) | -5.46(-5.79,-5.13) | 92.07(38.05,158.24) | 20.20(12.37,28.87) | -5.38(-5.69,-5.06) |
| Djibouti | 2.53(0.81,5.46) | 9.31(4.59,14.84) | 5.21(4.73,5.70) | 53.83(17.07,118.86) | 190.93(95.68,301.75) | 5.05(4.57,5.54) |
| Dominica | 4.00(1.43,8.06) | 4.80(1.95,8.55) | 0.74(0.64,0.85) | 73.04(26.25,145.07) | 86.94(36.34,154.00) | 0.63(0.51,0.74) |
| Dominican Republic | 1.69(0.51,3.87) | 4.69(2.03,8.91) | 4.58(4.21,4.95) | 34.35(10.17,78.16) | 96.50(43.62,176.08) | 4.31(3.98,4.65) |
| Ecuador | 3.10(1.39,5.64) | 2.77(1.75,3.93) | 0.02(-0.33,0.38) | 64.07(28.90,116.10) | 55.25(35.68,77.35) | -0.22(-0.53,0.09) |
| Egypt | 13.91(9.90,18.80) | 19.86(13.82,28.12) | 1.70(1.38,2.02) | 316.20(235.55,421.17) | 487.24(354.95,655.57) | 1.95(1.66,2.25) |
| El Salvador | 1.68(0.64,3.17) | 2.01(1.21,3.15) | 0.49(0.15,0.84) | 32.91(12.40,63.06) | 41.32(25.53,63.19) | 0.70(0.39,1.02) |
| Equatorial Guinea | 1.46(0.37,4.01) | 9.73(5.42,15.16) | 7.81(7.34,8.30) | 30.20(7.73,82.72) | 190.58(110.21,289.77) | 7.65(7.17,8.13) |
| Eritrea | 1.23(0.30,3.15) | 3.85(1.63,7.23) | 3.70(3.39,4.02) | 27.18(7.42,68.51) | 76.51(33.36,140.75) | 3.29(2.99,3.59) |
| Estonia | 8.24(3.25,15.22) | 0.71(0.26,1.31) | -9.39(-10.23,-8.53) | 162.81(65.13,297.06) | 16.29(6.15,29.16) | -8.78(-9.54,-8.01) |
| Eswatini | 3.36(1.60,5.83) | 7.07(4.02,10.68) | 2.73(2.46,3.00) | 64.33(30.06,110.07) | 133.03(77.18,201.55) | 2.65(2.37,2.92) |
| Ethiopia | 0.47(0.13,1.19) | 1.41(0.71,2.42) | 4.14(3.84,4.45) | 9.59(2.65,24.19) | 26.71(13.68,46.19) | 3.85(3.52,4.19) |
| Fiji | 1.68(0.39,4.53) | 3.00(0.94,6.56) | 1.48(0.84,2.13) | 40.45(9.89,107.60) | 73.99(24.13,162.62) | 1.71(1.12,2.31) |
| Finland | 2.67(0.32,6.14) | 0.36(0.07,0.75) | -7.14(-7.60,-6.67) | 51.12(5.94,115.61) | 7.05(1.43,14.53) | -6.96(-7.43,-6.48) |
| France | 4.21(1.86,7.28) | 0.83(0.58,1.12) | -5.52(-5.68,-5.36) | 70.17(30.67,119.43) | 17.77(12.66,23.42) | -4.54(-4.72,-4.36) |
| Gabon | 4.17(1.79,7.93) | 10.18(5.91,15.02) | 3.03(2.58,3.48) | 86.14(36.54,160.81) | 204.48(124.09,297.30) | 2.92(2.47,3.37) |
| Gambia | 1.57(0.52,3.59) | 3.76(1.86,6.35) | 3.10(2.95,3.25) | 32.92(10.32,78.45) | 77.52(37.44,134.02) | 3.01(2.84,3.17) |
| Georgia | 9.13(4.62,16.14) | 7.76(5.31,10.55) | -0.13(-0.65,0.40) | 164.19(83.34,286.76) | 21.83(12.08,33.69) | 0.06(-0.39,0.51) |
| Germany | 7.58(3.52,12.51) | 1.35(0.96,1.79) | -6.00(-6.35,-5.66) | 134.39(62.56,218.34) | 27.92(20.31,36.58) | -5.33(-5.67,-4.99) |
| Ghana | 4.52(2.10,7.86) | 10.86(6.57,15.76) | 3.55(3.35,3.75) | 91.90(41.64,158.87) | 222.19(134.22,316.14) | 3.55(3.35,3.74) |
| Greece | 10.33(4.82,17.47) | 2.86(2.10,3.67) | -5.12(-5.62,-4.62) | 152.03(71.55,251.65) | 43.93(32.65,55.91) | -4.84(-5.23,-4.44) |
| Greenland | 2.82(0.66,7.62) | 0.94(0.09,2.67) | -4.89(-5.40,-4.38) | 57.22(13.04,154.06) | 21.03(2.00,59.45) | -4.37(-4.82,-3.92) |
| Grenada | 7.73(2.14,17.59) | 7.45(2.78,13.29) | -0.70(-0.94,-0.47) | 146.34(41.15,328.58) | 138.61(53.66,247.46) | -0.72(-0.96,-0.49) |
| Guam | 2.13(0.40,5.85) | 1.00(0.40,1.76) | -2.93(-3.85,-2.00) | 51.61(9.62,142.50) | 34.34(14.31,58.97) | -1.45(-2.20,-0.68) |
| Guatemala | 1.56(0.54,3.32) | 2.16(1.24,3.25) | 0.29(-0.17,0.76) | 27.29(9.36,59.49) | 37.86(21.52,56.80) | 0.38(-0.08,0.84) |
| Guinea | 1.14(0.35,2.80) | 2.20(0.95,4.28) | 2.71(2.58,2.84) | 22.59(6.56,57.39) | 44.77(19.02,88.92) | 2.78(2.64,2.91) |
| Guinea-Bissau | 1.60(0.49,4.00) | 3.12(1.41,5.63) | 2.47(2.36,2.58) | 34.66(10.15,87.31) | 66.16(29.32,123.22) | 2.40(2.29,2.51) |
| Guyana | 12.70(3.33,26.34) | 9.93(3.90,18.27) | -0.67(-0.85,-0.48) | 260.65(71.20,523.35) | 193.89(79.45,353.90) | -0.80(-0.98,-0.62) |
| Haiti | 1.85(0.47,4.80) | 3.32(1.14,7.40) | 2.32(2.19,2.45) | 34.46(8.72,91.02) | 60.57(20.84,136.49) | 2.27(2.13,2.41) |
| Honduras | 1.56(0.50,3.50) | 4.34(2.33,6.96) | 3.97(3.56,4.40) | 29.95(9.61,65.55) | 76.88(41.88,123.29) | 3.67(3.31,4.04) |
| Hungary | 15.92(8.37,24.49) | 4.88(3.73,6.26) | -4.56(-4.77,-4.34) | 344.51(187.12,528.20) | 110.11(85.56,138.88) | -4.38(-4.56,-4.20) |
| Iceland | 1.09(0.10,2.79) | 0.20(0.04,0.44) | -6.15(-6.56,-5.74) | 20.64(1.86,52.93) | 4.31(0.86,9.09) | -5.63(-5.91,-5.35) |
| India | 3.47(1.57,6.15) | 6.06(4.53,7.67) | 1.95(1.78,2.11) | 64.27(28.97,115.56) | 122.56(92.84,154.30) | 2.34(2.20,2.48) |
| Indonesia | 4.72(2.01,8.74) | 10.19(6.95,13.85) | 2.62(2.39,2.86) | 102.06(44.55,188.04) | 206.97(143.61,275.34) | 2.37(2.16,2.59) |
| Iran (Islamic Republic of) | 18.20(14.44,22.62) | 11.69(9.75,13.57) | -1.78(-2.07,-1.49) | 377.36(303.15,459.38) | 247.47(212.16,285.61) | -1.67(-1.91,-1.43) |
| Iraq | 21.39(14.05,28.97) | 26.00(19.64,32.51) | 0.97(0.75,1.19) | 466.37(307.27,626.72) | 542.49(413.38,680.04) | 0.77(0.54,0.99) |
| Ireland | 4.10(1.26,7.91) | 0.62(0.31,0.97) | -6.97(-7.19,-6.74) | 68.57(20.54,130.43) | 10.87(5.57,16.81) | -6.84(-7.07,-6.61) |
| Israel | 4.72(2.46,7.57) | 1.60(1.27,1.95) | -4.37(-4.60,-4.15) | 88.94(47.06,141.87) | 33.43(26.72,40.62) | -3.85(-4.06,-3.65) |
| Italy | 8.66(4.57,13.53) | 1.75(1.29,2.20) | -5.78(-5.93,-5.63) | 139.89(74.03,216.21) | 29.57(23.05,36.69) | -5.56(-5.73,-5.39) |
| Jamaica | 3.64(1.62,6.87) | 4.35(2.67,6.30) | 0.49(0.21,0.78) | 62.27(27.78,115.56) | 76.06(47.64,109.88) | 0.60(0.32,0.87) |
| Japan | 3.90(1.25,7.57) | 1.10(0.72,1.56) | -4.82(-5.27,-4.36) | 70.85(23.27,135.80) | 30.99(20.75,43.52) | -3.21(-3.62,-2.80) |
| Jordan | 19.74(14.78,25.22) | 10.98(8.34,13.89) | -2.42(-2.72,-2.11) | 415.17(319.11,518.15) | 235.96(185.53,292.50) | -2.31(-2.61,-2.01) |
| Kazakhstan | 12.15(5.83,21.54) | 13.08(8.58,18.29) | -0.22(-0.54,0.10) | 254.95(125.15,443.96) | 253.93(167.86,354.29) | -0.50(-0.84,-0.15) |
| Kenya | 1.03(0.44,1.98) | 2.52(1.40,4.01) | 3.71(3.42,4.00) | 21.49(9.72,41.52) | 50.92(29.18,80.12) | 3.60(3.30,3.91) |
| Kiribati | 1.51(0.38,4.13) | 2.21(0.65,5.60) | 0.39(0.04,0.73) | 37.79(9.17,100.07) | 53.53(15.79,129.94) | 0.26(-0.08,0.61) |
| Kuwait | 10.69(8.83,12.59) | 9.71(7.61,12.06) | 0.97(0.04,1.91) | 240.50(202.22,278.86) | 213.24(174.94,262.35) | 0.59(-0.21,1.39) |
| Kyrgyzstan | 9.34(4.05,16.94) | 9.36(5.72,13.64) | -0.47(-0.92,-0.02) | 195.61(84.38,354.23) | 202.41(125.19,294.74) | -0.40(-0.81,0.01) |
| Lao People's Democratic Republic | 1.86(0.53,4.36) | 4.23(2.06,6.85) | 2.77(2.52,3.03) | 38.61(11.25,89.81) | 85.00(42.36,139.30) | 2.70(2.46,2.94) |
| Latvia | 17.99(8.84,28.93) | 6.07(4.17,8.48) | -3.86(-4.34,-3.37) | 337.47(169.66,534.00) | 116.57(80.60,158.95) | -3.80(-4.32,-3.28) |
| Lebanon | 5.19(3.60,7.10) | 5.07(3.25,6.87) | 0.27(0.00,0.55) | 112.62(79.91,150.67) | 127.17(88.52,165.05) | 0.76(0.52,1.01) |
| Lesotho | 2.41(1.04,4.60) | 6.59(3.48,10.62) | 4.16(3.88,4.44) | 44.80(18.75,87.37) | 121.28(64.85,197.39) | 4.08(3.81,4.34) |
| Liberia | 1.53(0.53,3.41) | 2.41(1.12,4.31) | 2.64(2.17,3.11) | 30.86(10.55,71.11) | 48.57(22.11,88.91) | 2.66(2.15,3.17) |
| Libya | 8.41(5.10,13.05) | 12.41(8.02,18.34) | 1.40(1.05,1.75) | 183.46(114.59,273.81) | 299.01(201.04,418.07) | 1.72(1.35,2.09) |
| Lithuania | 8.36(3.71,14.10) | 3.48(2.20,5.01) | -2.88(-3.47,-2.28) | 185.13(82.89,310.53) | 73.20(47.32,104.55) | -3.04(-3.63,-2.45) |
| Luxembourg | 7.46(2.90,13.25) | 0.89(0.58,1.27) | -7.28(-7.57,-6.99) | 124.52(48.70,220.11) | 16.91(11.00,23.73) | -6.85(-7.12,-6.57) |
| Madagascar | 0.81(0.30,1.85) | 2.03(0.88,3.89) | 3.47(3.28,3.67) | 17.52(6.29,39.25) | 42.09(18.24,82.28) | 3.37(3.19,3.55) |
| Malawi | 0.83(0.25,2.12) | 1.69(0.71,3.22) | 3.12(2.89,3.36) | 15.93(4.79,41.16) | 33.14(13.44,62.25) | 3.18(2.95,3.41) |
| Malaysia | 8.83(3.99,14.06) | 4.31(2.87,6.16) | -2.21(-2.43,-2.00) | 209.67(96.40,328.61) | 104.99(73.81,143.90) | -2.03(-2.27,-1.79) |
| Maldives | 1.99(0.59,4.73) | 1.75(1.11,2.56) | -1.24(-1.56,-0.91) | 42.75(13.33,101.67) | 39.57(25.60,56.79) | -1.02(-1.33,-0.72) |
| Mali | 1.08(0.28,2.84) | 1.79(0.62,3.65) | 2.05(1.89,2.21) | 21.02(5.08,56.63) | 33.88(11.80,70.35) | 1.92(1.74,2.10) |
| Malta | 5.42(2.18,9.75) | 1.52(1.08,2.02) | -4.56(-4.90,-4.22) | 96.12(38.61,169.23) | 27.49(20.18,36.04) | -4.50(-4.78,-4.21) |
| Marshall Islands | 1.30(0.29,3.56) | 2.39(0.84,4.98) | 1.98(1.73,2.24) | 30.56(7.03,82.61) | 58.47(20.55,119.83) | 2.07(1.79,2.36) |
| Mauritania | 3.76(1.54,7.25) | 5.68(3.20,8.32) | 1.20(0.92,1.48) | 83.14(33.97,158.70) | 121.70(70.76,177.13) | 1.10(0.82,1.38) |
| Mauritius | 7.74(3.97,12.17) | 3.42(1.63,5.49) | -4.02(-4.63,-3.40) | 171.02(90.57,267.62) | 76.99(37.45,120.38) | -3.81(-4.36,-3.26) |
| Mexico | 4.51(2.25,7.44) | 2.20(1.60,2.83) | -2.91(-3.20,-2.63) | 88.49(45.38,143.82) | 46.90(34.66,59.29) | -2.63(-2.90,-2.35) |
| Micronesia (Federated States of) | 1.67(0.35,4.67) | 3.02(0.90,7.10) | 1.91(1.70,2.11) | 40.00(8.31,111.63) | 71.84(22.07,160.01) | 1.89(1.70,2.09) |
| Monaco | 2.95(0.31,6.49) | 1.58(0.89,2.41) | -1.83(-2.44,-1.21) | 48.75(5.20,107.60) | 30.06(17.70,43.94) | -1.32(-1.96,-0.67) |
| Mongolia | 1.41(0.61,2.73) | 5.04(3.29,7.08) | 4.78(4.30,5.26) | 40.20(17.81,78.59) | 125.68(84.04,175.82) | 4.23(3.88,4.59) |
| Montenegro | 5.20(2.80,7.96) | 5.54(4.16,7.19) | 1.00(0.61,1.39) | 108.88(60.41,161.46) | 106.07(80.77,133.17) | 0.47(0.16,0.78) |
| Morocco | 6.41(3.31,10.61) | 18.12(13.52,23.91) | 3.76(3.53,4.00) | 133.38(69.94,219.43) | 380.93(286.38,493.04) | 3.84(3.54,4.15) |
| Mozambique | 0.62(0.15,1.75) | 1.92(0.79,3.88) | 5.03(4.68,5.38) | 12.21(2.96,35.04) | 39.96(16.58,81.23) | 5.27(4.90,5.64) |
| Myanmar | 4.05(1.37,8.98) | 9.12(5.60,12.98) | 3.29(3.01,3.58) | 78.54(26.10,174.73) | 167.91(103.95,240.61) | 3.13(2.84,3.42) |
| Namibia | 4.51(2.18,7.94) | 8.01(4.64,11.90) | 2.01(1.75,2.27) | 86.46(41.47,151.02) | 146.72(86.34,216.31) | 1.83(1.57,2.09) |
| Nauru | 2.40(0.66,6.74) | 2.05(0.57,4.93) | -1.24(-1.48,-0.99) | 58.92(16.03,166.64) | 51.96(14.65,121.22) | -1.14(-1.39,-0.89) |
| Nepal | 1.86(0.50,4.80) | 5.70(3.30,8.94) | 4.40(4.06,4.73) | 34.13(8.86,86.55) | 102.70(59.59,161.73) | 4.35(4.02,4.69) |
| Netherlands | 5.16(2.34,8.69) | 1.36(0.97,1.79) | -5.13(-5.39,-4.86) | 90.85(41.86,151.52) | 26.14(18.95,34.15) | -4.75(-4.99,-4.51) |
| New Zealand | 1.25(0.08,3.27) | 0.35(0.06,0.71) | -5.06(-5.32,-4.79) | 22.25(1.55,58.98) | 6.56(1.15,13.20) | -4.77(-5.04,-4.51) |
| Nicaragua | 1.16(0.39,2.44) | 2.47(1.37,3.90) | 2.25(1.65,2.85) | 22.01(7.43,46.78) | 43.61(24.61,68.29) | 2.04(1.57,2.50) |
| Niger | 1.15(0.25,3.51) | 1.62(0.45,4.17) | 1.22(1.08,1.37) | 22.87(4.90,70.66) | 30.72(8.20,79.57) | 1.00(0.82,1.17) |
| Nigeria | 2.66(1.20,5.26) | 5.60(3.53,8.14) | 2.60(2.30,2.89) | 50.37(22.48,99.19) | 110.06(69.61,159.79) | 2.78(2.48,3.07) |
| Niue | 1.70(0.64,3.79) | 1.42(0.31,3.23) | -1.23(-1.48,-0.98) | 41.18(16.02,89.85) | 35.94(8.12,81.91) | -1.06(-1.31,-0.82) |
| North Macedonia | 36.06(21.11,51.78) | 33.35(25.71,41.80) | -0.36(-0.73,0.02) | 622.03(368.69,884.17) | 528.84(412.13,661.39) | -0.73(-1.06,-0.41) |
| Northern Mariana Islands | 3.03(0.97,7.34) | 1.49(0.77,2.42) | -2.85(-3.55,-2.15) | 72.39(23.68,175.21) | 39.23(20.43,62.14) | -2.47(-3.09,-1.85) |
| Norway | 3.43(0.94,6.91) | 0.38(0.14,0.68) | -7.94(-8.26,-7.62) | 61.03(16.43,121.06) | 8.70(3.28,15.49) | -6.89(-7.15,-6.62) |
| Oman | 12.29(6.58,19.34) | 18.17(13.42,23.60) | 1.56(1.22,1.91) | 261.85(140.16,407.52) | 346.72(262.40,442.05) | 1.06(0.62,1.49) |
| Pakistan | 2.78(0.92,6.18) | 8.47(5.60,11.99) | 4.20(4.01,4.40) | 52.27(17.51,118.69) | 168.19(109.47,235.92) | 4.40(4.19,4.60) |
| Palau | 1.70(0.02,5.50) | 1.56(0.01,3.97) | 0.54(-0.30,1.39) | 44.53(0.50,141.37) | 42.27(0.17,104.06) | 0.67(-0.16,1.50) |
| Palestine | 11.45(5.52,18.60) | 18.03(13.00,23.12) | 1.62(1.28,1.96) | 220.00(106.55,361.79) | 344.65(251.05,434.38) | 1.58(1.27,1.88) |
| Panama | 2.70(1.26,5.03) | 1.68(0.96,2.55) | -1.51(-1.83,-1.20) | 48.76(22.90,88.59) | 31.06(18.28,45.32) | -1.34(-1.60,-1.09) |
| Papua New Guinea | 0.54(0.09,1.71) | 0.93(0.21,2.51) | 1.80(1.60,2.01) | 13.28(2.27,42.38) | 22.56(5.34,59.58) | 1.80(1.59,2.00) |
| Paraguay | 2.43(1.04,4.48) | 2.47(1.50,3.80) | -0.07(-0.30,0.16) | 43.92(19.10,81.30) | 46.78(29.03,71.25) | 0.00(-0.24,0.24) |
| Peru | 3.23(1.49,5.50) | 2.17(1.37,3.22) | -1.44(-1.89,-0.99) | 61.09(28.47,104.83) | 45.54(31.07,64.28) | -1.13(-1.58,-0.68) |
| Philippines | 2.80(1.32,4.77) | 3.97(2.77,5.35) | 1.37(0.66,2.09) | 59.42(28.91,100.09) | 94.51(66.74,125.73) | 1.68(1.06,2.31) |
| Poland | 12.93(7.33,19.22) | 5.91(4.77,7.21) | -3.29(-3.63,-2.95) | 265.25(150.90,387.05) | 121.90(98.41,148.65) | -3.13(-3.38,-2.88) |
| Portugal | 8.97(2.88,17.34) | 1.26(0.72,1.86) | -7.38(-7.76,-6.99) | 142.14(47.34,273.33) | 20.89(12.07,30.56) | -7.15(-7.50,-6.81) |
| Puerto Rico | 0.83(0.01,2.84) | 0.29(0.06,0.56) | -4.66(-5.25,-4.06) | 15.36(0.12,53.10) | 6.90(1.50,12.75) | -3.51(-4.05,-2.96) |
| Qatar | 15.33(11.13,20.50) | 10.24(7.85,13.69) | -1.26(-1.54,-0.97) | 307.95(242.37,400.20) | 187.73(150.13,237.48) | -1.74(-2.00,-1.49) |
| Republic of Korea | 15.80(8.02,25.14) | 4.25(3.27,5.45) | -5.37(-5.74,-4.99) | 314.74(161.14,487.15) | 91.31(72.54,116.04) | -4.99(-5.31,-4.68) |
| Republic of Moldova | 8.69(4.06,14.88) | 5.61(3.18,8.33) | -0.69(-1.39,0.01) | 162.16(75.70,273.25) | 120.07(68.35,179.13) | -0.18(-0.91,0.56) |
| Romania | 15.21(7.93,24.23) | 8.39(6.32,10.75) | -2.66(-3.05,-2.28) | 283.58(149.73,447.21) | 156.52(119.14,199.25) | -2.67(-3.04,-2.29) |
| Russian Federation | 19.75(8.47,33.49) | 7.34(4.12,10.73) | -3.84(-4.46,-3.21) | 376.33(162.63,631.59) | 143.72(80.58,210.60) | -3.72(-4.35,-3.09) |
| Rwanda | 1.60(0.49,3.67) | 2.55(1.04,4.90) | 1.41(1.10,1.73) | 32.93(10.01,77.52) | 48.15(20.17,93.15) | 1.02(0.67,1.37) |
| Saint Kitts and Nevis | 6.49(2.29,13.01) | 3.38(1.52,5.52) | -2.37(-2.78,-1.96) | 118.94(43.86,237.36) | 60.80(27.47,100.57) | -2.66(-3.07,-2.25) |
| Saint Lucia | 7.94(2.66,16.69) | 6.11(2.57,10.69) | -1.75(-2.20,-1.31) | 140.74(47.09,293.28) | 109.94(46.82,189.67) | -1.53(-1.91,-1.14) |
| Saint Vincent and the Grenadines | 7.12(2.08,16.02) | 6.12(2.28,10.92) | -0.41(-0.80,-0.02) | 134.81(39.79,292.89) | 112.66(42.79,197.78) | -0.58(-0.93,-0.22) |
| Samoa | 2.05(0.47,5.62) | 2.22(0.61,4.96) | -0.02(-0.14,0.09) | 48.05(11.50,128.54) | 53.89(14.87,120.94) | 0.09(-0.03,0.21) |
| San Marino | 2.62(0.54,5.51) | 1.16(0.44,2.09) | -3.10(-3.48,-2.71) | 45.14(9.85,94.34) | 21.23(8.52,36.73) | -2.90(-3.30,-2.51) |
| Sao Tome and Principe | 1.64(0.66,3.41) | 5.08(2.52,8.62) | 4.44(4.20,4.69) | 35.05(14.04,72.56) | 110.68(55.22,181.95) | 4.44(4.21,4.68) |
| Saudi Arabia | 13.07(7.19,20.41) | 20.11(15.21,24.83) | 0.92(0.29,1.55) | 259.97(143.81,416.43) | 437.68(339.65,537.80) | 1.31(0.68,1.95) |
| Senegal | 2.09(0.76,4.59) | 3.39(1.62,5.83) | 1.38(1.17,1.60) | 44.52(15.26,97.72) | 69.83(33.64,120.70) | 1.26(1.06,1.47) |
| Serbia | 26.32(14.75,39.69) | 19.51(15.28,24.29) | -1.11(-1.53,-0.69) | 475.49(268.71,709.82) | 328.08(255.98,410.48) | -1.48(-1.89,-1.06) |
| Seychelles | 5.03(2.64,7.97) | 3.81(1.90,6.00) | -1.08(-1.25,-0.91) | 118.76(62.41,187.08) | 92.65(48.64,141.14) | -1.09(-1.23,-0.95) |
| Sierra Leone | 1.61(0.50,3.84) | 2.61(1.08,5.09) | 2.08(1.80,2.37) | 33.65(10.59,82.11) | 54.84(22.77,106.88) | 2.09(1.77,2.41) |
| Singapore | 7.59(2.52,13.90) | 1.37(0.86,1.94) | -6.25(-6.61,-5.89) | 169.13(56.43,313.57) | 38.92(24.77,54.47) | -5.45(-5.87,-5.02) |
| Slovakia | 13.01(6.54,19.90) | 5.59(4.12,7.26) | -2.95(-3.16,-2.73) | 280.34(147.84,421.75) | 121.80(93.29,155.41) | -2.94(-3.15,-2.72) |
| Slovenia | 13.77(7.04,23.24) | 2.72(1.92,3.79) | -5.87(-6.15,-5.59) | 258.94(135.84,425.06) | 53.84(39.72,70.15) | -5.69(-5.99,-5.40) |
| Solomon Islands | 0.73(0.14,2.25) | 2.01(0.55,5.15) | 3.30(3.12,3.48) | 16.76(3.12,51.55) | 43.59(11.82,113.85) | 3.11(2.92,3.30) |
| Somalia | 0.43(0.09,1.28) | 0.60(0.14,1.69) | 1.78(1.49,2.07) | 8.74(1.69,26.65) | 11.62(2.46,33.88) | 1.48(1.20,1.76) |
| South Africa | 5.02(3.61,6.62) | 7.28(5.59,9.02) | 1.53(1.01,2.05) | 111.95(82.13,144.96) | 147.56(114.34,182.05) | 1.19(0.74,1.64) |
| South Sudan | 1.30(0.41,3.04) | 2.15(0.80,4.07) | 2.16(2.02,2.31) | 27.30(8.65,63.79) | 44.12(17.25,84.83) | 2.03(1.89,2.18) |
| Spain | 4.76(1.89,8.60) | 0.75(0.49,1.05) | -6.50(-6.73,-6.27) | 78.48(30.35,142.76) | 14.58(9.70,20.40) | -5.81(-6.00,-5.62) |
| Sri Lanka | 5.01(2.12,9.05) | 4.76(2.90,7.02) | 0.39(-0.25,1.04) | 91.69(37.97,165.50) | 97.06(61.75,139.87) | 0.54(-0.02,1.10) |
| Sudan | 3.62(1.23,8.37) | 16.60(9.31,27.53) | 5.94(5.70,6.17) | 71.01(23.90,160.33) | 354.91(206.02,573.01) | 6.32(6.06,6.58) |
| Suriname | 6.10(2.07,11.63) | 6.03(2.74,10.39) | -0.28(-0.84,0.29) | 118.55(41.59,227.02) | 119.68(56.77,203.30) | -0.23(-0.75,0.28) |
| Sweden | 2.07(0.37,4.47) | 0.31(0.08,0.62) | -6.72(-7.21,-6.23) | 39.83(7.15,86.66) | 6.87(1.77,13.74) | -6.16(-6.57,-5.74) |
| Switzerland | 4.30(1.80,7.48) | 0.66(0.42,0.95) | -6.28(-6.50,-6.05) | 72.59(31.23,124.32) | 12.96(8.50,18.03) | -5.73(-5.98,-5.48) |
| Syrian Arab Republic | 12.94(9.03,17.88) | 11.12(7.82,14.94) | -1.09(-1.39,-0.78) | 287.48(200.08,385.97) | 240.75(177.48,320.08) | -1.09(-1.35,-0.83) |
| Taiwan (Province of China) | 6.73(3.52,10.78) | 2.32(1.76,3.04) | -4.00(-4.31,-3.68) | 147.86(80.32,236.80) | 69.70(54.80,87.12) | -2.80(-3.07,-2.54) |
| Tajikistan | 1.67(0.56,3.51) | 6.47(3.41,10.34) | 5.10(4.67,5.53) | 36.62(12.63,75.48) | 121.88(65.37,190.91) | 4.58(4.29,4.88) |
| Thailand | 4.06(1.92,6.55) | 3.37(2.37,4.66) | -1.19(-1.51,-0.86) | 98.87(47.24,159.28) | 92.23(68.12,120.78) | -0.73(-1.02,-0.43) |
| Timor-Leste | 0.87(0.21,2.43) | 3.71(1.57,7.10) | 6.46(5.78,7.14) | 17.81(4.25,48.73) | 75.00(30.64,140.62) | 6.44(5.74,7.15) |
| Togo | 2.02(0.77,4.09) | 3.40(1.68,5.72) | 1.70(1.41,1.99) | 42.90(15.77,88.71) | 72.90(35.84,121.57) | 1.70(1.39,2.00) |
| Tokelau | 1.47(0.04,5.45) | 1.09(0.01,3.09) | -1.57(-1.88,-1.25) | 35.98(1.10,132.09) | 27.39(0.15,75.55) | -1.45(-1.77,-1.13) |
| Tonga | 1.12(0.29,2.81) | 1.76(0.56,3.87) | 1.39(1.11,1.67) | 27.81(7.10,69.68) | 44.03(14.61,95.02) | 1.37(1.13,1.61) |
| Trinidad and Tobago | 10.29(1.86,22.17) | 5.66(1.82,10.51) | -2.63(-3.01,-2.24) | 196.94(38.97,412.24) | 110.86(37.23,204.89) | -2.59(-2.95,-2.22) |
| Tunisia | 9.40(6.08,13.44) | 11.66(7.71,16.60) | 0.76(0.30,1.23) | 177.15(115.19,249.57) | 238.08(163.85,333.95) | 1.04(0.57,1.51) |
| Turkey | 5.28(3.66,7.33) | 6.03(4.41,7.86) | 1.44(0.90,1.99) | 114.63(82.14,154.32) | 124.35(94.76,156.39) | 0.96(0.56,1.36) |
| Turkmenistan | 11.36(4.30,19.93) | 14.88(8.65,22.87) | 0.99(0.63,1.34) | 256.75(99.11,447.75) | 345.40(207.09,519.14) | 1.15(0.83,1.47) |
| Tuvalu | 0.91(0.22,2.62) | 1.52(0.59,3.20) | 1.27(0.95,1.60) | 21.90(5.37,61.92) | 37.11(14.86,78.10) | 1.38(1.06,1.69) |
| Uganda | 0.81(0.24,1.95) | 2.36(1.11,4.24) | 4.28(4.03,4.53) | 16.26(4.82,40.88) | 46.92(22.01,84.45) | 4.22(3.97,4.47) |
| Ukraine | 19.07(8.86,31.42) | 8.76(5.31,12.75) | -3.34(-3.65,-3.03) | 364.38(172.50,589.52) | 185.90(115.42,268.14) | -2.90(-3.20,-2.61) |
| United Arab Emirates | 29.34(21.22,43.57) | 17.69(12.45,24.31) | -1.86(-2.51,-1.21) | 593.09(430.94,845.38) | 410.78(301.15,545.76) | -1.35(-1.84,-0.87) |
| United Kingdom | 5.73(2.32,10.07) | 0.95(0.61,1.34) | -6.80(-7.06,-6.53) | 96.94(40.27,167.82) | 17.19(11.21,23.96) | -6.46(-6.68,-6.24) |
| United Republic of Tanzania | 0.76(0.26,1.72) | 2.46(1.14,4.39) | 4.86(4.57,5.15) | 15.60(5.46,35.77) | 48.82(22.89,88.82) | 4.59(4.37,4.82) |
| United States of America | 2.25(0.85,4.23) | 0.51(0.27,0.79) | -5.75(-6.09,-5.41) | 51.85(19.84,96.38) | 14.84(7.78,23.14) | -4.57(-4.81,-4.33) |
| United States Virgin Islands | 1.75(0.29,4.77) | 1.39(0.65,2.19) | -0.86(-1.41,-0.31) | 33.77(5.82,89.91) | 27.18(13.08,42.88) | -0.84(-1.36,-0.32) |
| Uruguay | 4.16(1.42,8.60) | 1.74(0.86,2.83) | -3.20(-3.36,-3.04) | 84.10(28.77,172.74) | 34.39(17.52,55.00) | -3.23(-3.41,-3.04) |
| Uzbekistan | 9.64(3.99,17.25) | 18.95(11.75,26.91) | 1.57(0.76,2.39) | 229.00(95.63,401.67) | 391.26(245.17,556.24) | 1.09(0.45,1.74) |
| Vanuatu | 1.41(0.30,3.84) | 2.50(0.68,5.91) | 1.52(1.29,1.75) | 32.84(7.31,89.34) | 59.16(15.89,134.74) | 1.58(1.35,1.82) |
| Venezuela (Bolivarian Republic of) | 3.94(1.58,6.89) | 3.37(2.12,5.06) | -0.58(-0.78,-0.39) | 76.35(31.44,131.47) | 63.37(40.86,91.88) | -0.69(-0.87,-0.50) |
| Viet Nam | 3.59(1.29,7.33) | 9.16(6.05,12.30) | 4.04(3.37,4.71) | 68.67(25.06,140.40) | 179.14(121.30,238.62) | 4.06(3.45,4.68) |
| Yemen | 3.08(0.82,7.95) | 14.16(6.65,23.54) | 6.14(5.71,6.57) | 61.12(16.13,154.96) | 298.90(143.89,478.76) | 6.41(5.96,6.86) |
| Zambia | 1.29(0.46,2.71) | 4.85(2.44,7.97) | 5.10(4.71,5.50) | 25.23(8.90,53.26) | 91.54(45.96,151.20) | 4.90(4.54,5.27) |
| Zimbabwe | 2.48(1.13,4.29) | 3.64(1.78,6.16) | 1.49(1.18,1.80) | 46.70(21.49,82.91) | 71.82(35.12,120.94) | 1.60(1.26,1.94) |

**Supplementary Table 4 Death Cases, DALYs and Increased Proportion of Ischaemic Stroke Attributed to Ambient Particulate Matter Pollution in Countries of the World from 1990 to 2019**

| **Country** | **Death Cases** | | | **DALYs** | | |
| --- | --- | --- | --- | --- | --- | --- |
|  | **1990** | **2019** | **Increased Proportion** | **1990** | **2019** | **Increased Proportion** |
| Afghanistan | 143.48 | 807.92 | 5.63 | 3390.32 | 22815.22 | 6.73 |
| Albania | 57.57 | 142.69 | 2.48 | 1386.90 | 3023.95 | 2.18 |
| Algeria | 1251.34 | 3844.88 | 3.07 | 30479.75 | 90582.85 | 2.97 |
| American Samoa | 0.26 | 0.47 | 1.81 | 8.53 | 14.21 | 1.67 |
| Andorra | 0.87 | 0.94 | 1.08 | 20.05 | 17.63 | 0.88 |
| Angola | 30.98 | 370.18 | 11.95 | 855.29 | 10024.70 | 11.72 |
| Antigua and Barbuda | 3.09 | 3.43 | 1.11 | 54.75 | 68.13 | 1.24 |
| Argentina | 1192.35 | 1002.91 | 0.84 | 24189.42 | 19710.33 | 0.81 |
| Armenia | 274.57 | 351.95 | 1.28 | 6332.46 | 7254.66 | 1.15 |
| Australia | 264.81 | 176.63 | 0.67 | 4951.66 | 3074.32 | 0.62 |
| Austria | 1042.94 | 218.47 | 0.21 | 17094.21 | 3977.60 | 0.23 |
| Azerbaijan | 185.25 | 624.05 | 3.37 | 4878.31 | 15338.43 | 3.14 |
| Bahrain | 17.14 | 44.11 | 2.57 | 520.36 | 1585.78 | 3.05 |
| Bangladesh | 982.82 | 9320.48 | 9.48 | 20180.42 | 178405.75 | 8.84 |
| Barbados | 26.93 | 25.78 | 0.96 | 453.23 | 457.96 | 1.01 |
| Belarus | 1829.67 | 1341.77 | 0.73 | 39894.56 | 27184.38 | 0.68 |
| Belgium | 1033.46 | 369.38 | 0.36 | 17466.02 | 5942.99 | 0.34 |
| Belize | 2.13 | 7.19 | 3.37 | 41.72 | 165.44 | 3.97 |
| Benin | 26.01 | 103.97 | 4.00 | 591.39 | 2586.33 | 4.37 |
| Bermuda | 1.49 | 0.85 | 0.57 | 30.13 | 15.66 | 0.52 |
| Bhutan | 1.69 | 19.13 | 11.31 | 41.12 | 401.78 | 9.77 |
| Bolivia (Plurinational State of) | 119.96 | 297.81 | 2.48 | 2691.10 | 6252.99 | 2.32 |
| Bosnia and Herzegovina | 447.54 | 1014.71 | 2.27 | 10747.86 | 19504.78 | 1.81 |
| Botswana | 18.76 | 91.18 | 4.86 | 447.59 | 2217.05 | 4.95 |
| Brazil | 3821.28 | 5037.15 | 1.32 | 89721.88 | 104068.28 | 1.16 |
| Brunei Darussalam | 2.23 | 2.36 | 1.06 | 64.58 | 77.62 | 1.20 |
| Bulgaria | 2239.70 | 2441.45 | 1.09 | 47197.05 | 43988.20 | 0.93 |
| Burkina Faso | 19.59 | 83.36 | 4.26 | 532.76 | 2226.57 | 4.18 |
| Burundi | 23.22 | 44.45 | 1.91 | 570.87 | 1136.32 | 1.99 |
| Cambodia | 49.87 | 328.56 | 6.59 | 1212.20 | 7436.33 | 6.13 |
| Cameroon | 83.70 | 477.08 | 5.70 | 2201.12 | 13108.10 | 5.96 |
| Canada | 513.95 | 283.53 | 0.55 | 11418.18 | 6383.92 | 0.56 |
| Cabo Verde | 2.70 | 27.39 | 10.15 | 64.49 | 582.62 | 9.03 |
| Central African Republic | 12.80 | 35.56 | 2.78 | 341.73 | 960.24 | 2.81 |
| Chad | 21.98 | 75.16 | 3.42 | 524.09 | 1968.92 | 3.76 |
| Chile | 505.47 | 850.89 | 1.68 | 10575.41 | 15994.94 | 1.51 |
| China | 44633.59 | 245378.84 | 5.50 | 1124039.81 | 5737751.02 | 5.10 |
| Colombia | 629.95 | 1005.80 | 1.60 | 14761.06 | 20498.97 | 1.39 |
| Comoros | 1.65 | 8.37 | 5.08 | 40.40 | 192.33 | 4.76 |
| Congo | 29.22 | 160.32 | 5.49 | 762.37 | 4107.66 | 5.39 |
| Costa Rica | 33.14 | 82.26 | 2.48 | 670.24 | 1647.14 | 2.46 |
| Côte d'Ivoire | 60.90 | 280.64 | 4.61 | 1880.25 | 8296.75 | 4.41 |
| Croatia | 936.58 | 610.59 | 0.65 | 17934.21 | 10259.79 | 0.57 |
| Cuba | 481.85 | 751.71 | 1.56 | 10033.48 | 14010.31 | 1.40 |
| Cyprus | 44.81 | 41.90 | 0.94 | 750.48 | 690.10 | 0.92 |
| Czechia | 3024.99 | 868.91 | 0.29 | 57018.69 | 16387.43 | 0.29 |
| Democratic Republic of the Congo | 172.59 | 622.66 | 3.61 | 4441.41 | 15119.39 | 3.40 |
| Denmark | 435.01 | 137.07 | 0.32 | 7752.48 | 2347.95 | 0.30 |
| Djibouti | 2.17 | 36.25 | 16.68 | 66.95 | 1020.77 | 15.25 |
| Dominica | 3.12 | 4.44 | 1.43 | 54.96 | 78.66 | 1.43 |
| Dominican Republic | 52.85 | 405.02 | 7.66 | 1282.21 | 8910.96 | 6.95 |
| Ecuador | 144.82 | 370.50 | 2.56 | 3447.23 | 8198.11 | 2.38 |
| Egypt | 3236.89 | 10523.99 | 3.25 | 90776.84 | 317076.57 | 3.49 |
| El Salvador | 46.02 | 126.91 | 2.76 | 960.78 | 2491.50 | 2.59 |
| Equatorial Guinea | 2.11 | 32.92 | 15.61 | 53.94 | 824.98 | 15.29 |
| Eritrea | 7.63 | 64.47 | 8.45 | 239.73 | 1769.97 | 7.38 |
| Estonia | 167.36 | 21.66 | 0.13 | 3357.60 | 423.04 | 0.13 |
| Ethiopia | 60.49 | 428.52 | 7.08 | 1688.17 | 10194.04 | 6.04 |
| Micronesia (Federated States of) | 0.61 | 1.54 | 2.52 | 18.75 | 48.68 | 2.60 |
| Fiji | 4.51 | 17.06 | 3.78 | 144.71 | 532.25 | 3.68 |
| Finland | 195.42 | 53.18 | 0.27 | 3689.38 | 876.98 | 0.24 |
| France | 3889.19 | 1524.10 | 0.39 | 61425.85 | 24948.78 | 0.41 |
| Gabon | 18.92 | 80.66 | 4.26 | 453.39 | 1940.63 | 4.28 |
| Georgia | 487.78 | 508.06 | 1.04 | 9633.12 | 3341.50 | 0.35 |
| Germany | 10343.30 | 3138.94 | 0.30 | 176935.65 | 54430.36 | 0.31 |
| Ghana | 203.51 | 1323.68 | 6.50 | 5486.15 | 34507.98 | 6.29 |
| Greece | 1538.40 | 956.46 | 0.62 | 23549.68 | 12458.09 | 0.53 |
| Greenland | 0.63 | 0.48 | 0.77 | 17.07 | 13.00 | 0.76 |
| Grenada | 6.29 | 7.04 | 1.12 | 109.54 | 146.61 | 1.34 |
| Guam | 1.09 | 1.81 | 1.65 | 38.01 | 63.64 | 1.67 |
| Guatemala | 37.33 | 203.74 | 5.46 | 860.72 | 4128.59 | 4.80 |
| Guinea | 30.21 | 99.30 | 3.29 | 712.90 | 2396.30 | 3.36 |
| Guinea-Bissau | 4.87 | 16.20 | 3.32 | 136.06 | 471.24 | 3.46 |
| Guyana | 41.82 | 50.74 | 1.21 | 973.05 | 1144.30 | 1.18 |
| Haiti | 44.66 | 173.17 | 3.88 | 1030.22 | 3887.15 | 3.77 |
| Honduras | 27.23 | 218.51 | 8.02 | 607.21 | 4414.92 | 7.27 |
| Hungary | 2274.93 | 1014.28 | 0.45 | 50362.82 | 20998.18 | 0.42 |
| Iceland | 3.33 | 1.34 | 0.40 | 60.56 | 24.31 | 0.40 |
| India | 10131.09 | 58035.63 | 5.73 | 251629.10 | 1351184.58 | 5.37 |
| Indonesia | 3358.97 | 16210.50 | 4.83 | 93066.97 | 408783.88 | 4.39 |
| Iran (Islamic Republic of) | 3431.93 | 7448.37 | 2.17 | 93504.66 | 178675.10 | 1.91 |
| Iraq | 1454.47 | 4700.02 | 3.23 | 35577.73 | 118984.56 | 3.34 |
| Ireland | 165.93 | 48.93 | 0.29 | 2889.64 | 818.34 | 0.28 |
| Israel | 222.17 | 206.87 | 0.93 | 4378.34 | 3917.83 | 0.89 |
| Italy | 7852.60 | 3441.45 | 0.44 | 128062.48 | 47577.76 | 0.37 |
| Jamaica | 68.73 | 135.96 | 1.98 | 1163.01 | 2261.49 | 1.94 |
| Japan | 5970.00 | 5628.78 | 0.94 | 116460.64 | 109327.30 | 0.94 |
| Jordan | 191.08 | 514.72 | 2.69 | 5110.62 | 14387.85 | 2.82 |
| Kazakhstan | 1344.76 | 1850.89 | 1.38 | 31343.74 | 41545.21 | 1.33 |
| Kenya | 63.72 | 382.88 | 6.01 | 1676.58 | 10218.43 | 6.09 |
| Kiribati | 0.46 | 1.16 | 2.55 | 14.46 | 37.53 | 2.60 |
| Kuwait | 46.70 | 186.50 | 3.99 | 1580.44 | 5707.36 | 3.61 |
| Kyrgyzstan | 266.53 | 365.10 | 1.37 | 5816.91 | 9048.35 | 1.56 |
| Lao People's Democratic Republic | 28.50 | 137.81 | 4.84 | 734.49 | 3370.88 | 4.59 |
| Latvia | 643.76 | 279.83 | 0.43 | 12204.33 | 4787.91 | 0.39 |
| Lebanon | 89.00 | 260.52 | 2.93 | 2363.07 | 6702.57 | 2.84 |
| Lesotho | 18.62 | 60.02 | 3.22 | 402.36 | 1361.84 | 3.38 |
| Liberia | 13.32 | 36.15 | 2.71 | 325.12 | 961.43 | 2.96 |
| Libya | 133.27 | 559.27 | 4.20 | 3347.26 | 15871.34 | 4.74 |
| Lithuania | 378.82 | 230.17 | 0.61 | 8366.83 | 4221.43 | 0.50 |
| Luxembourg | 41.36 | 10.32 | 0.25 | 698.35 | 173.80 | 0.25 |
| North Macedonia | 544.91 | 885.85 | 1.63 | 10516.39 | 15901.89 | 1.51 |
| Madagascar | 32.77 | 148.05 | 4.52 | 861.77 | 4148.28 | 4.81 |
| Malawi | 21.90 | 92.06 | 4.20 | 542.28 | 2219.29 | 4.09 |
| Malaysia | 681.85 | 962.62 | 1.41 | 18808.59 | 27544.30 | 1.46 |
| Maldives | 1.14 | 4.25 | 3.74 | 34.44 | 123.19 | 3.58 |
| Mali | 30.86 | 110.26 | 3.57 | 788.93 | 2682.18 | 3.40 |
| Malta | 21.95 | 15.84 | 0.72 | 408.53 | 261.59 | 0.64 |
| Marshall Islands | 0.17 | 0.57 | 3.41 | 5.03 | 20.06 | 3.99 |
| Mauritania | 31.15 | 97.00 | 3.11 | 819.78 | 2475.80 | 3.02 |
| Mauritius | 48.10 | 53.00 | 1.10 | 1230.07 | 1282.36 | 1.04 |
| Mexico | 1588.11 | 2338.51 | 1.47 | 36953.67 | 53892.61 | 1.46 |
| Republic of Moldova | 316.09 | 327.07 | 1.03 | 6715.87 | 6948.88 | 1.03 |
| Mongolia | 13.43 | 94.37 | 7.02 | 437.88 | 3112.47 | 7.11 |
| Montenegro | 28.84 | 51.72 | 1.79 | 652.37 | 1033.47 | 1.58 |
| Morocco | 701.95 | 4687.79 | 6.68 | 17446.56 | 114694.01 | 6.57 |
| Mozambique | 26.95 | 161.97 | 6.01 | 655.53 | 4080.39 | 6.22 |
| Myanmar | 715.64 | 3364.56 | 4.70 | 16872.36 | 71142.16 | 4.22 |
| Namibia | 26.35 | 91.29 | 3.46 | 592.57 | 1893.69 | 3.20 |
| Nepal | 122.71 | 1025.78 | 8.36 | 2810.32 | 21377.31 | 7.61 |
| Netherlands | 1076.10 | 518.93 | 0.48 | 18672.84 | 8929.96 | 0.48 |
| New Zealand | 48.58 | 30.96 | 0.64 | 884.00 | 511.76 | 0.58 |
| Nicaragua | 14.58 | 86.09 | 5.90 | 317.44 | 1771.71 | 5.58 |
| Niger | 21.26 | 82.10 | 3.86 | 586.15 | 2148.05 | 3.66 |
| Nigeria | 879.61 | 3504.73 | 3.98 | 20200.24 | 87822.57 | 4.35 |
| Democratic People's Republic of Korea | 619.97 | 2803.55 | 4.52 | 17197.23 | 70330.36 | 4.09 |
| Northern Mariana Islands | 0.34 | 0.54 | 1.57 | 13.82 | 18.73 | 1.36 |
| Norway | 266.28 | 43.04 | 0.16 | 4440.32 | 840.74 | 0.19 |
| Oman | 59.67 | 180.71 | 3.03 | 1676.12 | 5467.14 | 3.26 |
| Pakistan | 1334.66 | 6990.95 | 5.24 | 28226.53 | 172686.06 | 6.12 |
| Palestine | 83.78 | 311.31 | 3.72 | 1804.55 | 7353.54 | 4.07 |
| Panama | 36.54 | 70.75 | 1.94 | 708.27 | 1282.82 | 1.81 |
| Papua New Guinea | 6.69 | 29.83 | 4.46 | 231.33 | 1033.27 | 4.47 |
| Paraguay | 47.56 | 124.19 | 2.61 | 927.19 | 2490.94 | 2.69 |
| Peru | 334.05 | 701.39 | 2.10 | 7058.10 | 14694.38 | 2.08 |
| Philippines | 536.15 | 2503.52 | 4.67 | 16033.23 | 71795.18 | 4.48 |
| Poland | 5353.47 | 4433.93 | 0.83 | 114765.54 | 85397.39 | 0.74 |
| Portugal | 1185.35 | 386.56 | 0.33 | 19893.84 | 5532.13 | 0.28 |
| Puerto Rico | 28.62 | 25.22 | 0.88 | 556.42 | 479.21 | 0.86 |
| Qatar | 8.74 | 27.04 | 3.09 | 370.82 | 1714.69 | 4.62 |
| Romania | 3698.24 | 3455.44 | 0.93 | 76167.47 | 60412.34 | 0.79 |
| Russian Federation | 32279.48 | 17524.18 | 0.54 | 664783.07 | 337997.65 | 0.51 |
| Rwanda | 34.20 | 103.66 | 3.03 | 875.08 | 2507.31 | 2.87 |
| Saint Lucia | 6.26 | 12.35 | 1.97 | 121.15 | 230.92 | 1.91 |
| Saint Vincent and the Grenadines | 4.94 | 7.60 | 1.54 | 97.85 | 147.71 | 1.51 |
| Samoa | 1.49 | 2.79 | 1.87 | 40.71 | 76.55 | 1.88 |
| Sao Tome and Principe | 0.85 | 4.20 | 4.92 | 21.64 | 114.73 | 5.30 |
| Saudi Arabia | 578.22 | 2451.64 | 4.24 | 14558.46 | 83353.83 | 5.73 |
| Senegal | 52.80 | 199.50 | 3.78 | 1396.05 | 5047.45 | 3.62 |
| Serbia | 2478.16 | 3059.10 | 1.23 | 48978.14 | 53251.71 | 1.09 |
| Seychelles | 2.82 | 3.58 | 1.27 | 67.79 | 98.70 | 1.46 |
| Sierra Leone | 26.05 | 73.25 | 2.81 | 631.22 | 1947.79 | 3.09 |
| Singapore | 134.54 | 95.93 | 0.71 | 3514.79 | 2967.34 | 0.84 |
| Slovakia | 755.30 | 516.96 | 0.68 | 16716.05 | 11164.59 | 0.67 |
| Slovenia | 329.72 | 140.54 | 0.43 | 6307.10 | 2413.41 | 0.38 |
| Solomon Islands | 0.70 | 4.31 | 6.12 | 21.81 | 128.03 | 5.87 |
| Somalia | 6.98 | 24.87 | 3.56 | 191.60 | 665.17 | 3.47 |
| South Africa | 898.48 | 2668.11 | 2.97 | 23359.63 | 62581.97 | 2.68 |
| Republic of Korea | 3413.64 | 3588.89 | 1.05 | 83840.36 | 79886.47 | 0.95 |
| South Sudan | 24.32 | 57.52 | 2.36 | 607.53 | 1484.42 | 2.44 |
| Spain | 2583.60 | 963.49 | 0.37 | 43250.75 | 14963.82 | 0.35 |
| Sri Lanka | 394.30 | 1010.38 | 2.56 | 8796.64 | 23413.45 | 2.66 |
| Sudan | 268.30 | 2571.47 | 9.58 | 6276.96 | 66239.93 | 10.55 |
| Suriname | 14.14 | 32.93 | 2.33 | 294.36 | 693.39 | 2.36 |
| Eswatini | 7.17 | 28.96 | 4.04 | 166.39 | 686.83 | 4.13 |
| Sweden | 358.59 | 80.15 | 0.22 | 6283.37 | 1414.07 | 0.23 |
| Switzerland | 505.49 | 146.78 | 0.29 | 8090.29 | 2368.21 | 0.29 |
| Syrian Arab Republic | 567.40 | 1067.26 | 1.88 | 15128.33 | 28270.94 | 1.87 |
| Taiwan (Province of China) | 852.71 | 929.67 | 1.09 | 22200.63 | 26836.94 | 1.21 |
| Tajikistan | 42.94 | 191.92 | 4.47 | 997.89 | 4937.31 | 4.95 |
| United Republic of Tanzania | 62.88 | 470.30 | 7.48 | 1582.33 | 11024.89 | 6.97 |
| Thailand | 1158.43 | 3311.47 | 2.86 | 33917.93 | 92306.20 | 2.72 |
| Bahamas | 5.72 | 9.70 | 1.70 | 131.74 | 220.66 | 1.67 |
| Gambia | 4.01 | 29.30 | 7.31 | 112.74 | 733.54 | 6.51 |
| Timor-Leste | 1.49 | 23.94 | 16.07 | 42.33 | 574.78 | 13.58 |
| Togo | 18.55 | 88.02 | 4.75 | 522.00 | 2571.30 | 4.93 |
| Tonga | 0.50 | 1.33 | 2.65 | 14.79 | 34.49 | 2.33 |
| Trinidad and Tobago | 77.66 | 98.32 | 1.27 | 1653.95 | 1998.29 | 1.21 |
| Tunisia | 363.58 | 1310.50 | 3.60 | 8302.07 | 29188.65 | 3.52 |
| Turkey | 1558.92 | 4867.88 | 3.12 | 39401.40 | 106530.59 | 2.70 |
| Turkmenistan | 187.82 | 498.47 | 2.65 | 4850.79 | 13362.27 | 2.75 |
| Uganda | 38.71 | 237.80 | 6.14 | 955.26 | 5941.26 | 6.22 |
| Ukraine | 12945.70 | 6868.12 | 0.53 | 260083.12 | 140468.69 | 0.54 |
| United Arab Emirates | 68.91 | 448.39 | 6.51 | 2541.04 | 21178.75 | 8.33 |
| United Kingdom | 5589.70 | 1420.47 | 0.25 | 92646.40 | 22285.82 | 0.24 |
| United States of America | 7743.18 | 3157.22 | 0.41 | 170086.70 | 79931.47 | 0.47 |
| Uruguay | 162.24 | 110.07 | 0.68 | 3261.53 | 1910.14 | 0.59 |
| Uzbekistan | 1011.18 | 2657.32 | 2.63 | 26112.04 | 76529.55 | 2.93 |
| Vanuatu | 0.70 | 3.49 | 4.96 | 21.23 | 101.90 | 4.80 |
| Venezuela (Bolivarian Republic of) | 319.69 | 879.79 | 2.75 | 7074.05 | 17453.10 | 2.47 |
| Viet nam | 1243.34 | 7031.90 | 5.66 | 26227.72 | 154416.62 | 5.89 |
| United States Virgin Islands | 1.18 | 2.43 | 2.06 | 27.22 | 49.39 | 1.81 |
| Yemen | 108.03 | 1500.50 | 13.89 | 2743.92 | 39525.60 | 14.40 |
| Zambia | 25.40 | 228.49 | 8.99 | 636.44 | 5428.85 | 8.53 |
| Zimbabwe | 71.46 | 183.65 | 2.57 | 1699.31 | 4597.98 | 2.71 |
| Monaco | 2.56 | 1.90 | 0.74 | 37.68 | 29.74 | 0.79 |
| San Marino | 0.86 | 0.94 | 1.10 | 15.04 | 14.46 | 0.96 |
| Saint Kitts and Nevis | 2.43 | 1.69 | 0.70 | 45.79 | 36.27 | 0.79 |
| Cook Islands | 0.13 | 0.17 | 1.38 | 3.85 | 5.13 | 1.33 |
| Nauru | 0.07 | 0.06 | 0.91 | 2.44 | 2.49 | 1.02 |
| Niue | 0.04 | 0.03 | 0.75 | 0.91 | 0.76 | 0.84 |
| Palau | 0.15 | 0.27 | 1.86 | 4.58 | 8.99 | 1.96 |
| Tokelau | 0.02 | 0.01 | 0.66 | 0.49 | 0.35 | 0.72 |
| Tuvalu | 0.05 | 0.13 | 2.63 | 1.47 | 3.73 | 2.53 |
| Afghanistan | 82.54 | 385.48 | 4.67 | 1874.91 | 10592.03 | 5.65 |
| Albania | 31.30 | 73.18 | 2.34 | 767.43 | 1544.67 | 2.01 |
| Algeria | 652.27 | 1828.10 | 2.80 | 15294.00 | 42377.28 | 2.77 |
| American Samoa | 0.15 | 0.21 | 1.43 | 4.76 | 6.24 | 1.31 |
| Andorra | 0.50 | 0.47 | 0.94 | 11.69 | 9.16 | 0.78 |
| Angola | 19.49 | 197.95 | 10.16 | 530.00 | 5352.78 | 10.10 |
| Antigua and Barbuda | 1.31 | 1.31 | 1.00 | 25.12 | 27.45 | 1.09 |
| Argentina | 609.81 | 476.29 | 0.78 | 12968.56 | 9693.23 | 0.75 |
| Armenia | 124.02 | 153.99 | 1.24 | 2976.93 | 3381.66 | 1.14 |
| Australia | 115.97 | 74.48 | 0.64 | 2343.38 | 1354.54 | 0.58 |
| Austria | 396.80 | 94.47 | 0.24 | 7267.26 | 1838.96 | 0.25 |
| Azerbaijan | 88.35 | 291.99 | 3.31 | 2407.37 | 7358.06 | 3.06 |
| Bahrain | 8.25 | 23.10 | 2.80 | 263.11 | 898.45 | 3.41 |
| Bangladesh | 669.34 | 5924.11 | 8.85 | 13755.76 | 113644.85 | 8.26 |
| Barbados | 10.07 | 9.99 | 0.99 | 186.59 | 191.60 | 1.03 |
| Belarus | 686.62 | 548.14 | 0.80 | 16811.13 | 12899.11 | 0.77 |
| Belgium | 412.51 | 150.89 | 0.37 | 7730.99 | 2655.48 | 0.34 |
| Belize | 1.04 | 3.92 | 3.76 | 21.44 | 90.85 | 4.24 |
| Benin | 14.41 | 53.12 | 3.68 | 332.96 | 1377.31 | 4.14 |
| Bermuda | 0.68 | 0.45 | 0.66 | 14.90 | 8.59 | 0.58 |
| Bhutan | 0.91 | 11.25 | 12.41 | 22.77 | 234.34 | 10.29 |
| Bolivia (Plurinational State of) | 62.44 | 146.58 | 2.35 | 1383.31 | 3072.30 | 2.22 |
| Bosnia and Herzegovina | 213.72 | 450.87 | 2.11 | 5484.98 | 9269.61 | 1.69 |
| Botswana | 10.49 | 43.46 | 4.14 | 260.16 | 1114.34 | 4.28 |
| Brazil | 2247.18 | 2721.83 | 1.21 | 54823.90 | 58768.04 | 1.07 |
| Brunei Darussalam | 1.25 | 1.23 | 0.99 | 35.26 | 39.21 | 1.11 |
| Bulgaria | 1145.12 | 1110.89 | 0.97 | 25053.32 | 21741.12 | 0.87 |
| Burkina Faso | 11.48 | 46.37 | 4.04 | 302.87 | 1246.48 | 4.12 |
| Burundi | 13.16 | 26.45 | 2.01 | 315.64 | 667.83 | 2.12 |
| Cambodia | 27.09 | 169.29 | 6.25 | 652.74 | 3922.29 | 6.01 |
| Cameroon | 44.71 | 255.87 | 5.72 | 1183.44 | 7192.50 | 6.08 |
| Canada | 220.58 | 117.10 | 0.53 | 5599.93 | 3092.12 | 0.55 |
| Cabo Verde | 1.50 | 14.46 | 9.62 | 35.87 | 321.61 | 8.97 |
| Central African Republic | 7.55 | 18.98 | 2.51 | 204.40 | 536.73 | 2.63 |
| Chad | 11.88 | 45.47 | 3.83 | 287.12 | 1185.55 | 4.13 |
| Chile | 265.13 | 421.86 | 1.59 | 5638.52 | 8155.05 | 1.45 |
| China | 26506.57 | 146681.72 | 5.53 | 663950.51 | 3318210.36 | 5.00 |
| Colombia | 335.95 | 482.77 | 1.44 | 7829.53 | 9730.66 | 1.24 |
| Comoros | 0.88 | 3.86 | 4.40 | 20.86 | 88.27 | 4.23 |
| Congo | 15.68 | 75.38 | 4.81 | 406.56 | 1924.13 | 4.73 |
| Costa Rica | 18.28 | 39.77 | 2.18 | 359.29 | 792.04 | 2.20 |
| Côte d'Ivoire | 39.57 | 168.19 | 4.25 | 1232.77 | 5027.06 | 4.08 |
| Croatia | 395.56 | 256.31 | 0.65 | 8161.08 | 4723.53 | 0.58 |
| Cuba | 238.42 | 368.92 | 1.55 | 5012.45 | 7245.51 | 1.45 |
| Cyprus | 17.38 | 17.08 | 0.98 | 318.20 | 307.92 | 0.97 |
| Czechia | 1294.61 | 374.29 | 0.29 | 26832.46 | 7731.79 | 0.29 |
| Democratic Republic of the Congo | 101.61 | 308.26 | 3.03 | 2508.31 | 7821.09 | 3.12 |
| Denmark | 197.74 | 66.13 | 0.33 | 3821.61 | 1219.26 | 0.32 |
| Djibouti | 1.24 | 20.45 | 16.50 | 37.97 | 566.82 | 14.93 |
| Dominica | 1.20 | 1.77 | 1.47 | 22.81 | 34.94 | 1.53 |
| Dominican Republic | 30.69 | 210.68 | 6.86 | 754.45 | 4802.65 | 6.37 |
| Ecuador | 80.70 | 186.68 | 2.31 | 1903.43 | 4155.11 | 2.18 |
| Egypt | 1616.35 | 5326.77 | 3.30 | 45819.81 | 159103.14 | 3.47 |
| El Salvador | 26.68 | 61.08 | 2.29 | 559.48 | 1178.47 | 2.11 |
| Equatorial Guinea | 1.21 | 13.39 | 11.03 | 31.08 | 338.70 | 10.90 |
| Eritrea | 3.93 | 28.46 | 7.25 | 125.28 | 831.27 | 6.64 |
| Estonia | 63.03 | 8.33 | 0.13 | 1442.90 | 183.99 | 0.13 |
| Ethiopia | 37.26 | 239.19 | 6.42 | 998.19 | 5431.85 | 5.44 |
| Micronesia (Federated States of) | 0.39 | 0.82 | 2.09 | 11.83 | 26.20 | 2.21 |
| Fiji | 2.90 | 8.82 | 3.04 | 88.54 | 269.27 | 3.04 |
| Finland | 76.73 | 22.75 | 0.30 | 1643.06 | 414.81 | 0.25 |
| France | 1648.47 | 653.98 | 0.40 | 29537.77 | 11939.53 | 0.40 |
| Gabon | 9.73 | 37.69 | 3.87 | 243.95 | 959.68 | 3.93 |
| Georgia | 201.34 | 239.55 | 1.19 | 4254.20 | 4603.31 | 1.08 |
| Germany | 3940.76 | 1379.36 | 0.35 | 75173.73 | 26200.13 | 0.35 |
| Ghana | 79.49 | 497.96 | 6.26 | 2177.02 | 13778.30 | 6.33 |
| Greece | 637.46 | 367.85 | 0.58 | 10282.70 | 5202.28 | 0.51 |
| Greenland | 0.29 | 0.29 | 0.99 | 9.16 | 8.16 | 0.89 |
| Grenada | 2.89 | 2.90 | 1.00 | 53.41 | 68.00 | 1.27 |
| Guam | 0.61 | 0.94 | 1.54 | 20.08 | 31.50 | 1.57 |
| Guatemala | 21.44 | 107.68 | 5.02 | 462.30 | 2071.10 | 4.48 |
| Guinea | 15.24 | 53.70 | 3.52 | 360.47 | 1323.51 | 3.67 |
| Guinea-Bissau | 2.87 | 8.44 | 2.94 | 78.45 | 250.40 | 3.19 |
| Guyana | 24.03 | 25.44 | 1.06 | 582.25 | 600.80 | 1.03 |
| Haiti | 22.66 | 79.68 | 3.52 | 529.18 | 1764.05 | 3.33 |
| Honduras | 17.02 | 124.08 | 7.29 | 358.00 | 2448.10 | 6.84 |
| Hungary | 1077.39 | 442.11 | 0.41 | 25716.68 | 10066.90 | 0.39 |
| Iceland | 1.59 | 0.68 | 0.43 | 30.73 | 12.87 | 0.42 |
| India | 6555.89 | 32467.01 | 4.95 | 162466.41 | 763489.66 | 4.70 |
| Indonesia | 1830.93 | 8372.26 | 4.57 | 50050.82 | 215230.85 | 4.30 |
| Iran (Islamic Republic of) | 1883.67 | 3783.69 | 2.01 | 51445.73 | 91563.01 | 1.78 |
| Iraq | 781.20 | 2474.87 | 3.17 | 19186.57 | 61720.82 | 3.22 |
| Ireland | 76.30 | 22.07 | 0.29 | 1433.28 | 393.15 | 0.27 |
| Israel | 104.74 | 88.30 | 0.84 | 2112.32 | 1808.81 | 0.86 |
| Italy | 3386.06 | 1348.74 | 0.40 | 60086.51 | 20724.51 | 0.34 |
| Jamaica | 32.13 | 58.93 | 1.83 | 575.60 | 1060.88 | 1.84 |
| Japan | 2912.43 | 2852.51 | 0.98 | 60094.30 | 58429.32 | 0.97 |
| Jordan | 80.43 | 256.01 | 3.18 | 2228.37 | 7167.05 | 3.22 |
| Kazakhstan | 581.47 | 851.30 | 1.46 | 14910.22 | 20795.58 | 1.39 |
| Kenya | 35.88 | 204.89 | 5.71 | 919.59 | 5431.33 | 5.91 |
| Kiribati | 0.32 | 0.76 | 2.40 | 9.95 | 24.67 | 2.48 |
| Kuwait | 23.84 | 130.93 | 5.49 | 868.41 | 3690.37 | 4.25 |
| Kyrgyzstan | 126.36 | 207.40 | 1.64 | 2971.63 | 5367.66 | 1.81 |
| Lao People's Democratic Republic | 18.34 | 86.49 | 4.71 | 464.83 | 2117.11 | 4.55 |
| Latvia | 235.96 | 97.66 | 0.41 | 5023.70 | 1962.99 | 0.39 |
| Lebanon | 40.92 | 121.57 | 2.97 | 1066.72 | 2925.07 | 2.74 |
| Lesotho | 9.35 | 26.93 | 2.88 | 220.28 | 678.07 | 3.08 |
| Liberia | 7.60 | 19.56 | 2.57 | 183.62 | 519.19 | 2.83 |
| Libya | 67.55 | 266.29 | 3.94 | 1643.64 | 7121.23 | 4.33 |
| Lithuania | 154.45 | 87.50 | 0.57 | 3684.18 | 1841.14 | 0.50 |
| Luxembourg | 17.24 | 4.49 | 0.26 | 319.18 | 83.32 | 0.26 |
| North Macedonia | 273.82 | 410.50 | 1.50 | 5408.48 | 7721.58 | 1.43 |
| Madagascar | 19.58 | 77.76 | 3.97 | 494.81 | 2169.49 | 4.38 |
| Malawi | 12.31 | 52.37 | 4.25 | 297.69 | 1296.54 | 4.36 |
| Malaysia | 340.95 | 498.51 | 1.46 | 9380.30 | 14186.51 | 1.51 |
| Maldives | 0.82 | 2.77 | 3.39 | 23.00 | 78.03 | 3.39 |
| Mali | 13.69 | 56.05 | 4.09 | 338.09 | 1288.70 | 3.81 |
| Malta | 10.08 | 6.98 | 0.69 | 194.51 | 123.58 | 0.64 |
| Marshall Islands | 0.11 | 0.32 | 3.00 | 3.07 | 10.57 | 3.44 |
| Mauritania | 14.09 | 44.90 | 3.19 | 386.81 | 1126.72 | 2.91 |
| Mauritius | 27.86 | 27.45 | 0.99 | 741.20 | 679.04 | 0.92 |
| Mexico | 819.83 | 1187.34 | 1.45 | 18346.14 | 26947.49 | 1.47 |
| Republic of Moldova | 140.13 | 153.77 | 1.10 | 3113.82 | 3454.75 | 1.11 |
| Mongolia | 7.35 | 54.72 | 7.44 | 230.66 | 1771.48 | 7.68 |
| Montenegro | 14.60 | 24.92 | 1.71 | 320.30 | 499.98 | 1.56 |
| Morocco | 375.18 | 2281.57 | 6.08 | 9182.56 | 53490.40 | 5.83 |
| Mozambique | 15.85 | 100.65 | 6.35 | 389.24 | 2636.71 | 6.77 |
| Myanmar | 453.17 | 1781.92 | 3.93 | 11104.38 | 40682.53 | 3.66 |
| Namibia | 14.91 | 45.26 | 3.04 | 339.65 | 993.36 | 2.92 |
| Nepal | 82.93 | 704.00 | 8.49 | 1881.50 | 14562.44 | 7.74 |
| Netherlands | 444.79 | 218.49 | 0.49 | 8557.02 | 4179.00 | 0.49 |
| New Zealand | 20.52 | 13.07 | 0.64 | 404.07 | 227.66 | 0.56 |
| Nicaragua | 9.71 | 48.05 | 4.95 | 204.53 | 981.73 | 4.80 |
| Niger | 11.55 | 43.17 | 3.74 | 322.48 | 1124.10 | 3.49 |
| Nigeria | 416.73 | 1694.56 | 4.07 | 10334.98 | 41021.23 | 3.97 |
| Democratic People's Republic of Korea | 314.98 | 1443.03 | 4.58 | 9054.22 | 37783.69 | 4.17 |
| Northern Mariana Islands | 0.19 | 0.31 | 1.58 | 7.75 | 10.04 | 1.29 |
| Norway | 115.63 | 18.43 | 0.16 | 2121.96 | 411.26 | 0.19 |
| Oman | 43.66 | 117.54 | 2.69 | 1259.23 | 3642.39 | 2.89 |
| Pakistan | 856.07 | 3810.63 | 4.45 | 17784.54 | 94194.38 | 5.30 |
| Palestine | 42.54 | 141.06 | 3.32 | 910.18 | 3469.98 | 3.81 |
| Panama | 20.63 | 37.43 | 1.81 | 398.86 | 676.97 | 1.70 |
| Papua New Guinea | 4.70 | 19.72 | 4.20 | 150.00 | 640.94 | 4.27 |
| Paraguay | 28.41 | 70.42 | 2.48 | 576.87 | 1483.73 | 2.57 |
| Peru | 194.92 | 364.77 | 1.87 | 4024.99 | 7464.56 | 1.85 |
| Philippines | 290.86 | 1345.89 | 4.63 | 8563.52 | 39107.62 | 4.57 |
| Poland | 2145.91 | 1853.84 | 0.86 | 52116.75 | 40656.29 | 0.78 |
| Portugal | 533.97 | 163.57 | 0.31 | 9651.22 | 2540.10 | 0.26 |
| Puerto Rico | 13.81 | 11.10 | 0.80 | 273.51 | 215.19 | 0.79 |
| Qatar | 5.13 | 18.07 | 3.52 | 236.94 | 1196.07 | 5.05 |
| Romania | 1733.02 | 1511.46 | 0.87 | 37217.28 | 28647.15 | 0.77 |
| Russian Federation | 10876.99 | 6832.04 | 0.63 | 259452.26 | 153689.23 | 0.59 |
| Rwanda | 19.19 | 51.70 | 2.69 | 481.10 | 1272.44 | 2.64 |
| Saint Lucia | 2.64 | 5.32 | 2.02 | 54.38 | 107.07 | 1.97 |
| Saint Vincent and the Grenadines | 2.08 | 3.24 | 1.56 | 43.93 | 66.63 | 1.52 |
| Samoa | 0.93 | 1.49 | 1.60 | 24.99 | 40.39 | 1.62 |
| Sao Tome and Principe | 0.40 | 1.98 | 5.00 | 10.16 | 54.87 | 5.40 |
| Saudi Arabia | 341.93 | 1463.86 | 4.28 | 8718.54 | 48168.81 | 5.52 |
| Senegal | 29.16 | 101.44 | 3.48 | 779.20 | 2582.04 | 3.31 |
| Serbia | 1152.90 | 1318.43 | 1.14 | 24311.26 | 24600.51 | 1.01 |
| Seychelles | 1.59 | 1.86 | 1.17 | 39.25 | 55.24 | 1.41 |
| Sierra Leone | 15.41 | 38.63 | 2.51 | 375.49 | 1043.67 | 2.78 |
| Singapore | 61.85 | 42.18 | 0.68 | 1656.30 | 1359.99 | 0.82 |
| Slovakia | 343.97 | 237.21 | 0.69 | 8098.09 | 5555.36 | 0.69 |
| Slovenia | 142.56 | 62.40 | 0.44 | 2940.64 | 1167.89 | 0.40 |
| Solomon Islands | 0.47 | 2.47 | 5.22 | 14.17 | 73.33 | 5.18 |
| Somalia | 4.01 | 12.61 | 3.15 | 107.21 | 345.74 | 3.22 |
| South Africa | 424.56 | 1090.95 | 2.57 | 11418.06 | 27854.70 | 2.44 |
| Republic of Korea | 1702.14 | 1719.73 | 1.01 | 43709.85 | 40983.89 | 0.94 |
| South Sudan | 14.40 | 35.30 | 2.45 | 363.25 | 868.29 | 2.39 |
| Spain | 1075.48 | 379.24 | 0.35 | 19718.30 | 6589.66 | 0.33 |
| Sri Lanka | 257.42 | 525.30 | 2.04 | 5707.21 | 12473.66 | 2.19 |
| Sudan | 156.69 | 1419.22 | 9.06 | 3613.44 | 35279.15 | 9.76 |
| Suriname | 6.95 | 15.41 | 2.22 | 153.63 | 347.24 | 2.26 |
| Eswatini | 3.62 | 12.95 | 3.58 | 88.75 | 333.31 | 3.76 |
| Sweden | 160.42 | 35.76 | 0.22 | 3096.79 | 696.30 | 0.22 |
| Switzerland | 213.17 | 61.25 | 0.29 | 3787.60 | 1083.59 | 0.29 |
| Syrian Arab Republic | 292.69 | 550.43 | 1.88 | 7964.66 | 14510.22 | 1.82 |
| Taiwan (Province of China) | 468.70 | 502.00 | 1.07 | 12221.73 | 13048.70 | 1.07 |
| Tajikistan | 22.78 | 99.53 | 4.37 | 529.43 | 2491.97 | 4.71 |
| United Republic of Tanzania | 37.74 | 270.26 | 7.16 | 927.84 | 6354.18 | 6.85 |
| Thailand | 687.58 | 1732.08 | 2.52 | 19735.84 | 48437.48 | 2.45 |
| Bahamas | 2.38 | 4.16 | 1.75 | 58.27 | 100.76 | 1.73 |
| Gambia | 2.29 | 15.13 | 6.60 | 67.77 | 394.21 | 5.82 |
| Timor-Leste | 0.88 | 15.05 | 17.02 | 25.12 | 356.12 | 14.18 |
| Togo | 10.17 | 45.91 | 4.52 | 289.70 | 1408.22 | 4.86 |
| Tonga | 0.30 | 0.64 | 2.09 | 8.31 | 16.41 | 1.97 |
| Trinidad and Tobago | 41.30 | 51.01 | 1.24 | 926.52 | 1098.76 | 1.19 |
| Tunisia | 200.53 | 659.55 | 3.29 | 4519.57 | 14646.86 | 3.24 |
| Turkey | 762.59 | 2170.85 | 2.85 | 19333.00 | 49339.03 | 2.55 |
| Turkmenistan | 82.87 | 257.34 | 3.11 | 2215.24 | 7165.99 | 3.23 |
| Uganda | 22.34 | 122.36 | 5.48 | 551.05 | 3127.19 | 5.67 |
| Ukraine | 4654.14 | 2982.66 | 0.64 | 104913.16 | 69538.53 | 0.66 |
| United Arab Emirates | 43.69 | 336.84 | 7.71 | 1668.57 | 15525.25 | 9.30 |
| United Kingdom | 2236.12 | 609.19 | 0.27 | 41619.83 | 10434.08 | 0.25 |
| United States of America | 3086.64 | 1245.18 | 0.40 | 71657.80 | 35016.75 | 0.49 |
| Uruguay | 75.80 | 44.93 | 0.59 | 1635.30 | 860.47 | 0.53 |
| Uzbekistan | 530.72 | 1471.79 | 2.77 | 14188.10 | 42446.85 | 2.99 |
| Vanuatu | 0.49 | 2.21 | 4.46 | 14.09 | 61.90 | 4.39 |
| Venezuela (Bolivarian Republic of) | 156.92 | 446.11 | 2.84 | 3502.11 | 9021.28 | 2.58 |
| Viet nam | 734.93 | 4149.92 | 5.65 | 16536.34 | 98203.86 | 5.94 |
| United States Virgin Islands | 0.50 | 1.24 | 2.46 | 12.23 | 26.56 | 2.17 |
| Yemen | 57.77 | 782.73 | 13.55 | 1547.20 | 20276.22 | 13.11 |
| Zambia | 14.56 | 118.93 | 8.17 | 342.20 | 2849.12 | 8.33 |
| Zimbabwe | 36.78 | 83.79 | 2.28 | 905.52 | 2126.06 | 2.35 |
| Monaco | 0.94 | 0.74 | 0.79 | 15.57 | 12.69 | 0.82 |
| San Marino | 0.41 | 0.44 | 1.09 | 7.67 | 7.20 | 0.94 |
| Saint Kitts and Nevis | 0.97 | 0.76 | 0.78 | 18.98 | 18.38 | 0.97 |
| Cook Islands | 0.07 | 0.09 | 1.24 | 2.12 | 2.58 | 1.22 |
| Nauru | 0.04 | 0.03 | 0.69 | 1.54 | 1.17 | 0.76 |
| Niue | 0.02 | 0.01 | 0.77 | 0.47 | 0.37 | 0.80 |
| Palau | 0.09 | 0.17 | 1.87 | 2.76 | 5.53 | 2.00 |
| Tokelau | 0.01 | 0.01 | 0.63 | 0.20 | 0.14 | 0.72 |
| Tuvalu | 0.03 | 0.06 | 2.13 | 0.83 | 1.76 | 2.12 |
| Afghanistan | 60.94 | 422.44 | 6.93 | 1515.41 | 12223.18 | 8.07 |
| Albania | 26.28 | 69.51 | 2.65 | 619.47 | 1479.27 | 2.39 |
| Algeria | 599.07 | 2016.78 | 3.37 | 15185.74 | 48205.57 | 3.17 |
| American Samoa | 0.11 | 0.25 | 2.32 | 3.77 | 7.96 | 2.11 |
| Andorra | 0.37 | 0.47 | 1.28 | 8.35 | 8.47 | 1.01 |
| Angola | 11.49 | 172.24 | 14.99 | 325.28 | 4671.92 | 14.36 |
| Antigua and Barbuda | 1.78 | 2.12 | 1.19 | 29.63 | 40.68 | 1.37 |
| Argentina | 582.54 | 526.62 | 0.90 | 11220.87 | 10017.10 | 0.89 |
| Armenia | 150.55 | 197.96 | 1.31 | 3355.53 | 3873.00 | 1.15 |
| Australia | 148.83 | 102.15 | 0.69 | 2608.28 | 1719.78 | 0.66 |
| Austria | 646.14 | 124.00 | 0.19 | 9826.95 | 2138.64 | 0.22 |
| Azerbaijan | 96.90 | 332.06 | 3.43 | 2470.94 | 7980.37 | 3.23 |
| Bahrain | 8.89 | 21.01 | 2.36 | 257.25 | 687.33 | 2.67 |
| Bangladesh | 313.47 | 3396.37 | 10.83 | 6424.66 | 64760.89 | 10.08 |
| Barbados | 16.85 | 15.79 | 0.94 | 266.64 | 266.36 | 1.00 |
| Belarus | 1143.05 | 793.62 | 0.69 | 23083.43 | 14285.27 | 0.62 |
| Belgium | 620.96 | 218.49 | 0.35 | 9735.03 | 3287.51 | 0.34 |
| Belize | 1.09 | 3.28 | 3.00 | 20.28 | 74.59 | 3.68 |
| Benin | 11.60 | 50.85 | 4.38 | 258.43 | 1209.02 | 4.68 |
| Bermuda | 0.81 | 0.41 | 0.50 | 15.22 | 7.07 | 0.46 |
| Bhutan | 0.78 | 7.88 | 10.05 | 18.35 | 167.44 | 9.13 |
| Bolivia (Plurinational State of) | 57.53 | 151.23 | 2.63 | 1307.79 | 3180.69 | 2.43 |
| Bosnia and Herzegovina | 233.82 | 563.84 | 2.41 | 5262.88 | 10235.17 | 1.94 |
| Botswana | 8.27 | 47.72 | 5.77 | 187.43 | 1102.70 | 5.88 |
| Brazil | 1574.09 | 2315.32 | 1.47 | 34897.98 | 45300.24 | 1.30 |
| Brunei Darussalam | 0.98 | 1.13 | 1.15 | 29.32 | 38.41 | 1.31 |
| Bulgaria | 1094.58 | 1330.56 | 1.22 | 22143.72 | 22247.08 | 1.00 |
| Burkina Faso | 8.11 | 36.99 | 4.56 | 229.89 | 980.08 | 4.26 |
| Burundi | 10.06 | 18.00 | 1.79 | 255.23 | 468.49 | 1.84 |
| Cambodia | 22.78 | 159.27 | 6.99 | 559.46 | 3514.04 | 6.28 |
| Cameroon | 38.99 | 221.21 | 5.67 | 1017.69 | 5915.60 | 5.81 |
| Canada | 293.37 | 166.42 | 0.57 | 5818.25 | 3291.81 | 0.57 |
| Cabo Verde | 1.20 | 12.94 | 10.82 | 28.62 | 261.01 | 9.12 |
| Central African Republic | 5.25 | 16.58 | 3.16 | 137.33 | 423.52 | 3.08 |
| Chad | 10.11 | 29.69 | 2.94 | 236.97 | 783.37 | 3.31 |
| Chile | 240.34 | 429.03 | 1.79 | 4936.90 | 7839.89 | 1.59 |
| China | 18127.02 | 98697.12 | 5.44 | 460089.30 | 2419540.66 | 5.26 |
| Colombia | 294.00 | 523.03 | 1.78 | 6931.53 | 10768.30 | 1.55 |
| Comoros | 0.77 | 4.50 | 5.85 | 19.54 | 104.06 | 5.33 |
| Congo | 13.54 | 84.94 | 6.27 | 355.81 | 2183.52 | 6.14 |
| Costa Rica | 14.86 | 42.49 | 2.86 | 310.95 | 855.10 | 2.75 |
| Côte d'Ivoire | 21.32 | 112.46 | 5.27 | 647.48 | 3269.69 | 5.05 |
| Croatia | 541.01 | 354.28 | 0.65 | 9773.12 | 5536.26 | 0.57 |
| Cuba | 243.44 | 382.79 | 1.57 | 5021.03 | 6764.80 | 1.35 |
| Cyprus | 27.43 | 24.83 | 0.91 | 432.28 | 382.18 | 0.88 |
| Czechia | 1730.38 | 494.62 | 0.29 | 30186.24 | 8655.63 | 0.29 |
| Democratic Republic of the Congo | 70.98 | 314.40 | 4.43 | 1933.10 | 7298.30 | 3.78 |
| Denmark | 237.27 | 70.94 | 0.30 | 3930.87 | 1128.69 | 0.29 |
| Djibouti | 0.93 | 15.80 | 16.91 | 28.98 | 453.95 | 15.67 |
| Dominica | 1.91 | 2.67 | 1.40 | 32.15 | 43.72 | 1.36 |
| Dominican Republic | 22.15 | 194.34 | 8.77 | 527.76 | 4108.31 | 7.78 |
| Ecuador | 64.12 | 183.81 | 2.87 | 1543.80 | 4043.00 | 2.62 |
| Egypt | 1620.54 | 5197.22 | 3.21 | 44957.03 | 157973.43 | 3.51 |
| El Salvador | 19.35 | 65.84 | 3.40 | 401.30 | 1313.03 | 3.27 |
| Equatorial Guinea | 0.89 | 19.52 | 21.82 | 22.87 | 486.28 | 21.27 |
| Eritrea | 3.71 | 36.01 | 9.72 | 114.46 | 938.70 | 8.20 |
| Estonia | 104.33 | 13.33 | 0.13 | 1914.70 | 239.05 | 0.12 |
| Ethiopia | 23.22 | 189.32 | 8.15 | 689.99 | 4762.19 | 6.90 |
| Micronesia (Federated States of) | 0.22 | 0.73 | 3.29 | 6.92 | 22.48 | 3.25 |
| Fiji | 1.61 | 8.24 | 5.12 | 56.16 | 262.97 | 4.68 |
| Finland | 118.70 | 30.43 | 0.26 | 2046.32 | 462.17 | 0.23 |
| France | 2240.72 | 870.13 | 0.39 | 31888.08 | 13009.25 | 0.41 |
| Gabon | 9.19 | 42.98 | 4.68 | 209.44 | 980.95 | 4.68 |
| Georgia | 286.44 | 268.51 | 0.94 | 5378.92 | 4448.90 | 0.83 |
| Germany | 6402.53 | 1759.58 | 0.27 | 101761.92 | 28230.24 | 0.28 |
| Ghana | 124.02 | 825.72 | 6.66 | 3309.13 | 20729.68 | 6.26 |
| Greece | 900.94 | 588.61 | 0.65 | 13266.98 | 7255.81 | 0.55 |
| Greenland | 0.34 | 0.20 | 0.59 | 7.91 | 4.84 | 0.61 |
| Grenada | 3.40 | 4.14 | 1.22 | 56.13 | 78.61 | 1.40 |
| Guam | 0.49 | 0.87 | 1.79 | 17.93 | 32.14 | 1.79 |
| Guatemala | 15.89 | 96.07 | 6.04 | 398.42 | 2057.49 | 5.16 |
| Guinea | 14.96 | 45.60 | 3.05 | 352.43 | 1072.79 | 3.04 |
| Guinea-Bissau | 2.00 | 7.77 | 3.88 | 57.61 | 220.84 | 3.83 |
| Guyana | 17.79 | 25.30 | 1.42 | 390.80 | 543.50 | 1.39 |
| Haiti | 22.01 | 93.49 | 4.25 | 501.04 | 2123.10 | 4.24 |
| Honduras | 10.22 | 94.44 | 9.24 | 249.21 | 1966.82 | 7.89 |
| Hungary | 1197.54 | 572.17 | 0.48 | 24646.14 | 10931.28 | 0.44 |
| Iceland | 1.74 | 0.66 | 0.38 | 29.83 | 11.43 | 0.38 |
| India | 3575.20 | 25568.62 | 7.15 | 89162.69 | 587694.92 | 6.59 |
| Indonesia | 1528.04 | 7838.24 | 5.13 | 43016.14 | 193553.03 | 4.50 |
| Iran (Islamic Republic of) | 1548.26 | 3664.68 | 2.37 | 42058.92 | 87112.09 | 2.07 |
| Iraq | 673.27 | 2225.15 | 3.30 | 16391.16 | 57263.74 | 3.49 |
| Ireland | 89.63 | 26.86 | 0.30 | 1456.37 | 425.19 | 0.29 |
| Israel | 117.43 | 118.58 | 1.01 | 2266.01 | 2109.01 | 0.93 |
| Italy | 4466.54 | 2092.71 | 0.47 | 67975.97 | 26853.25 | 0.40 |
| Jamaica | 36.60 | 77.03 | 2.10 | 587.41 | 1200.61 | 2.04 |
| Japan | 3057.57 | 2776.28 | 0.91 | 56366.34 | 50897.98 | 0.90 |
| Jordan | 110.65 | 258.71 | 2.34 | 2882.25 | 7220.80 | 2.51 |
| Kazakhstan | 763.28 | 999.59 | 1.31 | 16433.53 | 20749.63 | 1.26 |
| Kenya | 27.84 | 177.99 | 6.39 | 756.99 | 4787.10 | 6.32 |
| Kiribati | 0.14 | 0.40 | 2.88 | 4.50 | 12.86 | 2.85 |
| Kuwait | 22.86 | 55.58 | 2.43 | 712.03 | 2017.00 | 2.83 |
| Kyrgyzstan | 140.17 | 157.70 | 1.13 | 2845.28 | 3680.69 | 1.29 |
| Lao People's Democratic Republic | 10.15 | 51.32 | 5.05 | 269.66 | 1253.76 | 4.65 |
| Latvia | 407.80 | 182.17 | 0.45 | 7180.63 | 2824.92 | 0.39 |
| Lebanon | 48.09 | 138.95 | 2.89 | 1296.35 | 3777.49 | 2.91 |
| Lesotho | 9.27 | 33.09 | 3.57 | 182.08 | 683.78 | 3.76 |
| Liberia | 5.72 | 16.59 | 2.90 | 141.50 | 442.24 | 3.13 |
| Libya | 65.72 | 292.98 | 4.46 | 1703.61 | 8750.11 | 5.14 |
| Lithuania | 224.36 | 142.67 | 0.64 | 4682.64 | 2380.28 | 0.51 |
| Luxembourg | 24.12 | 5.83 | 0.24 | 379.17 | 90.47 | 0.24 |
| North Macedonia | 271.09 | 475.34 | 1.75 | 5107.91 | 8180.31 | 1.60 |
| Madagascar | 13.18 | 70.29 | 5.33 | 366.96 | 1978.79 | 5.39 |
| Malawi | 9.58 | 39.69 | 4.14 | 244.59 | 922.76 | 3.77 |
| Malaysia | 340.90 | 464.11 | 1.36 | 9428.29 | 13357.79 | 1.42 |
| Maldives | 0.32 | 1.48 | 4.64 | 11.45 | 45.16 | 3.95 |
| Mali | 17.17 | 54.21 | 3.16 | 450.84 | 1393.47 | 3.09 |
| Malta | 11.88 | 8.86 | 0.75 | 214.01 | 138.01 | 0.64 |
| Marshall Islands | 0.06 | 0.25 | 4.10 | 1.96 | 9.49 | 4.84 |
| Mauritania | 17.06 | 52.10 | 3.05 | 432.98 | 1349.08 | 3.12 |
| Mauritius | 20.24 | 25.55 | 1.26 | 488.87 | 603.31 | 1.23 |
| Mexico | 768.28 | 1151.17 | 1.50 | 18607.53 | 26945.11 | 1.45 |
| Republic of Moldova | 175.97 | 173.29 | 0.98 | 3602.05 | 3494.13 | 0.97 |
| Mongolia | 6.08 | 39.66 | 6.52 | 207.22 | 1340.99 | 6.47 |
| Montenegro | 14.24 | 26.80 | 1.88 | 332.07 | 533.49 | 1.61 |
| Morocco | 326.77 | 2406.23 | 7.36 | 8264.00 | 61203.62 | 7.41 |
| Mozambique | 11.10 | 61.33 | 5.52 | 266.29 | 1443.67 | 5.42 |
| Myanmar | 262.47 | 1582.63 | 6.03 | 5767.97 | 30459.62 | 5.28 |
| Namibia | 11.45 | 46.03 | 4.02 | 252.92 | 900.33 | 3.56 |
| Nepal | 39.77 | 321.78 | 8.09 | 928.82 | 6814.87 | 7.34 |
| Netherlands | 631.31 | 300.44 | 0.48 | 10115.82 | 4750.96 | 0.47 |
| New Zealand | 28.06 | 17.88 | 0.64 | 479.93 | 284.10 | 0.59 |
| Nicaragua | 4.87 | 38.05 | 7.81 | 112.91 | 789.98 | 7.00 |
| Niger | 9.72 | 38.93 | 4.01 | 263.67 | 1023.95 | 3.88 |
| Nigeria | 462.88 | 1810.17 | 3.91 | 9865.26 | 46801.34 | 4.74 |
| Democratic People's Republic of Korea | 304.99 | 1360.52 | 4.46 | 8143.01 | 32546.67 | 4.00 |
| Northern Mariana Islands | 0.15 | 0.23 | 1.55 | 6.06 | 8.70 | 1.43 |
| Norway | 150.65 | 24.61 | 0.16 | 2318.36 | 429.48 | 0.19 |
| Oman | 16.01 | 63.18 | 3.95 | 416.89 | 1824.75 | 4.38 |
| Pakistan | 478.59 | 3180.33 | 6.65 | 10441.99 | 78491.67 | 7.52 |
| Palestine | 41.25 | 170.25 | 4.13 | 894.37 | 3883.57 | 4.34 |
| Panama | 15.90 | 33.33 | 2.10 | 309.42 | 605.85 | 1.96 |
| Papua New Guinea | 1.99 | 10.10 | 5.07 | 81.33 | 392.32 | 4.82 |
| Paraguay | 19.14 | 53.78 | 2.81 | 350.32 | 1007.21 | 2.88 |
| Peru | 139.13 | 336.62 | 2.42 | 3033.12 | 7229.82 | 2.38 |
| Philippines | 245.29 | 1157.63 | 4.72 | 7469.70 | 32687.56 | 4.38 |
| Poland | 3207.56 | 2580.09 | 0.80 | 62648.79 | 44741.10 | 0.71 |
| Portugal | 651.38 | 222.99 | 0.34 | 10242.63 | 2992.04 | 0.29 |
| Puerto Rico | 14.81 | 14.12 | 0.95 | 282.91 | 264.02 | 0.93 |
| Qatar | 3.61 | 8.97 | 2.48 | 133.88 | 518.61 | 3.87 |
| Romania | 1965.22 | 1943.99 | 0.99 | 38950.19 | 31765.19 | 0.82 |
| Russian Federation | 21402.49 | 10692.14 | 0.50 | 405330.82 | 184308.43 | 0.45 |
| Rwanda | 15.00 | 51.96 | 3.46 | 393.98 | 1234.87 | 3.13 |
| Saint Lucia | 3.62 | 7.03 | 1.94 | 66.78 | 123.85 | 1.85 |
| Saint Vincent and the Grenadines | 2.86 | 4.36 | 1.53 | 53.92 | 81.08 | 1.50 |
| Samoa | 0.56 | 1.30 | 2.30 | 15.72 | 36.16 | 2.30 |
| Sao Tome and Principe | 0.46 | 2.22 | 4.85 | 11.48 | 59.87 | 5.22 |
| Saudi Arabia | 236.29 | 987.77 | 4.18 | 5839.92 | 35185.03 | 6.02 |
| Senegal | 23.64 | 98.06 | 4.15 | 616.84 | 2465.41 | 4.00 |
| Serbia | 1325.26 | 1740.67 | 1.31 | 24666.88 | 28651.20 | 1.16 |
| Seychelles | 1.24 | 1.72 | 1.39 | 28.55 | 43.46 | 1.52 |
| Sierra Leone | 10.64 | 34.61 | 3.25 | 255.73 | 904.13 | 3.54 |
| Singapore | 72.68 | 53.75 | 0.74 | 1858.49 | 1607.35 | 0.86 |
| Slovakia | 411.32 | 279.75 | 0.68 | 8617.96 | 5609.23 | 0.65 |
| Slovenia | 187.16 | 78.14 | 0.42 | 3366.47 | 1245.52 | 0.37 |
| Solomon Islands | 0.23 | 1.84 | 7.98 | 7.64 | 54.70 | 7.16 |
| Somalia | 2.98 | 12.26 | 4.12 | 84.39 | 319.43 | 3.79 |
| South Africa | 473.93 | 1577.17 | 3.33 | 11941.57 | 34727.28 | 2.91 |
| Republic of Korea | 1711.49 | 1869.15 | 1.09 | 40130.51 | 38902.58 | 0.97 |
| South Sudan | 9.92 | 22.22 | 2.24 | 244.27 | 616.13 | 2.52 |
| Spain | 1508.12 | 584.25 | 0.39 | 23532.45 | 8374.16 | 0.36 |
| Sri Lanka | 136.88 | 485.08 | 3.54 | 3089.43 | 10939.79 | 3.54 |
| Sudan | 111.61 | 1152.26 | 10.32 | 2663.52 | 30960.78 | 11.62 |
| Suriname | 7.19 | 17.52 | 2.44 | 140.73 | 346.15 | 2.46 |
| Eswatini | 3.55 | 16.01 | 4.51 | 77.63 | 353.52 | 4.55 |
| Sweden | 198.17 | 44.39 | 0.22 | 3186.58 | 717.77 | 0.23 |
| Switzerland | 292.32 | 85.53 | 0.29 | 4302.69 | 1284.62 | 0.30 |
| Syrian Arab Republic | 274.71 | 516.83 | 1.88 | 7163.67 | 13760.72 | 1.92 |
| Taiwan (Province of China) | 384.00 | 427.67 | 1.11 | 9978.90 | 13788.24 | 1.38 |
| Tajikistan | 20.16 | 92.39 | 4.58 | 468.46 | 2445.34 | 5.22 |
| United Republic of Tanzania | 25.14 | 200.05 | 7.96 | 654.49 | 4670.71 | 7.14 |
| Thailand | 470.85 | 1579.39 | 3.35 | 14182.10 | 43868.72 | 3.09 |
| Bahamas | 3.34 | 5.54 | 1.66 | 73.46 | 119.90 | 1.63 |
| Gambia | 1.72 | 14.17 | 8.26 | 44.97 | 339.33 | 7.55 |
| Timor-Leste | 0.60 | 8.89 | 14.69 | 17.21 | 218.66 | 12.70 |
| Togo | 8.38 | 42.10 | 5.02 | 232.31 | 1163.08 | 5.01 |
| Tonga | 0.20 | 0.69 | 3.51 | 6.48 | 18.08 | 2.79 |
| Trinidad and Tobago | 36.36 | 47.31 | 1.30 | 727.43 | 899.52 | 1.24 |
| Tunisia | 163.05 | 650.95 | 3.99 | 3782.50 | 14541.79 | 3.84 |
| Turkey | 796.33 | 2697.03 | 3.39 | 20068.40 | 57191.55 | 2.85 |
| Turkmenistan | 104.95 | 241.13 | 2.30 | 2635.55 | 6196.28 | 2.35 |
| Uganda | 16.37 | 115.44 | 7.05 | 404.21 | 2814.07 | 6.96 |
| Ukraine | 8291.56 | 3885.46 | 0.47 | 155169.96 | 70930.16 | 0.46 |
| United Arab Emirates | 25.23 | 111.55 | 4.42 | 872.47 | 5653.50 | 6.48 |
| United Kingdom | 3353.58 | 811.29 | 0.24 | 51026.57 | 11851.75 | 0.23 |
| United States of America | 4656.54 | 1912.05 | 0.41 | 98428.90 | 44914.72 | 0.46 |
| Uruguay | 86.44 | 65.14 | 0.75 | 1626.23 | 1049.67 | 0.65 |
| Uzbekistan | 480.46 | 1185.53 | 2.47 | 11923.94 | 34082.70 | 2.86 |
| Vanuatu | 0.21 | 1.28 | 6.14 | 7.13 | 40.00 | 5.61 |
| Venezuela (Bolivarian Republic of) | 162.77 | 433.69 | 2.66 | 3571.94 | 8431.82 | 2.36 |
| Viet nam | 508.40 | 2881.98 | 5.67 | 9691.38 | 56212.76 | 5.80 |
| United States Virgin Islands | 0.67 | 1.19 | 1.76 | 14.99 | 22.82 | 1.52 |
| Yemen | 50.26 | 717.78 | 14.28 | 1196.72 | 19249.38 | 16.09 |
| Zambia | 10.85 | 109.55 | 10.10 | 294.24 | 2579.73 | 8.77 |
| Zimbabwe | 34.68 | 99.86 | 2.88 | 793.79 | 2471.92 | 3.11 |
| Monaco | 1.62 | 1.16 | 0.71 | 22.11 | 17.04 | 0.77 |
| San Marino | 0.45 | 0.50 | 1.11 | 7.37 | 7.27 | 0.99 |
| Saint Kitts and Nevis | 1.46 | 0.93 | 0.64 | 26.81 | 17.89 | 0.67 |
| Cook Islands | 0.05 | 0.08 | 1.56 | 1.74 | 2.55 | 1.47 |
| Nauru | 0.02 | 0.03 | 1.34 | 0.90 | 1.32 | 1.46 |
| Niue | 0.02 | 0.02 | 0.74 | 0.44 | 0.39 | 0.88 |
| Palau | 0.06 | 0.10 | 1.85 | 1.82 | 3.45 | 1.90 |
| Tokelau | 0.01 | 0.01 | 0.69 | 0.29 | 0.21 | 0.72 |
| Tuvalu | 0.02 | 0.07 | 3.32 | 0.64 | 1.97 | 3.08 |

**Supplementary Table 5 Death Rate of Ischaemic Stroke Attributed to Ambient Particulate Matter Pollution by Age Group in Countries Around the World in 2019**

| **Country**  **Age** | **25-29 years** | **30-34 years** | **35-39 years** | **40-44 years** | **45-49 years** | **50-54 years** | **55-59 years** | **60-64 years** | **65-69 years** | **70-74 years** | **75-79 years** | **80-84 years** | **85+ years** |
| --- | --- | --- | --- | --- | --- | --- | --- | --- | --- | --- | --- | --- | --- |
| Afghanistan | 0.30 | 0.57 | 1.07 | 2.02 | 3.57 | 6.49 | 9.48 | 19.18 | 29.29 | 49.85 | 81.55 | 124.55 | 169.72 |
| Albania | 0.18 | 0.25 | 0.26 | 0.31 | 0.54 | 0.90 | 1.61 | 4.86 | 8.40 | 21.20 | 34.94 | 70.47 | 90.59 |
| Algeria | 0.40 | 0.71 | 1.26 | 2.16 | 3.54 | 6.41 | 10.46 | 22.84 | 37.16 | 64.67 | 127.14 | 228.40 | 563.30 |
| American Samoa | 0.05 | 0.09 | 0.17 | 0.19 | 0.39 | 0.68 | 0.94 | 2.38 | 3.39 | 7.09 | 11.88 | 19.95 | 28.41 |
| Andorra | 0.01 | 0.01 | 0.01 | 0.03 | 0.05 | 0.09 | 0.16 | 0.50 | 0.96 | 2.79 | 5.68 | 13.38 | 25.66 |
| Angola | 0.07 | 0.11 | 0.17 | 0.41 | 0.86 | 1.82 | 3.14 | 10.07 | 16.54 | 36.54 | 56.13 | 98.18 | 109.39 |
| Antigua and Barbuda | 0.05 | 0.07 | 0.13 | 0.24 | 0.51 | 1.15 | 2.30 | 6.10 | 9.61 | 22.29 | 38.69 | 79.99 | 126.11 |
| Argentina | 0.02 | 0.03 | 0.05 | 0.09 | 0.20 | 0.39 | 0.68 | 2.82 | 4.77 | 11.44 | 18.64 | 35.82 | 52.85 |
| Armenia | 0.12 | 0.18 | 0.39 | 0.82 | 2.01 | 3.89 | 6.63 | 14.50 | 26.07 | 57.13 | 91.27 | 165.21 | 217.81 |
| Australia | 0.00 | 0.01 | 0.01 | 0.02 | 0.03 | 0.05 | 0.09 | 0.21 | 0.39 | 1.14 | 2.60 | 7.34 | 19.21 |
| Austria | 0.01 | 0.01 | 0.02 | 0.04 | 0.08 | 0.15 | 0.34 | 0.92 | 1.80 | 4.42 | 8.22 | 20.96 | 44.13 |
| Azerbaijan | 0.06 | 0.15 | 0.30 | 0.63 | 1.60 | 3.44 | 6.07 | 16.63 | 29.65 | 64.02 | 98.12 | 182.93 | 268.80 |
| Bahamas | 0.08 | 0.17 | 0.28 | 0.46 | 0.74 | 1.39 | 2.13 | 4.69 | 7.81 | 17.53 | 30.44 | 57.03 | 77.66 |
| Bahrain | 0.17 | 0.21 | 0.44 | 0.60 | 0.99 | 1.90 | 3.32 | 9.22 | 19.77 | 49.69 | 111.85 | 201.51 | 325.26 |
| Bangladesh | 0.11 | 0.19 | 0.27 | 0.46 | 1.05 | 2.59 | 4.00 | 14.33 | 24.66 | 51.87 | 75.19 | 167.35 | 264.02 |
| Barbados | 0.07 | 0.16 | 0.23 | 0.45 | 0.90 | 1.56 | 2.88 | 7.04 | 12.21 | 31.87 | 51.81 | 97.56 | 166.59 |
| Belarus | 0.05 | 0.13 | 0.28 | 0.71 | 2.00 | 3.95 | 7.79 | 18.17 | 31.59 | 55.43 | 81.83 | 135.35 | 180.26 |
| Belgium | 0.01 | 0.02 | 0.04 | 0.07 | 0.13 | 0.24 | 0.44 | 1.25 | 2.17 | 5.67 | 11.00 | 26.30 | 54.96 |
| Belize | 0.08 | 0.16 | 0.18 | 0.33 | 0.68 | 1.28 | 2.39 | 5.54 | 8.27 | 18.53 | 30.08 | 58.20 | 85.84 |
| Benin | 0.08 | 0.10 | 0.12 | 0.28 | 0.41 | 0.99 | 1.77 | 5.51 | 8.84 | 16.66 | 26.88 | 59.50 | 71.25 |
| Bermuda | 0.01 | 0.01 | 0.02 | 0.04 | 0.10 | 0.22 | 0.38 | 0.86 | 1.41 | 3.19 | 5.53 | 12.26 | 20.41 |
| Bhutan | 0.03 | 0.05 | 0.09 | 0.17 | 0.44 | 1.00 | 2.05 | 6.88 | 12.87 | 28.74 | 47.58 | 70.97 | 106.87 |
| Bolivia (Plurinational State of) | 0.10 | 0.12 | 0.19 | 0.32 | 0.72 | 1.33 | 2.12 | 6.43 | 10.24 | 24.63 | 43.98 | 85.41 | 109.25 |
| Bosnia and Herzegovina | 0.42 | 0.56 | 0.78 | 1.61 | 3.24 | 5.99 | 11.33 | 23.33 | 46.71 | 107.55 | 196.59 | 343.35 | 475.20 |
| Botswana | 0.10 | 0.18 | 0.30 | 0.61 | 1.48 | 2.68 | 4.08 | 19.88 | 30.82 | 68.05 | 103.38 | 176.06 | 232.16 |
| Brazil | 0.03 | 0.06 | 0.10 | 0.18 | 0.48 | 0.84 | 1.37 | 4.32 | 6.87 | 15.70 | 23.92 | 42.30 | 56.59 |
| Brunei Darussalam | 0.01 | 0.02 | 0.03 | 0.06 | 0.15 | 0.29 | 0.61 | 1.72 | 3.66 | 8.46 | 15.16 | 27.88 | 42.06 |
| Bulgaria | 0.18 | 0.34 | 0.67 | 1.53 | 3.60 | 6.87 | 12.45 | 27.84 | 46.27 | 91.03 | 158.97 | 283.44 | 436.00 |
| Burkina Faso | 0.04 | 0.05 | 0.06 | 0.16 | 0.22 | 0.52 | 0.97 | 2.59 | 3.87 | 6.70 | 9.84 | 24.01 | 38.45 |
| Burundi | 0.02 | 0.03 | 0.04 | 0.10 | 0.20 | 0.42 | 0.73 | 2.60 | 4.51 | 10.07 | 16.32 | 30.12 | 34.22 |
| Cabo Verde | 0.26 | 0.24 | 0.29 | 0.69 | 1.14 | 2.56 | 4.32 | 11.44 | 17.14 | 34.18 | 56.67 | 138.28 | 208.94 |
| Cambodia | 0.03 | 0.04 | 0.10 | 0.19 | 0.50 | 1.11 | 1.91 | 5.59 | 9.18 | 24.54 | 39.34 | 77.23 | 101.02 |
| Cameroon | 0.23 | 0.28 | 0.32 | 0.76 | 1.06 | 2.41 | 4.01 | 11.37 | 16.41 | 30.72 | 48.85 | 111.26 | 146.54 |
| Canada | 0.00 | 0.01 | 0.01 | 0.02 | 0.03 | 0.05 | 0.09 | 0.27 | 0.49 | 1.46 | 2.92 | 7.41 | 17.25 |
| Central African Republic | 0.04 | 0.05 | 0.10 | 0.25 | 0.57 | 1.13 | 1.94 | 5.78 | 9.17 | 18.75 | 27.76 | 47.71 | 52.70 |
| Chad | 0.08 | 0.09 | 0.10 | 0.23 | 0.32 | 0.80 | 1.32 | 3.89 | 5.66 | 10.23 | 15.02 | 35.05 | 47.61 |
| Chile | 0.04 | 0.06 | 0.10 | 0.19 | 0.39 | 0.67 | 1.23 | 4.27 | 8.10 | 20.08 | 36.59 | 70.57 | 123.29 |
| China | 0.15 | 0.33 | 0.65 | 1.42 | 2.50 | 5.35 | 10.44 | 21.94 | 41.55 | 86.91 | 145.82 | 255.50 | 381.95 |
| Colombia | 0.05 | 0.06 | 0.10 | 0.15 | 0.28 | 0.47 | 0.77 | 2.71 | 4.46 | 11.67 | 20.06 | 36.92 | 55.29 |
| Comoros | 0.03 | 0.05 | 0.07 | 0.16 | 0.29 | 0.58 | 1.04 | 3.46 | 6.13 | 14.41 | 23.36 | 40.83 | 45.77 |
| Congo | 0.12 | 0.19 | 0.32 | 0.77 | 1.54 | 3.06 | 5.51 | 16.58 | 28.59 | 62.89 | 98.97 | 167.53 | 210.43 |
| Cook Islands | 0.04 | 0.08 | 0.14 | 0.14 | 0.25 | 0.42 | 0.63 | 1.50 | 2.03 | 4.00 | 6.94 | 12.50 | 18.67 |
| Costa Rica | 0.06 | 0.06 | 0.10 | 0.11 | 0.21 | 0.34 | 0.55 | 1.75 | 2.99 | 8.68 | 16.53 | 34.65 | 59.49 |
| Croatia | 0.01 | 0.03 | 0.06 | 0.12 | 0.41 | 0.89 | 1.84 | 6.78 | 13.51 | 34.41 | 67.04 | 134.95 | 200.97 |
| Cuba | 0.04 | 0.10 | 0.16 | 0.35 | 0.73 | 1.61 | 3.21 | 6.97 | 11.96 | 23.56 | 39.19 | 68.29 | 97.07 |
| Cyprus | 0.01 | 0.01 | 0.03 | 0.06 | 0.13 | 0.19 | 0.33 | 1.09 | 2.12 | 7.03 | 17.19 | 58.05 | 118.24 |
| Czechia | 0.02 | 0.04 | 0.08 | 0.15 | 0.39 | 0.96 | 1.96 | 4.98 | 9.29 | 19.27 | 35.54 | 75.07 | 142.35 |
| Côte d'Ivoire | 0.19 | 0.21 | 0.23 | 0.53 | 0.72 | 1.62 | 2.77 | 8.02 | 11.55 | 20.83 | 33.67 | 76.37 | 102.07 |
| Democratic People's Republic of Korea | 0.18 | 0.31 | 0.57 | 1.37 | 3.08 | 6.50 | 12.64 | 24.43 | 40.07 | 66.34 | 94.12 | 127.81 | 158.05 |
| Democratic Republic of the Congo | 0.03 | 0.04 | 0.07 | 0.17 | 0.37 | 0.78 | 1.43 | 4.72 | 8.03 | 17.97 | 27.69 | 51.02 | 59.28 |
| Denmark | 0.01 | 0.01 | 0.02 | 0.05 | 0.11 | 0.21 | 0.44 | 1.18 | 1.88 | 4.97 | 9.18 | 21.91 | 43.42 |
| Djibouti | 0.13 | 0.21 | 0.34 | 0.78 | 1.54 | 3.15 | 5.53 | 17.72 | 30.52 | 67.45 | 107.69 | 180.74 | 194.11 |
| Dominica | 0.07 | 0.13 | 0.21 | 0.41 | 0.74 | 1.41 | 2.65 | 6.90 | 12.61 | 30.18 | 50.31 | 96.59 | 136.28 |
| Dominican Republic | 0.18 | 0.32 | 0.47 | 0.87 | 1.45 | 2.82 | 4.09 | 10.19 | 13.89 | 33.47 | 40.82 | 76.11 | 115.41 |
| Ecuador | 0.16 | 0.19 | 0.24 | 0.35 | 0.68 | 1.14 | 1.78 | 4.38 | 6.39 | 13.26 | 24.00 | 49.23 | 93.97 |
| Egypt | 1.82 | 2.04 | 2.68 | 3.84 | 7.05 | 15.12 | 24.88 | 52.83 | 82.11 | 128.19 | 207.18 | 288.34 | 326.13 |
| El Salvador | 0.08 | 0.10 | 0.13 | 0.19 | 0.38 | 0.64 | 1.01 | 3.14 | 4.89 | 12.11 | 20.86 | 40.69 | 58.44 |
| Equatorial Guinea | 0.09 | 0.14 | 0.24 | 0.57 | 1.24 | 2.43 | 4.46 | 14.62 | 27.28 | 64.97 | 111.56 | 204.52 | 248.90 |
| Eritrea | 0.05 | 0.08 | 0.15 | 0.33 | 0.68 | 1.31 | 2.25 | 7.24 | 11.91 | 27.18 | 43.52 | 74.78 | 85.33 |
| Estonia | 0.00 | 0.01 | 0.01 | 0.03 | 0.11 | 0.27 | 0.62 | 1.49 | 2.74 | 4.45 | 7.37 | 11.55 | 18.55 |
| Eswatini | 0.06 | 0.11 | 0.20 | 0.41 | 0.96 | 1.89 | 3.04 | 14.99 | 21.30 | 47.33 | 75.24 | 135.89 | 180.86 |
| Ethiopia | 0.01 | 0.02 | 0.03 | 0.06 | 0.13 | 0.28 | 0.52 | 1.91 | 3.59 | 8.98 | 15.75 | 31.13 | 38.08 |
| Fiji | 0.13 | 0.20 | 0.42 | 0.44 | 1.00 | 1.88 | 2.68 | 7.03 | 9.48 | 18.83 | 29.70 | 49.36 | 70.01 |
| Finland | 0.01 | 0.01 | 0.01 | 0.02 | 0.04 | 0.07 | 0.15 | 0.39 | 0.74 | 1.51 | 3.26 | 7.16 | 15.11 |
| France | 0.01 | 0.02 | 0.03 | 0.06 | 0.13 | 0.22 | 0.38 | 0.90 | 1.46 | 3.32 | 6.27 | 15.53 | 39.15 |
| Gabon | 0.11 | 0.17 | 0.30 | 0.75 | 1.63 | 3.24 | 5.93 | 18.76 | 32.92 | 69.58 | 113.90 | 197.36 | 241.23 |
| Gambia | 0.15 | 0.17 | 0.21 | 0.49 | 0.70 | 1.65 | 2.81 | 7.96 | 11.65 | 20.54 | 32.75 | 70.34 | 105.81 |
| Georgia | 0.11 | 0.21 | 0.33 | 0.57 | 1.53 | 2.85 | 4.79 | 13.03 | 21.75 | 55.59 | 94.54 | 167.04 | 155.06 |
| Germany | 0.02 | 0.03 | 0.06 | 0.10 | 0.22 | 0.41 | 0.72 | 1.63 | 2.79 | 6.34 | 11.46 | 26.30 | 52.83 |
| Ghana | 0.40 | 0.60 | 0.73 | 1.77 | 2.39 | 5.44 | 8.29 | 23.78 | 34.47 | 60.75 | 100.77 | 203.06 | 276.24 |
| Greece | 0.02 | 0.03 | 0.03 | 0.06 | 0.13 | 0.22 | 0.41 | 1.55 | 3.16 | 10.18 | 23.37 | 66.19 | 135.61 |
| Greenland | 0.00 | 0.01 | 0.03 | 0.06 | 0.13 | 0.21 | 0.41 | 1.20 | 2.06 | 5.27 | 8.62 | 18.75 | 33.26 |
| Grenada | 0.10 | 0.24 | 0.40 | 0.70 | 1.34 | 2.67 | 5.16 | 13.08 | 23.69 | 47.73 | 74.29 | 141.82 | 197.52 |
| Guam | 0.06 | 0.14 | 0.30 | 0.25 | 0.48 | 0.94 | 1.21 | 2.34 | 3.19 | 5.39 | 8.82 | 15.34 | 21.09 |
| Guatemala | 0.08 | 0.08 | 0.11 | 0.14 | 0.28 | 0.44 | 0.66 | 2.53 | 4.20 | 10.42 | 16.55 | 42.51 | 85.84 |
| Guinea | 0.09 | 0.11 | 0.12 | 0.30 | 0.41 | 0.99 | 1.63 | 4.80 | 6.77 | 12.34 | 18.88 | 43.54 | 59.14 |
| Guinea-Bissau | 0.16 | 0.19 | 0.22 | 0.55 | 0.76 | 1.66 | 2.82 | 7.54 | 10.35 | 17.83 | 26.31 | 56.24 | 77.33 |
| Guyana | 0.32 | 0.52 | 0.72 | 1.28 | 2.43 | 4.51 | 7.80 | 21.09 | 34.55 | 78.07 | 103.60 | 172.00 | 196.04 |
| Haiti | 0.08 | 0.13 | 0.20 | 0.41 | 0.71 | 1.40 | 2.31 | 5.93 | 9.44 | 22.89 | 34.50 | 64.35 | 78.77 |
| Honduras | 0.09 | 0.12 | 0.21 | 0.40 | 0.79 | 1.46 | 2.53 | 7.62 | 12.92 | 28.91 | 46.98 | 82.53 | 109.09 |
| Hungary | 0.05 | 0.11 | 0.20 | 0.45 | 1.05 | 2.65 | 4.99 | 9.96 | 15.51 | 27.55 | 48.44 | 83.61 | 130.54 |
| Iceland | 0.00 | 0.00 | 0.00 | 0.01 | 0.02 | 0.03 | 0.05 | 0.16 | 0.30 | 0.87 | 1.74 | 4.31 | 9.38 |
| India | 0.08 | 0.14 | 0.25 | 0.44 | 1.02 | 2.38 | 4.76 | 13.57 | 22.67 | 44.40 | 64.28 | 98.45 | 131.47 |
| Indonesia | 0.15 | 0.21 | 0.44 | 0.79 | 1.87 | 3.82 | 6.69 | 18.69 | 31.05 | 70.03 | 103.88 | 192.69 | 257.64 |
| Iran (Islamic Republic of) | 0.48 | 0.66 | 1.06 | 1.79 | 3.11 | 6.12 | 10.79 | 21.38 | 35.59 | 63.60 | 127.25 | 200.22 | 299.92 |
| Iraq | 0.42 | 0.74 | 1.46 | 2.92 | 7.24 | 14.70 | 24.99 | 58.30 | 96.11 | 173.13 | 274.06 | 420.55 | 561.32 |
| Ireland | 0.00 | 0.01 | 0.01 | 0.02 | 0.04 | 0.06 | 0.12 | 0.37 | 0.74 | 2.32 | 5.25 | 13.26 | 29.55 |
| Israel | 0.02 | 0.03 | 0.03 | 0.06 | 0.15 | 0.30 | 0.53 | 1.50 | 2.54 | 6.89 | 13.76 | 34.27 | 70.01 |
| Italy | 0.01 | 0.02 | 0.03 | 0.05 | 0.11 | 0.18 | 0.33 | 0.97 | 1.86 | 5.65 | 12.88 | 36.39 | 91.75 |
| Jamaica | 0.09 | 0.11 | 0.14 | 0.28 | 0.53 | 0.89 | 1.46 | 4.91 | 8.97 | 31.62 | 49.72 | 95.75 | 122.50 |
| Japan | 0.01 | 0.02 | 0.04 | 0.08 | 0.17 | 0.30 | 0.54 | 1.30 | 2.31 | 4.80 | 8.95 | 19.11 | 50.78 |
| Jordan | 0.11 | 0.22 | 0.41 | 0.77 | 1.80 | 3.95 | 7.55 | 18.64 | 36.88 | 62.24 | 129.39 | 198.69 | 287.71 |
| Kazakhstan | 0.17 | 0.35 | 0.70 | 1.32 | 3.02 | 5.91 | 11.08 | 25.95 | 43.84 | 82.61 | 130.32 | 235.99 | 323.52 |
| Kenya | 0.03 | 0.05 | 0.08 | 0.17 | 0.35 | 0.68 | 1.18 | 4.08 | 7.17 | 16.94 | 28.72 | 53.22 | 60.69 |
| Kiribati | 0.13 | 0.29 | 0.56 | 0.53 | 1.13 | 1.73 | 2.69 | 5.98 | 7.51 | 14.25 | 21.53 | 33.11 | 37.60 |
| Kuwait | 0.21 | 0.37 | 0.62 | 1.07 | 1.96 | 3.68 | 6.28 | 15.48 | 29.73 | 60.74 | 107.97 | 168.03 | 266.73 |
| Kyrgyzstan | 0.12 | 0.24 | 0.53 | 1.04 | 3.10 | 5.86 | 10.87 | 28.13 | 42.84 | 67.73 | 94.88 | 131.07 | 151.73 |
| Lao People's Democratic Republic | 0.06 | 0.09 | 0.18 | 0.35 | 0.78 | 1.64 | 2.77 | 8.14 | 12.71 | 30.59 | 44.45 | 81.07 | 99.51 |
| Latvia | 0.02 | 0.04 | 0.10 | 0.29 | 0.91 | 2.22 | 4.88 | 11.30 | 20.50 | 35.66 | 60.39 | 107.74 | 170.36 |
| Lebanon | 0.21 | 0.30 | 0.52 | 0.83 | 1.31 | 2.23 | 3.84 | 7.60 | 12.67 | 25.42 | 50.77 | 94.12 | 153.04 |
| Lesotho | 0.05 | 0.10 | 0.18 | 0.38 | 0.97 | 1.78 | 2.81 | 13.98 | 19.45 | 45.63 | 68.66 | 127.25 | 168.21 |
| Liberia | 0.10 | 0.12 | 0.13 | 0.30 | 0.42 | 0.97 | 1.62 | 4.68 | 6.84 | 12.63 | 20.73 | 48.38 | 70.54 |
| Libya | 0.91 | 1.24 | 2.02 | 2.97 | 5.16 | 8.08 | 12.72 | 28.25 | 48.33 | 76.68 | 134.66 | 170.49 | 250.62 |
| Lithuania | 0.02 | 0.06 | 0.11 | 0.27 | 0.67 | 1.31 | 2.91 | 7.09 | 12.78 | 21.14 | 34.07 | 57.98 | 94.14 |
| Luxembourg | 0.01 | 0.02 | 0.02 | 0.04 | 0.08 | 0.14 | 0.25 | 0.69 | 1.39 | 3.87 | 8.36 | 19.63 | 37.77 |
| Madagascar | 0.05 | 0.08 | 0.12 | 0.23 | 0.41 | 0.77 | 1.30 | 4.03 | 6.68 | 14.76 | 22.49 | 38.85 | 41.61 |
| Malawi | 0.02 | 0.03 | 0.05 | 0.13 | 0.26 | 0.54 | 0.95 | 3.12 | 5.27 | 11.63 | 19.60 | 33.31 | 38.05 |
| Malaysia | 0.09 | 0.16 | 0.29 | 0.49 | 1.07 | 2.17 | 3.54 | 8.35 | 13.45 | 31.22 | 44.90 | 76.19 | 96.65 |
| Maldives | 0.03 | 0.06 | 0.09 | 0.14 | 0.29 | 0.58 | 0.92 | 2.44 | 4.41 | 10.03 | 16.82 | 39.37 | 51.48 |
| Mali | 0.06 | 0.09 | 0.09 | 0.19 | 0.23 | 0.57 | 0.88 | 2.81 | 4.44 | 9.42 | 15.57 | 38.40 | 56.91 |
| Malta | 0.02 | 0.03 | 0.04 | 0.07 | 0.12 | 0.21 | 0.38 | 1.17 | 2.47 | 6.78 | 14.78 | 35.07 | 60.12 |
| Marshall Islands | 0.14 | 0.26 | 0.52 | 0.52 | 0.99 | 1.55 | 2.19 | 5.31 | 7.26 | 14.57 | 23.01 | 40.27 | 53.23 |
| Mauritania | 0.21 | 0.27 | 0.30 | 0.72 | 0.98 | 2.44 | 3.79 | 10.76 | 16.54 | 30.84 | 51.59 | 112.67 | 160.71 |
| Mauritius | 0.06 | 0.10 | 0.21 | 0.30 | 0.66 | 1.16 | 1.61 | 5.80 | 9.16 | 22.47 | 36.34 | 67.91 | 91.56 |
| Mexico | 0.07 | 0.09 | 0.15 | 0.24 | 0.48 | 0.83 | 1.33 | 3.70 | 6.05 | 12.59 | 21.52 | 40.89 | 65.76 |
| Micronesia (Federated States of) | 0.17 | 0.35 | 0.56 | 0.48 | 1.22 | 2.10 | 3.03 | 7.05 | 9.17 | 18.67 | 29.56 | 50.06 | 65.92 |
| Monaco | 0.03 | 0.05 | 0.07 | 0.11 | 0.20 | 0.33 | 0.57 | 1.45 | 2.68 | 7.09 | 14.44 | 33.18 | 66.79 |
| Mongolia | 0.10 | 0.32 | 0.68 | 1.28 | 2.93 | 4.97 | 6.36 | 14.56 | 20.42 | 34.14 | 38.74 | 60.36 | 92.14 |
| Montenegro | 0.02 | 0.05 | 0.09 | 0.19 | 0.47 | 1.17 | 2.60 | 7.24 | 14.13 | 31.77 | 55.88 | 110.85 | 180.01 |
| Morocco | 0.43 | 0.77 | 1.38 | 2.69 | 5.04 | 9.95 | 17.61 | 37.94 | 64.52 | 116.15 | 194.04 | 295.08 | 403.36 |
| Mozambique | 0.02 | 0.04 | 0.07 | 0.18 | 0.39 | 0.83 | 1.56 | 4.89 | 7.93 | 14.65 | 22.74 | 32.27 | 30.20 |
| Myanmar | 0.23 | 0.26 | 0.44 | 0.74 | 1.55 | 3.36 | 5.42 | 15.82 | 25.48 | 61.67 | 95.91 | 183.59 | 228.70 |
| Namibia | 0.04 | 0.08 | 0.15 | 0.32 | 0.83 | 1.71 | 2.85 | 14.99 | 23.18 | 55.53 | 89.64 | 159.07 | 208.71 |
| Nauru | 0.19 | 0.33 | 0.62 | 0.63 | 1.16 | 1.78 | 2.33 | 5.12 | 6.60 | 12.52 | 19.24 | 30.50 | 38.04 |
| Nepal | 0.03 | 0.06 | 0.11 | 0.25 | 0.64 | 1.65 | 3.38 | 10.84 | 19.13 | 41.46 | 65.47 | 92.62 | 145.43 |
| Netherlands | 0.01 | 0.02 | 0.04 | 0.08 | 0.17 | 0.30 | 0.53 | 1.28 | 2.20 | 5.72 | 11.42 | 27.62 | 59.14 |
| New Zealand | 0.00 | 0.01 | 0.01 | 0.02 | 0.04 | 0.05 | 0.10 | 0.24 | 0.44 | 1.30 | 2.88 | 7.28 | 16.94 |
| Nicaragua | 0.02 | 0.03 | 0.06 | 0.13 | 0.27 | 0.53 | 0.89 | 3.39 | 5.90 | 15.42 | 26.32 | 52.79 | 73.61 |
| Niger | 0.05 | 0.06 | 0.07 | 0.17 | 0.23 | 0.59 | 0.94 | 2.89 | 4.34 | 8.42 | 13.96 | 33.93 | 49.57 |
| Nigeria | 0.13 | 0.16 | 0.20 | 0.48 | 0.69 | 1.69 | 2.88 | 9.33 | 16.01 | 32.93 | 58.54 | 121.47 | 147.91 |
| Niue | 0.07 | 0.12 | 0.22 | 0.24 | 0.50 | 0.90 | 1.36 | 3.29 | 4.57 | 8.89 | 14.28 | 23.21 | 30.81 |
| North Macedonia | 0.18 | 0.29 | 0.52 | 0.92 | 2.53 | 4.95 | 11.51 | 34.11 | 73.40 | 178.16 | 397.85 | 713.19 | 1062.05 |
| Northern Mariana Islands | 0.04 | 0.09 | 0.19 | 0.20 | 0.46 | 0.78 | 1.29 | 3.05 | 4.50 | 8.71 | 14.98 | 25.97 | 37.57 |
| Norway | 0.00 | 0.00 | 0.01 | 0.01 | 0.03 | 0.05 | 0.09 | 0.31 | 0.55 | 1.63 | 3.14 | 8.06 | 17.67 |
| Oman | 0.28 | 0.38 | 0.67 | 1.05 | 2.22 | 4.57 | 9.81 | 25.86 | 55.48 | 112.38 | 213.42 | 419.51 | 414.95 |
| Pakistan | 0.17 | 0.31 | 0.44 | 0.80 | 1.60 | 3.34 | 6.10 | 18.53 | 30.95 | 65.55 | 93.63 | 129.20 | 183.62 |
| Palau | 0.11 | 0.24 | 0.45 | 0.39 | 0.76 | 1.35 | 1.99 | 4.38 | 5.97 | 10.10 | 13.62 | 19.54 | 29.58 |
| Palestine | 0.27 | 0.33 | 0.58 | 1.23 | 2.63 | 6.54 | 13.47 | 29.86 | 60.74 | 119.16 | 209.00 | 313.28 | 448.44 |
| Panama | 0.03 | 0.04 | 0.06 | 0.13 | 0.17 | 0.31 | 0.50 | 1.76 | 2.99 | 9.10 | 16.83 | 37.80 | 60.74 |
| Papua New Guinea | 0.03 | 0.05 | 0.12 | 0.12 | 0.28 | 0.50 | 0.77 | 1.99 | 2.79 | 5.86 | 9.26 | 16.92 | 21.49 |
| Paraguay | 0.03 | 0.05 | 0.07 | 0.14 | 0.42 | 0.77 | 1.30 | 4.33 | 6.88 | 16.94 | 26.32 | 49.49 | 64.42 |
| Peru | 0.12 | 0.13 | 0.16 | 0.24 | 0.42 | 0.73 | 1.12 | 3.19 | 5.18 | 11.45 | 21.25 | 39.91 | 73.91 |
| Philippines | 0.11 | 0.18 | 0.33 | 0.56 | 1.25 | 2.25 | 3.54 | 8.83 | 13.42 | 27.62 | 38.70 | 69.66 | 85.03 |
| Poland | 0.05 | 0.11 | 0.21 | 0.47 | 1.18 | 2.36 | 4.44 | 10.21 | 17.19 | 32.74 | 56.09 | 104.47 | 183.57 |
| Portugal | 0.00 | 0.01 | 0.02 | 0.04 | 0.08 | 0.15 | 0.28 | 0.96 | 1.83 | 5.51 | 11.37 | 27.78 | 54.27 |
| Puerto Rico | 0.01 | 0.01 | 0.02 | 0.02 | 0.03 | 0.07 | 0.10 | 0.33 | 0.52 | 1.54 | 2.68 | 6.26 | 11.34 |
| Qatar | 0.10 | 0.15 | 0.33 | 0.42 | 0.96 | 1.54 | 2.53 | 6.24 | 13.70 | 35.60 | 99.53 | 260.52 | 404.30 |
| Republic of Korea | 0.03 | 0.05 | 0.09 | 0.18 | 0.43 | 0.85 | 1.63 | 4.08 | 7.67 | 20.77 | 41.36 | 87.49 | 163.35 |
| Republic of Moldova | 0.07 | 0.11 | 0.22 | 0.46 | 1.14 | 2.46 | 5.31 | 13.69 | 25.48 | 44.64 | 58.85 | 86.48 | 99.55 |
| Romania | 0.07 | 0.12 | 0.22 | 0.44 | 1.24 | 2.38 | 4.45 | 12.72 | 22.19 | 52.32 | 91.85 | 165.24 | 239.84 |
| Russian Federation | 0.09 | 0.19 | 0.39 | 0.74 | 1.65 | 3.31 | 6.28 | 14.35 | 24.32 | 47.15 | 77.23 | 123.25 | 185.71 |
| Rwanda | 0.02 | 0.03 | 0.05 | 0.12 | 0.25 | 0.54 | 1.08 | 3.69 | 6.98 | 16.70 | 29.13 | 55.76 | 64.83 |
| Saint Kitts and Nevis | 0.04 | 0.06 | 0.11 | 0.26 | 0.58 | 1.34 | 2.44 | 5.65 | 9.61 | 20.82 | 33.21 | 65.50 | 95.69 |
| Saint Lucia | 0.09 | 0.18 | 0.28 | 0.61 | 1.15 | 2.08 | 3.73 | 9.21 | 14.08 | 36.03 | 56.22 | 114.86 | 200.28 |
| Saint Vincent and the Grenadines | 0.14 | 0.24 | 0.35 | 0.70 | 1.39 | 2.38 | 3.79 | 8.86 | 14.02 | 37.85 | 60.35 | 125.13 | 174.92 |
| Samoa | 0.08 | 0.16 | 0.32 | 0.35 | 0.76 | 1.33 | 1.87 | 4.66 | 6.69 | 13.50 | 22.23 | 38.17 | 53.55 |
| San Marino | 0.02 | 0.02 | 0.04 | 0.06 | 0.10 | 0.18 | 0.34 | 0.99 | 1.94 | 5.26 | 10.91 | 25.05 | 48.02 |
| Sao Tome and Principe | 0.28 | 0.29 | 0.35 | 0.74 | 1.03 | 2.28 | 3.72 | 9.97 | 15.98 | 29.98 | 47.37 | 95.40 | 133.66 |
| Saudi Arabia | 0.54 | 0.95 | 2.00 | 3.90 | 7.00 | 12.75 | 20.18 | 42.35 | 66.69 | 115.76 | 191.21 | 318.77 | 516.98 |
| Senegal | 0.14 | 0.15 | 0.17 | 0.42 | 0.60 | 1.40 | 2.32 | 6.73 | 10.06 | 18.25 | 29.51 | 66.82 | 97.37 |
| Serbia | 0.09 | 0.21 | 0.36 | 0.79 | 2.10 | 4.79 | 10.25 | 23.69 | 47.63 | 92.06 | 185.35 | 426.33 | 669.47 |
| Seychelles | 0.13 | 0.21 | 0.32 | 0.56 | 1.13 | 1.92 | 2.80 | 7.09 | 10.94 | 21.02 | 39.61 | 71.57 | 98.69 |
| Sierra Leone | 0.13 | 0.16 | 0.17 | 0.40 | 0.52 | 1.25 | 1.93 | 5.54 | 8.12 | 14.32 | 22.58 | 50.59 | 69.30 |
| Singapore | 0.01 | 0.01 | 0.02 | 0.05 | 0.12 | 0.24 | 0.48 | 1.43 | 2.72 | 7.34 | 13.10 | 28.69 | 52.35 |
| Slovakia | 0.06 | 0.13 | 0.25 | 0.56 | 1.14 | 2.15 | 4.25 | 10.40 | 18.34 | 34.16 | 58.32 | 103.32 | 144.20 |
| Slovenia | 0.01 | 0.01 | 0.04 | 0.07 | 0.19 | 0.50 | 1.00 | 2.89 | 6.00 | 13.87 | 26.55 | 61.23 | 96.83 |
| Solomon Islands | 0.05 | 0.12 | 0.31 | 0.34 | 0.82 | 1.44 | 2.05 | 4.73 | 6.21 | 12.87 | 20.05 | 33.93 | 42.25 |
| Somalia | 0.01 | 0.01 | 0.02 | 0.05 | 0.10 | 0.20 | 0.34 | 1.15 | 1.90 | 4.15 | 6.56 | 11.70 | 13.95 |
| South Africa | 0.13 | 0.25 | 0.31 | 0.44 | 0.87 | 1.73 | 3.05 | 14.92 | 22.56 | 45.09 | 67.65 | 141.79 | 207.64 |
| South Sudan | 0.02 | 0.03 | 0.06 | 0.12 | 0.25 | 0.53 | 0.96 | 3.47 | 6.39 | 14.87 | 25.27 | 45.78 | 50.35 |
| Spain | 0.01 | 0.01 | 0.02 | 0.03 | 0.07 | 0.13 | 0.22 | 0.58 | 0.97 | 2.67 | 5.56 | 15.25 | 36.70 |
| Sri Lanka | 0.10 | 0.15 | 0.24 | 0.38 | 0.80 | 1.53 | 2.39 | 6.40 | 11.63 | 26.95 | 46.38 | 91.43 | 156.12 |
| Sudan | 0.65 | 1.06 | 1.67 | 2.95 | 5.22 | 10.23 | 16.75 | 35.75 | 58.37 | 104.77 | 176.58 | 273.11 | 349.25 |
| Suriname | 0.16 | 0.28 | 0.47 | 0.74 | 1.35 | 2.62 | 4.45 | 11.51 | 19.53 | 41.51 | 65.88 | 114.54 | 130.76 |
| Sweden | 0.00 | 0.00 | 0.01 | 0.01 | 0.03 | 0.05 | 0.09 | 0.23 | 0.46 | 1.15 | 2.52 | 6.84 | 14.20 |
| Switzerland | 0.01 | 0.01 | 0.01 | 0.03 | 0.05 | 0.09 | 0.17 | 0.51 | 0.93 | 2.36 | 5.14 | 13.84 | 32.94 |
| Syrian Arab Republic | 0.59 | 0.60 | 1.00 | 1.54 | 3.02 | 6.10 | 9.97 | 23.87 | 36.66 | 64.38 | 103.23 | 185.37 | 299.18 |
| Taiwan (Province of China) | 0.03 | 0.06 | 0.11 | 0.20 | 0.45 | 0.75 | 1.26 | 3.18 | 5.17 | 12.76 | 22.24 | 49.52 | 74.72 |
| Tajikistan | 0.04 | 0.08 | 0.11 | 0.21 | 0.56 | 1.28 | 2.75 | 10.39 | 20.03 | 56.50 | 85.09 | 126.07 | 125.08 |
| Thailand | 0.17 | 0.30 | 0.50 | 0.70 | 1.28 | 2.18 | 3.09 | 6.68 | 10.27 | 20.07 | 32.62 | 58.86 | 79.98 |
| Timor-Leste | 0.05 | 0.08 | 0.14 | 0.26 | 0.65 | 1.40 | 2.43 | 6.71 | 10.72 | 25.19 | 39.46 | 72.67 | 92.18 |
| Togo | 0.16 | 0.19 | 0.21 | 0.52 | 0.74 | 1.72 | 2.93 | 7.82 | 11.10 | 18.56 | 30.12 | 65.03 | 84.61 |
| Tokelau | 0.07 | 0.12 | 0.19 | 0.26 | 0.41 | 0.72 | 0.96 | 2.27 | 3.25 | 6.29 | 11.12 | 17.82 | 25.73 |
| Tonga | 0.05 | 0.09 | 0.17 | 0.20 | 0.42 | 0.76 | 1.24 | 3.32 | 5.48 | 11.41 | 18.27 | 31.16 | 44.07 |
| Trinidad and Tobago | 0.17 | 0.34 | 0.48 | 0.77 | 1.19 | 2.04 | 3.32 | 9.11 | 15.85 | 37.72 | 61.71 | 108.53 | 144.64 |
| Tunisia | 0.31 | 0.58 | 0.95 | 1.62 | 2.76 | 5.06 | 9.72 | 20.37 | 36.56 | 67.83 | 121.98 | 214.18 | 297.07 |
| Turkey | 0.08 | 0.13 | 0.25 | 0.44 | 0.98 | 1.90 | 3.21 | 8.31 | 15.59 | 36.13 | 68.11 | 118.75 | 174.19 |
| Turkmenistan | 0.59 | 0.76 | 1.39 | 2.29 | 6.02 | 10.57 | 18.50 | 48.83 | 67.28 | 129.96 | 140.32 | 192.16 | 181.89 |
| Tuvalu | 0.09 | 0.18 | 0.36 | 0.35 | 0.68 | 1.09 | 1.47 | 3.43 | 4.66 | 9.10 | 14.64 | 24.72 | 33.11 |
| Uganda | 0.03 | 0.04 | 0.06 | 0.15 | 0.29 | 0.64 | 1.10 | 3.79 | 6.73 | 15.68 | 27.58 | 50.62 | 55.66 |
| Ukraine | 0.21 | 0.39 | 0.77 | 1.40 | 2.79 | 5.38 | 9.82 | 20.26 | 33.90 | 57.28 | 86.97 | 126.13 | 195.05 |
| United Arab Emirates | 0.45 | 0.88 | 2.09 | 3.15 | 6.77 | 11.66 | 18.72 | 39.35 | 61.41 | 88.50 | 186.20 | 302.53 | 396.20 |
| United Kingdom | 0.01 | 0.02 | 0.03 | 0.05 | 0.10 | 0.17 | 0.28 | 0.74 | 1.28 | 3.63 | 7.56 | 19.44 | 45.02 |
| United Republic of Tanzania | 0.03 | 0.05 | 0.08 | 0.16 | 0.32 | 0.66 | 1.18 | 4.48 | 8.36 | 18.27 | 29.90 | 49.85 | 48.33 |
| United States of America | 0.01 | 0.02 | 0.03 | 0.04 | 0.08 | 0.13 | 0.21 | 0.59 | 0.88 | 2.36 | 4.25 | 10.55 | 21.15 |
| United States Virgin Islands | 0.01 | 0.03 | 0.06 | 0.13 | 0.28 | 0.59 | 1.02 | 2.40 | 3.84 | 8.62 | 13.33 | 26.88 | 39.00 |
| Uruguay | 0.03 | 0.04 | 0.07 | 0.14 | 0.26 | 0.53 | 1.01 | 2.68 | 4.69 | 9.99 | 16.57 | 33.45 | 56.00 |
| Uzbekistan | 0.29 | 0.54 | 1.00 | 2.08 | 5.54 | 11.21 | 18.97 | 51.72 | 70.53 | 137.78 | 192.75 | 306.98 | 346.83 |
| Vanuatu | 0.11 | 0.23 | 0.48 | 0.48 | 1.03 | 1.68 | 2.38 | 5.79 | 7.99 | 15.41 | 23.99 | 40.57 | 55.29 |
| Venezuela(BolivarianRepublic of) | 0.06 | 0.06 | 0.09 | 0.16 | 0.34 | 0.69 | 1.28 | 4.67 | 7.94 | 22.56 | 39.11 | 75.46 | 90.79 |
| Viet Nam | 0.08 | 0.15 | 0.33 | 0.67 | 1.71 | 3.53 | 6.39 | 16.28 | 25.94 | 62.34 | 98.59 | 180.89 | 224.22 |
| Yemen | 0.39 | 0.69 | 1.34 | 2.63 | 4.70 | 9.12 | 14.82 | 32.39 | 51.78 | 90.39 | 147.03 | 226.35 | 290.83 |
| Zambia | 0.05 | 0.08 | 0.14 | 0.33 | 0.71 | 1.43 | 2.71 | 8.83 | 15.14 | 34.25 | 54.72 | 98.91 | 107.95 |
| Zimbabwe | 0.06 | 0.11 | 0.18 | 0.31 | 0.69 | 1.47 | 2.29 | 8.33 | 11.03 | 22.67 | 38.14 | 65.55 | 92.61 |

**Supplementary Table 6 DALY Rate of Ischaemic Stroke Attributed to Ambient Particulate Matter Pollution by Age Group in Countries Around the World in 2019**

| **Country**  **Age** | **25-29 years** | **30-34 years** | **35-39 years** | **40-44 years** | **45-49 years** | **50-54 years** | **55-59 years** | **60-64 years** | **65-69 years** | **70-74 years** | **75-79 years** | **80-84 years** | **85+ years** |
| --- | --- | --- | --- | --- | --- | --- | --- | --- | --- | --- | --- | --- | --- |
| Afghanistan | 24.83 | 40.39 | 66.51 | 110.16 | 172.84 | 275.20 | 358.10 | 603.39 | 771.76 | 1057.30 | 1349.37 | 1556.90 | 1469.27 |
| Albania | 18.68 | 23.89 | 25.90 | 31.06 | 44.05 | 62.29 | 94.06 | 193.99 | 277.34 | 507.84 | 643.20 | 929.21 | 793.34 |
| Algeria | 42.10 | 62.22 | 94.21 | 140.49 | 204.59 | 321.33 | 466.00 | 813.12 | 1101.76 | 1524.82 | 2266.45 | 2979.42 | 5045.61 |
| American Samoa | 8.03 | 11.40 | 16.56 | 18.39 | 28.04 | 40.80 | 52.82 | 97.11 | 121.08 | 189.03 | 242.19 | 297.74 | 281.80 |
| Andorra | 3.14 | 3.72 | 4.33 | 5.41 | 7.39 | 10.09 | 14.53 | 26.18 | 38.50 | 75.60 | 115.87 | 191.79 | 221.64 |
| Angola | 11.42 | 14.42 | 19.18 | 32.46 | 54.41 | 93.85 | 140.50 | 335.60 | 464.23 | 800.84 | 970.85 | 1267.48 | 992.70 |
| Antigua and Barbuda | 9.52 | 12.09 | 16.94 | 24.00 | 37.47 | 62.29 | 100.15 | 202.13 | 265.51 | 476.52 | 644.57 | 992.04 | 1044.46 |
| Argentina | 5.59 | 6.77 | 8.70 | 12.24 | 19.57 | 29.22 | 42.79 | 105.72 | 146.86 | 263.76 | 337.36 | 476.17 | 461.04 |
| Armenia | 24.08 | 32.14 | 47.89 | 74.09 | 130.30 | 205.35 | 300.10 | 517.45 | 753.31 | 1258.09 | 1542.30 | 2044.79 | 1828.01 |
| Australia | 2.19 | 2.66 | 3.18 | 3.90 | 5.16 | 6.47 | 9.06 | 13.87 | 19.34 | 35.06 | 56.24 | 104.60 | 160.18 |
| Austria | 5.05 | 5.87 | 7.05 | 9.28 | 12.58 | 18.23 | 28.72 | 49.07 | 73.52 | 127.23 | 179.97 | 309.15 | 393.93 |
| Azerbaijan | 16.33 | 25.50 | 37.19 | 57.74 | 104.43 | 179.28 | 273.30 | 567.99 | 829.85 | 1381.46 | 1640.73 | 2251.17 | 2379.75 |
| Bahamas | 10.86 | 16.42 | 23.28 | 33.26 | 45.79 | 70.17 | 93.08 | 160.42 | 219.93 | 381.21 | 514.14 | 717.72 | 643.57 |
| Bahrain | 32.38 | 38.52 | 55.20 | 70.18 | 98.18 | 146.29 | 212.84 | 398.36 | 654.82 | 1217.39 | 2031.18 | 2702.77 | 3042.54 |
| Bangladesh | 14.31 | 19.85 | 25.28 | 35.74 | 62.55 | 121.21 | 165.16 | 446.34 | 635.50 | 1061.39 | 1209.23 | 2008.37 | 2213.12 |
| Barbados | 12.66 | 19.02 | 25.03 | 37.87 | 58.51 | 83.61 | 126.59 | 237.80 | 338.34 | 675.42 | 863.63 | 1213.99 | 1376.95 |
| Belarus | 13.89 | 21.33 | 32.74 | 57.81 | 119.34 | 198.68 | 331.52 | 613.82 | 874.12 | 1209.57 | 1381.93 | 1683.19 | 1498.65 |
| Belgium | 5.23 | 6.58 | 7.77 | 10.30 | 14.50 | 20.31 | 30.28 | 56.10 | 78.68 | 146.40 | 215.47 | 363.11 | 472.62 |
| Belize | 11.63 | 17.33 | 20.06 | 29.19 | 44.89 | 67.35 | 103.30 | 186.63 | 233.18 | 402.58 | 511.36 | 732.79 | 704.13 |
| Benin | 9.59 | 11.25 | 13.08 | 21.85 | 28.99 | 53.27 | 81.48 | 186.87 | 249.83 | 371.49 | 469.92 | 759.68 | 620.62 |
| Bermuda | 2.39 | 3.06 | 4.06 | 5.96 | 8.79 | 13.79 | 19.39 | 32.62 | 43.63 | 73.91 | 99.13 | 158.64 | 169.00 |
| Bhutan | 8.57 | 10.68 | 13.80 | 19.42 | 32.66 | 56.26 | 92.81 | 226.65 | 344.89 | 601.75 | 776.76 | 878.40 | 899.32 |
| Bolivia (Plurinational State of) | 11.87 | 14.14 | 18.74 | 26.05 | 44.03 | 67.04 | 91.94 | 208.95 | 278.25 | 520.42 | 724.80 | 1055.93 | 936.10 |
| Bosnia and Herzegovina | 43.48 | 55.49 | 72.33 | 118.79 | 195.08 | 307.23 | 494.55 | 831.34 | 1339.77 | 2366.47 | 3327.50 | 4322.41 | 4109.72 |
| Botswana | 17.76 | 23.81 | 32.05 | 48.86 | 89.64 | 137.77 | 187.01 | 632.42 | 840.89 | 1470.89 | 1774.06 | 2267.45 | 2075.26 |
| Brazil | 5.98 | 7.71 | 10.67 | 15.39 | 28.97 | 42.80 | 61.33 | 143.46 | 190.89 | 338.26 | 407.38 | 539.24 | 480.98 |
| Brunei Darussalam | 3.54 | 4.44 | 6.31 | 9.45 | 16.02 | 25.16 | 41.24 | 78.25 | 126.33 | 214.16 | 293.82 | 395.74 | 418.11 |
| Bulgaria | 23.66 | 35.84 | 56.21 | 101.41 | 194.03 | 320.48 | 506.68 | 922.28 | 1277.68 | 1973.15 | 2659.99 | 3538.06 | 3767.30 |
| Burkina Faso | 5.32 | 6.03 | 6.91 | 12.12 | 15.50 | 27.48 | 43.02 | 87.81 | 110.65 | 153.09 | 178.75 | 311.80 | 330.64 |
| Burundi | 3.76 | 4.55 | 5.65 | 8.74 | 13.80 | 22.62 | 33.75 | 86.52 | 125.30 | 219.68 | 280.21 | 386.76 | 309.22 |
| Cabo Verde | 32.74 | 33.46 | 38.98 | 62.53 | 86.81 | 146.79 | 212.09 | 414.14 | 522.08 | 803.31 | 1036.55 | 1800.26 | 1802.22 |
| Cambodia | 6.04 | 7.98 | 11.98 | 17.32 | 32.33 | 57.87 | 89.18 | 198.10 | 276.94 | 551.73 | 689.94 | 987.96 | 885.12 |
| Cameroon | 24.27 | 27.94 | 31.75 | 54.96 | 70.35 | 125.01 | 181.62 | 388.84 | 476.66 | 701.69 | 874.05 | 1435.10 | 1290.71 |
| Canada | 3.12 | 3.70 | 4.28 | 5.18 | 6.84 | 9.27 | 13.04 | 21.06 | 29.96 | 52.44 | 75.74 | 123.34 | 162.17 |
| Central African Republic | 4.82 | 6.24 | 8.80 | 16.60 | 30.79 | 52.13 | 78.07 | 182.61 | 244.14 | 397.38 | 466.13 | 602.95 | 473.97 |
| Chad | 7.69 | 8.55 | 9.58 | 16.36 | 21.24 | 40.36 | 58.41 | 130.17 | 160.08 | 229.87 | 268.21 | 452.18 | 420.34 |
| Chile | 9.84 | 11.89 | 16.17 | 23.23 | 35.55 | 51.72 | 76.94 | 167.24 | 251.81 | 464.28 | 652.77 | 926.11 | 1044.47 |
| China | 29.57 | 43.17 | 62.75 | 102.57 | 154.40 | 274.98 | 471.72 | 829.74 | 1306.67 | 2124.39 | 2767.99 | 3563.82 | 3654.47 |
| Colombia | 9.71 | 11.29 | 14.87 | 18.67 | 25.95 | 34.85 | 47.71 | 104.17 | 140.87 | 269.53 | 360.70 | 492.19 | 469.69 |
| Comoros | 5.88 | 7.33 | 9.35 | 14.22 | 21.68 | 34.68 | 52.34 | 122.72 | 179.55 | 323.19 | 408.99 | 532.38 | 415.11 |
| Congo | 19.03 | 24.26 | 33.22 | 57.25 | 94.56 | 156.61 | 243.68 | 555.32 | 800.91 | 1376.53 | 1704.58 | 2160.87 | 1910.65 |
| Cook Islands | 6.74 | 9.93 | 13.99 | 14.94 | 21.08 | 29.44 | 39.63 | 68.27 | 82.92 | 121.21 | 156.04 | 199.06 | 192.77 |
| Costa Rica | 9.86 | 10.15 | 13.76 | 15.46 | 21.19 | 28.00 | 37.65 | 73.19 | 101.38 | 206.20 | 299.81 | 457.74 | 493.28 |
| Croatia | 10.41 | 13.60 | 18.01 | 24.70 | 41.75 | 66.29 | 109.43 | 260.47 | 420.41 | 798.16 | 1186.43 | 1746.77 | 1756.50 |
| Cuba | 8.63 | 13.33 | 18.12 | 28.90 | 46.67 | 80.36 | 131.96 | 229.83 | 324.43 | 504.26 | 653.57 | 853.03 | 782.30 |
| Cyprus | 6.18 | 7.08 | 8.68 | 11.19 | 15.42 | 19.82 | 27.20 | 51.50 | 77.92 | 174.88 | 318.67 | 748.12 | 1044.21 |
| Czechia | 11.17 | 14.87 | 19.95 | 27.44 | 41.86 | 69.47 | 112.86 | 207.26 | 311.76 | 488.45 | 679.36 | 1019.91 | 1214.51 |
| Côte d'Ivoire | 19.13 | 21.01 | 23.12 | 39.31 | 49.89 | 87.56 | 129.84 | 278.92 | 340.70 | 484.75 | 611.70 | 996.10 | 902.60 |
| Democratic People's Republic of Korea | 22.55 | 31.54 | 47.44 | 87.49 | 163.36 | 295.71 | 504.29 | 829.36 | 1152.68 | 1547.79 | 1739.79 | 1789.56 | 1501.22 |
| Democratic Republic of the Congo | 4.82 | 6.18 | 8.13 | 13.65 | 23.14 | 39.83 | 62.21 | 153.92 | 219.56 | 385.73 | 469.56 | 645.56 | 523.60 |
| Denmark | 3.94 | 4.72 | 5.80 | 7.66 | 11.13 | 16.60 | 26.42 | 48.87 | 64.83 | 123.06 | 174.79 | 296.70 | 363.95 |
| Djibouti | 25.00 | 31.64 | 40.51 | 64.78 | 102.67 | 169.96 | 255.89 | 601.00 | 862.97 | 1487.55 | 1867.41 | 2353.95 | 1798.32 |
| Dominica | 11.16 | 15.84 | 21.45 | 32.63 | 47.90 | 73.06 | 113.24 | 226.89 | 339.85 | 634.30 | 829.78 | 1194.39 | 1131.63 |
| Dominican Republic | 17.13 | 25.58 | 34.75 | 54.91 | 78.98 | 128.01 | 163.78 | 324.16 | 375.29 | 702.43 | 687.90 | 947.27 | 959.16 |
| Ecuador | 16.78 | 18.90 | 22.61 | 29.11 | 43.82 | 62.04 | 83.70 | 155.58 | 192.11 | 306.97 | 426.64 | 638.72 | 802.06 |
| Egypt | 137.32 | 148.21 | 182.58 | 242.68 | 391.18 | 710.68 | 1047.82 | 1819.32 | 2369.27 | 2986.54 | 3725.00 | 3901.36 | 3020.73 |
| El Salvador | 10.64 | 12.52 | 15.43 | 19.18 | 28.50 | 39.69 | 53.61 | 113.71 | 147.98 | 274.79 | 369.29 | 531.72 | 495.59 |
| Equatorial Guinea | 20.79 | 26.16 | 34.77 | 54.69 | 90.05 | 142.98 | 224.29 | 522.25 | 804.43 | 1471.24 | 1970.44 | 2684.32 | 2297.52 |
| Eritrea | 9.37 | 11.74 | 15.71 | 25.69 | 42.31 | 67.60 | 100.50 | 240.21 | 332.39 | 591.63 | 744.64 | 961.57 | 787.91 |
| Estonia | 2.74 | 3.49 | 4.66 | 6.69 | 11.32 | 18.39 | 31.59 | 56.51 | 82.16 | 104.95 | 130.79 | 150.54 | 157.57 |
| Eswatini | 12.33 | 16.02 | 21.90 | 34.25 | 60.52 | 98.60 | 138.84 | 475.07 | 583.66 | 1024.62 | 1284.54 | 1732.39 | 1614.70 |
| Ethiopia | 4.20 | 5.09 | 6.16 | 8.33 | 11.85 | 18.42 | 28.02 | 68.67 | 105.17 | 199.64 | 272.81 | 400.99 | 343.09 |
| Fiji | 16.50 | 22.33 | 35.45 | 37.75 | 63.77 | 99.04 | 129.31 | 255.72 | 304.52 | 466.64 | 572.87 | 701.00 | 690.97 |
| Finland | 1.69 | 1.83 | 2.20 | 2.79 | 4.12 | 5.94 | 9.45 | 16.60 | 24.84 | 38.69 | 61.88 | 98.16 | 127.58 |
| France | 4.38 | 5.32 | 6.60 | 8.96 | 13.22 | 18.41 | 26.41 | 43.39 | 57.71 | 94.27 | 133.48 | 224.97 | 332.80 |
| Gabon | 22.73 | 28.19 | 37.85 | 62.74 | 106.73 | 174.30 | 272.52 | 637.72 | 932.08 | 1550.09 | 1994.55 | 2587.32 | 2211.01 |
| Gambia | 16.13 | 17.98 | 20.96 | 36.22 | 46.90 | 85.47 | 126.76 | 271.65 | 336.13 | 469.55 | 585.23 | 911.23 | 934.84 |
| Georgia | 16.60 | 24.92 | 33.47 | 47.24 | 90.76 | 141.03 | 206.46 | 432.42 | 599.22 | 1169.41 | 1542.23 | 2039.71 | 1253.69 |
| Germany | 5.76 | 7.08 | 8.85 | 12.12 | 18.72 | 27.90 | 41.52 | 69.76 | 97.26 | 163.85 | 227.94 | 369.01 | 465.73 |
| Ghana | 39.01 | 51.43 | 59.77 | 112.50 | 142.66 | 262.88 | 358.87 | 786.73 | 963.57 | 1351.94 | 1748.85 | 2593.90 | 2404.39 |
| Greece | 6.57 | 7.71 | 8.98 | 11.06 | 15.85 | 21.87 | 31.98 | 67.54 | 107.41 | 242.36 | 418.26 | 847.19 | 1141.17 |
| Greenland | 3.25 | 4.22 | 5.52 | 7.81 | 12.21 | 17.69 | 28.70 | 56.10 | 79.47 | 143.48 | 184.31 | 279.00 | 321.45 |
| Grenada | 14.53 | 23.77 | 34.47 | 50.67 | 78.68 | 127.92 | 205.55 | 412.47 | 617.33 | 994.04 | 1222.18 | 1749.96 | 1608.71 |
| Guam | 10.53 | 16.07 | 26.34 | 25.39 | 38.01 | 58.84 | 72.99 | 110.60 | 134.43 | 175.84 | 217.09 | 267.60 | 234.90 |
| Guatemala | 9.22 | 10.05 | 12.01 | 14.73 | 21.34 | 28.65 | 37.44 | 91.42 | 125.75 | 235.92 | 296.77 | 542.82 | 750.92 |
| Guinea | 9.07 | 10.52 | 11.61 | 20.94 | 26.33 | 49.55 | 71.67 | 159.79 | 191.26 | 276.24 | 333.31 | 557.27 | 504.79 |
| Guinea-Bissau | 14.51 | 16.94 | 19.18 | 35.94 | 45.94 | 81.10 | 119.10 | 247.72 | 288.78 | 396.91 | 462.77 | 721.72 | 678.02 |
| Guyana | 27.41 | 39.14 | 50.61 | 78.19 | 125.54 | 198.26 | 294.70 | 641.66 | 877.48 | 1579.78 | 1668.14 | 2095.48 | 1615.71 |
| Haiti | 6.64 | 10.15 | 14.17 | 24.41 | 36.54 | 60.83 | 86.93 | 180.75 | 240.58 | 462.48 | 550.92 | 777.12 | 663.26 |
| Honduras | 8.88 | 11.32 | 15.91 | 24.78 | 40.75 | 63.85 | 95.29 | 229.56 | 325.00 | 583.00 | 748.27 | 999.88 | 920.53 |
| Hungary | 14.21 | 20.49 | 28.65 | 44.08 | 74.16 | 140.32 | 228.26 | 373.63 | 490.76 | 680.03 | 902.50 | 1139.63 | 1147.75 |
| Iceland | 1.15 | 1.33 | 1.51 | 1.89 | 2.60 | 3.67 | 5.18 | 8.96 | 13.03 | 24.99 | 37.13 | 63.04 | 81.41 |
| India | 17.57 | 22.88 | 30.64 | 41.79 | 69.13 | 122.57 | 200.96 | 438.35 | 600.94 | 929.31 | 1053.88 | 1219.66 | 1115.97 |
| Indonesia | 23.12 | 29.52 | 45.49 | 66.94 | 120.37 | 202.82 | 313.01 | 659.49 | 915.87 | 1574.26 | 1832.01 | 2484.45 | 2276.30 |
| Iran (Islamic Republic of) | 47.94 | 59.94 | 83.04 | 120.62 | 181.32 | 300.50 | 461.06 | 752.81 | 1039.64 | 1468.87 | 2214.79 | 2614.49 | 2620.82 |
| Iraq | 50.51 | 72.93 | 115.74 | 191.70 | 384.32 | 668.32 | 1006.30 | 1909.87 | 2628.71 | 3783.17 | 4680.58 | 5405.53 | 4833.69 |
| Ireland | 2.49 | 2.95 | 3.27 | 4.12 | 5.53 | 7.52 | 10.74 | 18.83 | 28.39 | 59.33 | 99.15 | 177.95 | 250.07 |
| Israel | 8.23 | 9.60 | 10.97 | 14.13 | 20.57 | 29.52 | 42.47 | 75.04 | 103.17 | 190.40 | 285.48 | 490.29 | 612.50 |
| Italy | 6.67 | 7.85 | 9.06 | 10.90 | 14.67 | 19.31 | 27.26 | 48.70 | 73.76 | 151.30 | 253.25 | 492.52 | 762.64 |
| Jamaica | 11.77 | 13.84 | 16.95 | 25.16 | 36.72 | 50.82 | 70.32 | 165.84 | 248.24 | 654.32 | 812.88 | 1173.09 | 953.65 |
| Japan | 7.09 | 8.79 | 12.02 | 16.86 | 25.38 | 36.52 | 52.99 | 84.36 | 116.22 | 171.22 | 235.65 | 338.14 | 472.39 |
| Jordan | 28.05 | 38.04 | 54.16 | 78.63 | 134.12 | 231.18 | 377.72 | 713.16 | 1128.38 | 1528.49 | 2351.50 | 2715.28 | 2639.22 |
| Kazakhstan | 23.82 | 37.31 | 59.60 | 92.58 | 168.37 | 277.42 | 445.62 | 839.99 | 1172.55 | 1745.09 | 2136.24 | 2881.55 | 2694.15 |
| Kenya | 8.55 | 10.50 | 12.85 | 18.06 | 26.93 | 41.41 | 60.96 | 144.92 | 209.74 | 379.71 | 503.62 | 695.52 | 560.48 |
| Kiribati | 11.67 | 21.47 | 35.31 | 32.81 | 57.93 | 78.74 | 107.63 | 194.81 | 212.46 | 318.71 | 377.88 | 436.57 | 355.28 |
| Kuwait | 36.43 | 50.60 | 69.53 | 98.31 | 145.77 | 222.12 | 326.51 | 601.39 | 922.36 | 1452.99 | 1978.19 | 2305.81 | 2377.50 |
| Kyrgyzstan | 17.72 | 26.52 | 44.45 | 70.98 | 160.82 | 261.29 | 418.99 | 874.50 | 1114.04 | 1412.08 | 1535.46 | 1592.30 | 1252.84 |
| Lao People's Democratic Republic | 7.92 | 10.80 | 16.48 | 25.85 | 46.30 | 80.85 | 122.55 | 276.54 | 369.77 | 680.22 | 781.71 | 1046.16 | 873.97 |
| Latvia | 9.08 | 12.56 | 18.52 | 30.97 | 61.45 | 115.67 | 209.51 | 386.87 | 572.16 | 786.20 | 1012.44 | 1331.11 | 1399.99 |
| Lebanon | 30.04 | 38.38 | 54.07 | 74.56 | 104.68 | 153.64 | 232.73 | 364.43 | 494.38 | 729.10 | 1049.98 | 1380.76 | 1414.65 |
| Lesotho | 10.04 | 13.68 | 19.06 | 30.07 | 57.51 | 89.17 | 123.09 | 434.96 | 520.89 | 964.21 | 1150.07 | 1596.34 | 1478.02 |
| Liberia | 11.11 | 12.54 | 13.85 | 22.71 | 29.35 | 51.47 | 74.86 | 161.39 | 199.18 | 289.22 | 370.00 | 621.94 | 604.76 |
| Libya | 78.12 | 98.66 | 141.60 | 189.10 | 288.18 | 403.51 | 565.80 | 995.54 | 1401.29 | 1789.47 | 2395.80 | 2320.66 | 2179.13 |
| Lithuania | 8.39 | 12.37 | 17.00 | 27.59 | 48.28 | 76.65 | 135.71 | 253.52 | 369.48 | 482.27 | 588.70 | 732.92 | 796.38 |
| Luxembourg | 3.97 | 4.74 | 5.43 | 6.75 | 9.50 | 12.81 | 18.76 | 32.81 | 50.48 | 98.48 | 158.43 | 266.26 | 319.11 |
| Madagascar | 5.89 | 7.88 | 10.27 | 16.31 | 25.39 | 40.55 | 59.48 | 136.28 | 188.49 | 324.02 | 388.76 | 501.53 | 377.65 |
| Malawi | 4.17 | 5.13 | 6.34 | 10.30 | 16.60 | 27.91 | 42.25 | 103.31 | 146.39 | 253.37 | 334.10 | 427.80 | 336.40 |
| Malaysia | 18.70 | 24.57 | 33.87 | 46.86 | 77.69 | 127.44 | 187.70 | 336.67 | 456.34 | 769.71 | 869.98 | 1071.28 | 899.71 |
| Maldives | 8.24 | 10.62 | 13.44 | 17.45 | 26.27 | 40.28 | 57.52 | 106.91 | 157.07 | 260.23 | 333.09 | 536.40 | 472.76 |
| Mali | 7.01 | 8.96 | 9.30 | 14.49 | 17.36 | 31.26 | 42.98 | 98.11 | 129.70 | 212.24 | 274.95 | 489.72 | 486.26 |
| Malta | 5.95 | 6.72 | 7.96 | 9.81 | 13.40 | 18.29 | 26.57 | 51.21 | 83.21 | 165.46 | 273.13 | 466.70 | 511.44 |
| Marshall Islands | 14.06 | 22.26 | 36.58 | 36.77 | 58.14 | 79.69 | 102.76 | 191.69 | 228.17 | 351.33 | 429.63 | 548.67 | 500.18 |
| Mauritania | 27.30 | 31.80 | 36.22 | 59.68 | 75.76 | 138.46 | 192.08 | 395.08 | 508.39 | 738.23 | 953.62 | 1488.06 | 1420.71 |
| Mauritius | 14.75 | 19.30 | 26.71 | 33.27 | 52.52 | 76.34 | 100.53 | 228.76 | 308.70 | 552.39 | 694.12 | 937.07 | 813.27 |
| Mexico | 12.18 | 14.27 | 18.35 | 23.78 | 35.48 | 49.94 | 68.71 | 135.46 | 181.95 | 291.26 | 386.92 | 543.56 | 578.86 |
| Micronesia (Federated States of) | 17.13 | 28.07 | 39.51 | 35.86 | 68.97 | 101.90 | 132.86 | 243.87 | 277.58 | 436.89 | 538.52 | 674.52 | 620.50 |
| Monaco | 6.10 | 7.56 | 9.35 | 12.15 | 17.12 | 23.95 | 35.36 | 63.28 | 93.63 | 178.57 | 276.48 | 455.50 | 557.01 |
| Mongolia | 18.25 | 34.58 | 55.99 | 87.25 | 158.81 | 232.49 | 272.13 | 490.35 | 579.54 | 764.28 | 689.55 | 780.76 | 833.19 |
| Montenegro | 11.45 | 15.18 | 21.08 | 29.67 | 46.30 | 79.55 | 138.31 | 279.86 | 443.49 | 756.83 | 1020.68 | 1461.82 | 1585.33 |
| Morocco | 46.05 | 68.89 | 105.66 | 172.99 | 279.60 | 468.86 | 720.57 | 1264.09 | 1775.26 | 2542.99 | 3290.44 | 3759.98 | 3467.63 |
| Mozambique | 3.51 | 4.58 | 6.46 | 12.53 | 22.24 | 39.24 | 63.04 | 154.08 | 210.16 | 311.93 | 379.91 | 410.07 | 269.41 |
| Myanmar | 21.81 | 24.14 | 34.25 | 49.25 | 84.22 | 152.37 | 219.06 | 505.35 | 687.55 | 1296.88 | 1584.76 | 2263.53 | 1942.85 |
| Namibia | 13.05 | 16.31 | 21.76 | 32.22 | 56.96 | 92.70 | 133.63 | 477.05 | 633.25 | 1192.77 | 1518.16 | 2019.63 | 1823.60 |
| Nauru | 16.25 | 24.41 | 39.13 | 38.64 | 60.71 | 81.93 | 98.33 | 173.82 | 196.63 | 291.80 | 349.16 | 403.77 | 349.55 |
| Nepal | 8.56 | 10.95 | 14.89 | 22.91 | 41.00 | 79.72 | 135.23 | 335.75 | 488.57 | 841.04 | 1042.06 | 1123.29 | 1213.91 |
| Netherlands | 5.44 | 6.65 | 8.16 | 10.90 | 16.61 | 23.42 | 34.00 | 57.35 | 80.05 | 147.63 | 222.22 | 379.06 | 505.42 |
| New Zealand | 1.94 | 2.31 | 2.65 | 3.34 | 4.21 | 5.20 | 7.45 | 11.94 | 17.29 | 34.09 | 55.54 | 98.48 | 139.41 |
| Nicaragua | 5.51 | 6.76 | 9.03 | 13.09 | 20.15 | 30.74 | 43.50 | 113.22 | 162.65 | 326.73 | 437.08 | 655.79 | 634.78 |
| Niger | 5.87 | 6.62 | 7.52 | 12.48 | 15.85 | 30.34 | 42.58 | 97.51 | 123.60 | 188.23 | 244.21 | 431.28 | 421.55 |
| Nigeria | 19.41 | 22.61 | 26.41 | 41.83 | 54.06 | 96.52 | 142.18 | 327.55 | 462.40 | 739.84 | 1022.15 | 1559.15 | 1287.31 |
| Niue | 8.83 | 12.54 | 18.87 | 20.23 | 32.27 | 48.48 | 65.38 | 121.02 | 147.55 | 222.39 | 276.63 | 330.92 | 297.20 |
| North Macedonia | 27.91 | 37.75 | 54.93 | 79.71 | 156.35 | 256.16 | 490.33 | 1127.88 | 1979.94 | 3743.25 | 6435.35 | 8733.35 | 9357.32 |
| Northern Mariana Islands | 9.17 | 13.12 | 19.09 | 21.92 | 33.23 | 49.39 | 69.63 | 125.37 | 159.72 | 238.17 | 308.63 | 392.41 | 377.40 |
| Norway | 2.31 | 2.71 | 3.15 | 4.04 | 5.45 | 7.45 | 10.45 | 18.97 | 26.86 | 50.87 | 73.02 | 124.59 | 160.37 |
| Oman | 38.02 | 46.39 | 66.50 | 90.51 | 148.57 | 247.97 | 443.25 | 906.19 | 1569.95 | 2518.39 | 3676.25 | 5358.42 | 3661.31 |
| Pakistan | 21.89 | 31.52 | 39.60 | 59.03 | 95.72 | 162.40 | 251.75 | 588.04 | 811.70 | 1354.89 | 1524.68 | 1600.23 | 1549.27 |
| Palau | 10.79 | 18.96 | 30.62 | 27.63 | 43.64 | 66.10 | 87.40 | 153.46 | 182.04 | 246.15 | 266.02 | 287.73 | 289.09 |
| Palestine | 33.71 | 39.60 | 56.50 | 91.98 | 157.43 | 311.34 | 548.14 | 999.22 | 1654.92 | 2577.41 | 3511.67 | 3980.22 | 3861.18 |
| Panama | 6.54 | 7.76 | 9.50 | 13.50 | 16.54 | 23.17 | 31.56 | 68.61 | 95.54 | 207.79 | 297.81 | 487.51 | 496.50 |
| Papua New Guinea | 4.09 | 6.14 | 10.29 | 11.01 | 18.81 | 28.29 | 38.90 | 75.19 | 92.19 | 146.51 | 179.44 | 236.85 | 206.98 |
| Paraguay | 5.34 | 6.84 | 9.06 | 13.32 | 26.13 | 40.07 | 58.33 | 142.44 | 190.44 | 363.23 | 446.74 | 626.04 | 529.64 |
| Peru | 14.34 | 15.89 | 18.85 | 24.28 | 33.72 | 47.27 | 62.47 | 122.05 | 163.37 | 272.98 | 387.87 | 535.64 | 613.86 |
| Philippines | 16.61 | 21.88 | 31.83 | 44.82 | 78.12 | 120.21 | 171.23 | 326.02 | 421.66 | 658.19 | 727.57 | 948.05 | 777.44 |
| Poland | 15.95 | 21.81 | 30.19 | 46.13 | 80.46 | 129.95 | 208.08 | 375.63 | 525.74 | 779.62 | 1025.20 | 1392.82 | 1585.13 |
| Portugal | 3.29 | 3.88 | 4.81 | 6.26 | 9.00 | 12.47 | 18.59 | 39.13 | 59.60 | 128.52 | 202.75 | 358.75 | 453.88 |
| Puerto Rico | 2.33 | 2.75 | 3.66 | 4.62 | 5.80 | 7.68 | 9.71 | 16.73 | 21.17 | 40.08 | 52.74 | 86.06 | 94.51 |
| Qatar | 27.64 | 36.56 | 53.30 | 67.49 | 103.61 | 141.50 | 199.04 | 333.21 | 535.46 | 973.04 | 1880.58 | 3440.82 | 3796.52 |
| Republic of Korea | 12.23 | 15.89 | 22.48 | 33.43 | 53.88 | 83.35 | 127.87 | 218.00 | 318.53 | 579.35 | 856.89 | 1275.93 | 1500.48 |
| Republic of Moldova | 12.59 | 16.66 | 24.72 | 38.89 | 71.44 | 124.57 | 223.01 | 451.99 | 683.80 | 949.19 | 977.65 | 1069.02 | 813.90 |
| Romania | 14.69 | 19.34 | 27.33 | 41.26 | 79.63 | 127.27 | 204.90 | 443.57 | 645.26 | 1161.25 | 1574.78 | 2102.19 | 2052.40 |
| Russian Federation | 13.71 | 21.03 | 33.38 | 52.16 | 93.13 | 157.63 | 256.52 | 473.21 | 662.44 | 1004.16 | 1264.43 | 1508.55 | 1527.06 |
| Rwanda | 5.86 | 7.12 | 8.77 | 12.93 | 20.09 | 32.86 | 53.26 | 128.51 | 200.49 | 370.77 | 504.37 | 717.48 | 585.51 |
| Saint Kitts and Nevis | 5.93 | 7.73 | 11.33 | 19.27 | 33.38 | 61.59 | 95.07 | 177.68 | 250.06 | 429.02 | 539.35 | 800.33 | 787.51 |
| Saint Lucia | 14.27 | 21.00 | 28.06 | 46.17 | 70.43 | 104.62 | 157.13 | 302.86 | 388.89 | 763.40 | 940.52 | 1421.98 | 1639.58 |
| Saint Vincent and the Grenadines | 16.42 | 23.44 | 30.76 | 49.48 | 79.47 | 115.49 | 158.69 | 292.09 | 384.93 | 794.79 | 1000.40 | 1545.28 | 1491.93 |
| Samoa | 10.70 | 16.56 | 25.91 | 28.51 | 47.49 | 70.85 | 91.89 | 174.30 | 218.16 | 338.09 | 429.74 | 538.99 | 506.05 |
| San Marino | 4.26 | 5.19 | 6.17 | 7.82 | 10.75 | 15.12 | 22.67 | 42.90 | 66.06 | 129.06 | 202.92 | 335.66 | 400.06 |
| Sao Tome and Principe | 28.12 | 30.04 | 35.12 | 56.93 | 73.91 | 126.98 | 181.42 | 360.02 | 477.41 | 697.96 | 860.97 | 1252.97 | 1179.52 |
| Saudi Arabia | 59.19 | 86.43 | 146.04 | 240.35 | 377.67 | 598.59 | 845.00 | 1449.29 | 1916.85 | 2661.14 | 3415.66 | 4235.71 | 4420.32 |
| Senegal | 15.54 | 17.09 | 19.42 | 32.89 | 42.56 | 75.80 | 109.22 | 234.07 | 294.74 | 421.75 | 532.64 | 867.54 | 845.95 |
| Serbia | 21.42 | 31.84 | 44.56 | 69.88 | 132.60 | 241.40 | 434.05 | 809.71 | 1323.34 | 2028.08 | 3126.75 | 5299.18 | 5897.86 |
| Seychelles | 19.54 | 25.79 | 33.78 | 48.06 | 77.03 | 113.72 | 156.27 | 290.74 | 384.78 | 562.01 | 780.83 | 1015.10 | 907.41 |
| Sierra Leone | 12.89 | 14.83 | 16.31 | 28.44 | 35.02 | 64.25 | 88.28 | 189.64 | 234.90 | 328.50 | 405.81 | 655.78 | 601.73 |
| Singapore | 7.58 | 9.11 | 12.03 | 18.50 | 27.61 | 42.13 | 61.79 | 102.90 | 146.16 | 246.99 | 331.28 | 483.34 | 521.44 |
| Slovakia | 14.66 | 21.15 | 30.86 | 49.41 | 79.20 | 124.11 | 205.22 | 386.52 | 563.06 | 822.11 | 1075.82 | 1393.96 | 1278.45 |
| Slovenia | 7.92 | 10.12 | 13.57 | 17.84 | 26.59 | 42.82 | 67.02 | 128.55 | 207.04 | 351.91 | 506.29 | 822.12 | 816.21 |
| Solomon Islands | 5.51 | 9.63 | 19.61 | 20.65 | 40.90 | 62.77 | 80.25 | 151.46 | 172.36 | 281.38 | 344.42 | 437.02 | 390.61 |
| Somalia | 1.27 | 1.57 | 2.10 | 3.64 | 6.01 | 10.10 | 14.94 | 37.34 | 51.83 | 89.13 | 110.72 | 148.52 | 124.99 |
| South Africa | 25.51 | 34.04 | 38.51 | 47.50 | 71.05 | 110.55 | 167.70 | 514.78 | 663.42 | 1040.16 | 1239.52 | 1882.97 | 1873.97 |
| South Sudan | 5.84 | 7.11 | 9.04 | 13.24 | 20.62 | 34.15 | 52.04 | 126.40 | 191.52 | 341.00 | 452.71 | 610.75 | 467.41 |
| Spain | 3.81 | 4.48 | 5.14 | 6.48 | 9.02 | 12.89 | 18.24 | 30.36 | 41.05 | 75.47 | 115.55 | 214.60 | 313.23 |
| Sri Lanka | 16.59 | 20.82 | 27.55 | 36.77 | 58.60 | 91.20 | 129.67 | 253.99 | 381.76 | 663.70 | 880.17 | 1242.76 | 1370.37 |
| Sudan | 55.39 | 80.80 | 115.34 | 178.58 | 279.08 | 469.16 | 677.77 | 1182.80 | 1603.66 | 2288.33 | 2983.43 | 3464.12 | 2953.09 |
| Suriname | 17.41 | 25.21 | 37.11 | 51.40 | 78.28 | 124.67 | 181.81 | 368.73 | 518.50 | 869.66 | 1085.17 | 1420.26 | 1085.32 |
| Sweden | 3.08 | 3.40 | 3.64 | 4.25 | 5.29 | 6.82 | 8.89 | 13.59 | 19.39 | 33.14 | 51.57 | 95.05 | 120.92 |
| Switzerland | 3.12 | 3.71 | 4.26 | 5.42 | 7.43 | 10.38 | 15.02 | 26.83 | 38.43 | 67.95 | 107.78 | 196.98 | 278.56 |
| Syrian Arab Republic | 55.43 | 58.17 | 81.95 | 110.50 | 178.39 | 299.38 | 433.83 | 820.48 | 1061.26 | 1481.75 | 1845.77 | 2425.33 | 2725.75 |
| Taiwan (Province of China) | 18.08 | 22.97 | 28.63 | 36.62 | 53.04 | 74.20 | 110.62 | 189.61 | 265.76 | 437.04 | 573.81 | 840.03 | 794.26 |
| Tajikistan | 11.84 | 17.26 | 22.11 | 30.83 | 49.29 | 79.94 | 132.60 | 346.48 | 540.80 | 1161.02 | 1358.48 | 1521.16 | 1055.94 |
| Thailand | 23.42 | 33.05 | 45.70 | 57.93 | 87.87 | 129.56 | 173.37 | 288.66 | 378.82 | 556.33 | 686.78 | 878.52 | 754.14 |
| Timor-Leste | 7.70 | 10.31 | 14.84 | 21.71 | 40.66 | 72.28 | 111.57 | 236.63 | 321.85 | 573.46 | 700.67 | 941.44 | 823.20 |
| Togo | 16.28 | 18.60 | 20.40 | 36.70 | 47.92 | 87.29 | 129.10 | 264.72 | 319.34 | 427.16 | 540.36 | 844.65 | 745.92 |
| Tokelau | 7.66 | 11.62 | 15.28 | 19.54 | 25.82 | 38.55 | 47.58 | 85.03 | 106.33 | 156.60 | 212.78 | 250.04 | 228.78 |
| Tonga | 10.12 | 14.34 | 19.57 | 22.55 | 33.86 | 48.91 | 69.55 | 134.14 | 186.23 | 294.75 | 366.57 | 456.90 | 423.66 |
| Trinidad and Tobago | 19.65 | 30.69 | 40.19 | 56.11 | 75.20 | 106.80 | 147.45 | 303.13 | 433.29 | 798.58 | 1025.39 | 1355.19 | 1174.67 |
| Tunisia | 33.47 | 50.54 | 72.64 | 107.08 | 160.19 | 252.78 | 415.17 | 710.63 | 1045.70 | 1535.68 | 2115.70 | 2752.37 | 2549.56 |
| Turkey | 17.99 | 23.73 | 33.60 | 46.98 | 75.47 | 117.55 | 172.95 | 327.52 | 497.49 | 862.67 | 1240.83 | 1590.88 | 1523.44 |
| Turkmenistan | 51.35 | 64.89 | 101.97 | 148.19 | 309.22 | 473.29 | 720.74 | 1527.59 | 1781.34 | 2712.54 | 2319.12 | 2366.73 | 1507.66 |
| Tuvalu | 9.80 | 15.13 | 24.65 | 24.06 | 38.20 | 53.24 | 65.76 | 120.40 | 142.78 | 216.76 | 269.70 | 334.78 | 306.29 |
| Uganda | 6.15 | 7.42 | 9.28 | 14.10 | 21.86 | 37.06 | 54.57 | 132.71 | 196.99 | 354.99 | 487.41 | 667.68 | 517.29 |
| Ukraine | 25.86 | 37.64 | 59.29 | 91.02 | 151.82 | 248.31 | 389.22 | 657.22 | 903.78 | 1210.98 | 1421.08 | 1544.29 | 1624.59 |
| United Arab Emirates | 52.37 | 79.12 | 152.02 | 208.37 | 376.93 | 576.80 | 834.77 | 1417.37 | 1852.55 | 2213.27 | 3427.21 | 4117.52 | 3683.16 |
| United Kingdom | 4.28 | 5.13 | 5.96 | 7.72 | 10.47 | 14.20 | 19.61 | 34.15 | 47.31 | 91.98 | 142.55 | 258.53 | 372.93 |
| United Republic of Tanzania | 6.07 | 7.40 | 9.14 | 13.96 | 21.96 | 36.39 | 55.65 | 149.92 | 230.85 | 396.82 | 509.67 | 640.90 | 441.18 |
| United States of America | 5.07 | 6.08 | 7.10 | 8.85 | 11.86 | 16.38 | 23.23 | 38.74 | 49.45 | 81.57 | 110.26 | 177.59 | 210.15 |
| United States Virgin Islands | 3.77 | 5.14 | 7.33 | 11.89 | 18.71 | 30.43 | 44.09 | 80.62 | 106.87 | 185.66 | 225.80 | 336.31 | 325.72 |
| Uruguay | 5.16 | 6.30 | 8.89 | 13.11 | 20.16 | 32.55 | 50.82 | 98.96 | 140.27 | 229.38 | 297.33 | 439.29 | 470.27 |
| Uzbekistan | 34.33 | 52.44 | 81.13 | 136.90 | 287.81 | 497.15 | 735.15 | 1604.25 | 1842.59 | 2838.21 | 3128.30 | 3760.07 | 3090.53 |
| Vanuatu | 11.16 | 18.74 | 32.48 | 32.92 | 56.90 | 81.78 | 105.48 | 201.98 | 242.06 | 364.66 | 442.77 | 551.05 | 525.98 |
| Venezuela (Bolivarian Republic of) | 11.24 | 12.28 | 15.35 | 20.40 | 29.85 | 45.25 | 67.26 | 163.18 | 229.24 | 488.36 | 662.97 | 957.78 | 750.42 |
| Viet Nam | 15.13 | 21.13 | 32.47 | 51.51 | 99.81 | 173.14 | 274.76 | 554.70 | 750.84 | 1376.83 | 1699.55 | 2295.46 | 1940.18 |
| Yemen | 35.28 | 54.27 | 89.49 | 151.81 | 240.29 | 403.60 | 579.87 | 1040.50 | 1388.15 | 1942.09 | 2459.57 | 2848.28 | 2500.89 |
| Zambia | 9.39 | 11.74 | 15.51 | 25.23 | 42.70 | 70.75 | 113.58 | 282.65 | 406.12 | 725.43 | 914.02 | 1242.68 | 973.47 |
| Zimbabwe | 8.71 | 11.75 | 15.95 | 23.35 | 40.81 | 71.39 | 98.31 | 266.07 | 306.31 | 498.16 | 654.72 | 841.28 | 808.48 |

**Supplementary Table 7 Death Cases, DALYs and Increased Proportion of Ischaemic Stroke Attributed to Ambient Particulate Matter Pollution in Men and Women from 1990 to 2019 in Different Regions Around the World**

| **Region** | **Female** | | | | | | **Male** | | | | | |
| --- | --- | --- | --- | --- | --- | --- | --- | --- | --- | --- | --- | --- |
|  | **Death Cases** | | | **DALYs** | | | **Death Cases** | | | **DALYs** | | |
|  | **1990** | **2019** | **Increased Proportion** | **1990** | **2019** | **Increased Proportion** | **1990** | **2019** | **Increased Proportion** | **1990** | **2019** | **Increased Proportion** |
| Global | 118423.82 | 232449.11 | 1.96 | 2435589.19 | 5348572.44 | 2.20 | 107183.61 | 283508.06 | 2.65 | 2506566.26 | 6554599.59 | 2.61 |
| Andean Latin America | 260.78 | 671.67 | 2.58 | 5884.70 | 14453.51 | 2.46 | 338.06 | 698.04 | 2.06 | 7311.72 | 14691.97 | 2.01 |
| Australasia | 176.90 | 120.03 | 0.68 | 3088.22 | 2003.88 | 0.65 | 136.49 | 87.55 | 0.64 | 2747.45 | 1582.20 | 0.58 |
| Caribbean | 453.25 | 930.89 | 2.05 | 9139.58 | 17727.10 | 1.94 | 450.77 | 886.26 | 1.97 | 9640.83 | 18502.68 | 1.92 |
| Central Asia | 2049.00 | 3514.52 | 1.72 | 45719.36 | 84797.90 | 1.85 | 1765.26 | 3627.60 | 2.05 | 44683.77 | 95382.55 | 2.13 |
| Central Europe | 12205.48 | 10509.75 | 0.86 | 236321.84 | 179810.73 | 0.76 | 9964.47 | 8126.42 | 0.82 | 222428.74 | 163926.40 | 0.74 |
| Central Latin America | 1306.14 | 2478.09 | 1.90 | 30893.20 | 53733.50 | 1.74 | 1426.46 | 2534.29 | 1.78 | 32020.25 | 53847.86 | 1.68 |
| Central sub-Saharan Africa | 111.34 | 650.65 | 5.84 | 2983.83 | 16044.50 | 5.38 | 155.28 | 651.66 | 4.20 | 3924.30 | 16933.11 | 4.31 |
| East Asia | 18816.01 | 100485.31 | 5.34 | 478211.21 | 2465875.57 | 5.16 | 27290.25 | 148626.74 | 5.45 | 685226.46 | 3369042.75 | 4.92 |
| Eastern Europe | 31749.55 | 15882.68 | 0.50 | 600964.22 | 278462.23 | 0.46 | 16811.33 | 10710.10 | 0.64 | 394441.16 | 243569.75 | 0.62 |
| Eastern Sub-Saharan Africa | 180.80 | 1125.31 | 6.22 | 4822.14 | 28117.13 | 5.83 | 252.51 | 1366.34 | 5.41 | 6376.62 | 33964.23 | 5.33 |
| High-income Asia Pacific | 4842.73 | 4700.30 | 0.97 | 98384.66 | 91446.33 | 0.93 | 4677.68 | 4615.65 | 0.99 | 105495.71 | 100812.41 | 0.96 |
| High-income North America | 4950.36 | 2078.70 | 0.42 | 104257.43 | 48212.14 | 0.46 | 3307.59 | 1362.59 | 0.41 | 77268.66 | 38117.63 | 0.49 |
| North Africa and Middle East | 6807.97 | 23800.47 | 3.50 | 178376.06 | 629843.94 | 3.53 | 7564.50 | 24563.48 | 3.25 | 198778.88 | 647360.53 | 3.26 |
| Oceania | 6.52 | 27.81 | 4.27 | 234.43 | 952.75 | 4.06 | 12.53 | 41.01 | 3.27 | 386.88 | 1283.65 | 3.32 |
| South Asia | 4407.82 | 32474.99 | 7.37 | 106976.51 | 737929.80 | 6.90 | 8165.14 | 42916.99 | 5.26 | 195910.98 | 986125.68 | 5.03 |
| Southeast Asia | 3552.89 | 16258.58 | 4.58 | 94145.35 | 387265.02 | 4.11 | 4678.64 | 18733.34 | 4.00 | 123188.30 | 476154.15 | 3.87 |
| Southern Latin America | 909.36 | 1020.84 | 1.12 | 17784.71 | 18907.61 | 1.06 | 950.78 | 943.13 | 0.99 | 20243.19 | 18709.68 | 0.92 |
| Southern sub-Saharan Africa | 541.14 | 1819.86 | 3.36 | 13435.42 | 40239.53 | 3.00 | 499.70 | 1303.35 | 2.61 | 13232.42 | 33099.83 | 2.50 |
| Tropical Latin America | 1593.23 | 2369.10 | 1.49 | 35248.30 | 46307.45 | 1.31 | 2275.60 | 2792.25 | 1.23 | 55400.77 | 60251.77 | 1.09 |
| Western Europe | 22712.85 | 8023.12 | 0.35 | 350263.56 | 116056.58 | 0.33 | 15718.31 | 5716.32 | 0.36 | 288779.27 | 98973.94 | 0.34 |
| Western sub-Saharan Africa | 789.70 | 3506.45 | 4.44 | 18454.45 | 90385.24 | 4.90 | 742.27 | 3204.95 | 4.32 | 19079.90 | 82266.82 | 4.31 |

**Supplementary Table 8 Death Cases, DALYs and Increased Proportion of Ischaemic Stroke Attributed to Ambient Particulate Matter Pollution in Men and Women from 1990 to 2019 in Countries of Different SDI Levels Around the World**

| **SDI** | **Female** | | | | | | **Male** | | | | | |
| --- | --- | --- | --- | --- | --- | --- | --- | --- | --- | --- | --- | --- |
|  | **Death Cases** | | | **DALYs** | | | **Death Cases** | | | **DALYs** | | |
|  | **1990** | **2019** | **Increased Proportion** | **1990** | **2019** | **Increased Proportion** | **1990** | **2019** | **Increased Proportion** | **1990** | **2019** | **Increased Proportion** |
| High SDI | 29547.66 | 15868.51 | 0.54 | 531585.91 | 336504.20 | 0.63 | 22514.38 | 15883.40 | 0.71 | 475530.54 | 374207.89 | 0.79 |
| High-middle SDI | 61743.04 | 80215.84 | 1.30 | 1220469.15 | 1700031.46 | 1.39 | 47863.62 | 90058.70 | 1.88 | 1106868.53 | 1995487.29 | 1.80 |
| Middle SDI | 21515.68 | 96486.53 | 4.48 | 546617.87 | 2374672.68 | 4.34 | 27732.22 | 124795.64 | 4.50 | 704654.90 | 2954231.69 | 4.19 |
| Low-middle SDI | 4544.86 | 33302.36 | 7.33 | 109969.64 | 772795.74 | 7.03 | 7302.84 | 44073.53 | 6.04 | 176242.14 | 1018156.46 | 5.78 |
| Low SDI | 1028.66 | 6489.51 | 6.31 | 26085.99 | 162648.96 | 6.24 | 1730.16 | 8609.14 | 4.98 | 42382.47 | 210431.20 | 4.97 |
